# Protocol for a multicentre randomised controlled parallel-group trial to compare the effectiveness of remotely delivered cognitive-behavioural and graded exercise interventions with usual care alone to lessen the impact of fatigue in inflammatory rheumatic diseases (LIFT)

**DOI:** 10.1136/bmjopen-2018-026793

**Published:** 2019-01-30

**Authors:** Kathryn R Martin, Eva-Maria Bachmair, Lorna Aucott, Emma Dures, Richard Emsley, Stuart R Gray, Sarah Hewlett, Vinod Kumar, Karina Lovell, Gary J Macfarlane, Graeme MacLennan, Paul McNamee, John Norrie, Lorna Paul, Stuart Ralston, Stefan Siebert, Alison Wearden, Peter D White, Neil Basu

**Affiliations:** 1 Epidemiology Group, University of Aberdeen, Aberdeen, UK; 2 Aberdeen Centre for Arthritis and Musculoskeletal Health, University of Aberdeen, Aberdeen, UK; 3 Centre of Healthcare and Randomised Trials (CHaRT), Health Service Research Unit, University of Aberdeen, Aberdeen, UK; 4 Department of Nursing and Midwifery, University of the West of England, Academic Rheumatology Unit, Bristol Royal Infirmary, Bristol, UK; 5 Department of Biostatistics and Health Informatics, Institute of Psychiatry, Psychology and Neuroscience, King’s College London, London, UK; 6 Institute of Cardiovascular and Medical Sciences, BHF Glasgow Cardiovascular Research Centre, University of Glasgow, Glasgow, UK; 7 Department of Rheumatology, Ninewells Hospital, NHS Tayside, Dundee, UK; 8 School of Health Sciences, University of Manchester, Manchester, UK; 9 Health Economics Research Unit, University of Aberdeen, Aberdeen, UK; 10 Edinburgh Clinical Trials Unit, University of Edinburgh, Western General Hospital, Edinburgh, UK; 11 School of Health and Life Science, Glasgow Caledonian University, Glasgow, UK; 12 Rheumatology and Bone Disease, University of Edinburgh, Western General Hospital, Edinburgh, UK; 13 Institute of Infection, Immunity and Inflammation, University of Glasgow, Glasgow, UK; 14 School of Health Sciences, University of Manchester, Manchester, UK; 15 Department of Psychological Medicine, Queen Mary University of London, Bartholomew’s Hospital, London, UK

**Keywords:** lift, fatigue, inflammatory rheumatic disease, randomised controlled trial, cognitive behavioural, exercise

## Abstract

**Introduction:**

Fatigue remains pervasive, disabling and challenging to manage across all inflammatory rheumatic diseases (IRDs). Non-pharmacological interventions, specifically cognitive-behavioural approaches (CBAs) and graded exercise programmes designed to support and increase exercise, are valuable treatments which help patients with IRD to manage their fatigue. Yet, healthcare systems have encountered substantial barriers to the implementation of these therapeutic options. Lessening the Impact of Fatigue in Inflammatory Rheumatic Diseases: a Randomised Trial (LIFT) is designed to give insights into the effectiveness of a remotely delivered standardised intervention for a range of patients with IRD. It will also enable the exploration of putative moderating factors which may allow for the future triage of patients and to investigate the precise mediators of treatment effect in IRD-related fatigue.

**Methods and analysis:**

LIFT is a pragmatic, multicentre, three-arm randomised, controlled trial, which will test whether adapted CBA and personalised exercise programme interventions can individually reduce the impact and severity of fatigue. This will be conducted with up to 375 eligible patients diagnosed with IRD and interventions will be delivered by rheumatology healthcare professionals, using the telephone or internet-based audio/video calls.

**Ethics approval and dissemination:**

Ethical approval has been granted by Wales REC 7 (17/WA/0065). Results of this study will be disseminated through presentation at scientific conferences and in scientific journal. A lay summary of the results will be sent to participants.

**Trial registration number:**

NCT03248518; Pre-results.

**Strengths and limitations of this study**

- Lessening the Impact of Fatigue in Inflammatory Rheumatic Diseases: a Randomised Trial is designed to give insights into the effectiveness of a remotely delivered standardised intervention for a range of patients with inflammatory rheumatic disease (IRD).
- It will also enable the exploration of putative moderating factors which may allow for the future triage of patients and to investigate the precise mediators of treatment effect in IRD-related fatigue.
- Although participants and therapist are not blinded to treatment allocation, all research staff at study sites performing assessments are blinded.
- The availability of rheumatology healthcare professionals to be trained as therapists for the study may be restricted.
- Patients responding to study invitation may not represent the entire population, however, we seek to mitigate this by considering all patients, with a relevant diagnosis, managed at each recruiting hospital.

## Introduction

Despite major advances in the management of inflammatory rheumatic diseases (IRDs), patients remain burdened by their disease and cite fatigue as a principal problem. In rheumatoid arthritis (RA), for example, as many as 80% of patients report significant fatigue^1^ and over 70% consider fatigue to be equal to pain in terms of burden.^2^ Moreover, fatigue is a crucial determinant of impaired quality of life^3 4^ and a predictor of work disability.^5 6^ Indeed, over 75% of patients identify fatigue as the main barrier to remaining in employment.^7^ Studies in other chronic IRDs, such as axial spondyloarthritis (AxSpA) and systemic lupus erythematosus (SLE), have reported similar fatigue prevalence of 66%–85%^8 9^ and report the impact of fatigue on quality of life and employment to be equally pronounced.^10–12^ In spite of these profound consequences, patients feel that this symptom is clinically neglected^13 14^ and rheumatologists admit uncertainty regarding its management.^15^

This situation reflects the poor availability of suitable interventions within traditional healthcare systems such as the National Health Service (NHS), but this is not to say that effective treatments do not exist. There is now a considerable consensus across the healthcare community that non-pharmacological interventions, specifically cognitive-behavioural approaches (CBAs) and programmes designed to support increased exercise, are valuable treatments which help patients with IRD to manage the fatigue associated with their chronic diseases.^16 17^

Our current team has made key contributions to the evidence base, which supports the use of these treatments for fatigue in IRDs.^18–20^ However, current healthcare systems pose to substantial barriers to the implementation of these therapeutic options as part of standard clinical care.

First, existing studies—including our own—have only developed bespoke disease-specific models of care, which vary in content, structure and method of delivery. Inevitably, this necessitates the development of multiple particular skill sets and duplicated pathways for the care providers if they are to equitably serve their diverse patient populations—a time consuming, costly and inefficient undertaking.

Second, patients find it challenging to commit to regular face-to-face treatment sessions (a common underpinning of existing CBA and exercise interventions). This is often due to a combination of health complications and the time-constrained nature of modern life, particularly relevant to those patients still in employment.

Third, individual patients report substantial variation in their preference and response to the distinct interventions of CBA and exercise.^21 22^

### Rationale for study

It is becoming increasingly clear that:

- Similarities exist across chronic IRDs regarding the nature and likely mechanisms which maintain fatigue—such as dysfunctional activity behaviours^23–25^ and illness beliefs^14 26 27^and so the application of standardised generic, rather than disease-specific, interventions may prove effective.
- Alternative, more flexible, methods of remote delivery such as telephone and internet-based audio/video calls can be just as effective as traditional face-to-face interventions.^28 29^
- In the future, the identification of baseline patient preferences and characteristics which can predict differential treatment effects (moderators) will be vital to inform a personalised triage approach to care.^22 30^

Therefore, a pragmatic study is proposed, which will use CBA and personalised exercise programme (PEP) interventions, informed by previously developed interventions^31 32^ which will be generically applicable across IRD-related fatigue populations. We will test whether these two key non-pharmacological interventions compared with usual care alone can individually reduce fatigue, when delivered by the rheumatology team, across a mixture of IRDs using telephone or internet-based audio/video calls. In doing so, this will enable us to explore potential moderating factors which may allow for the future triage of patients according to the most suitable intervention, as well as to investigate the precise mediators of the effect of treatment on IRD-related fatigue.

### Objectives

Primarily, we seek to test the hypothesis that either standardised CBA or PEP interventions, in addition to usual care, is more effective than usual care alone to lessen the impact and severity of fatigue. We will also explore the underlying moderators (in order to inform future patient triage) and mediators (in order to optimise future interventions) of IRD-related fatigue.

## Methods and analysis

### Study description

**Figure 1:**
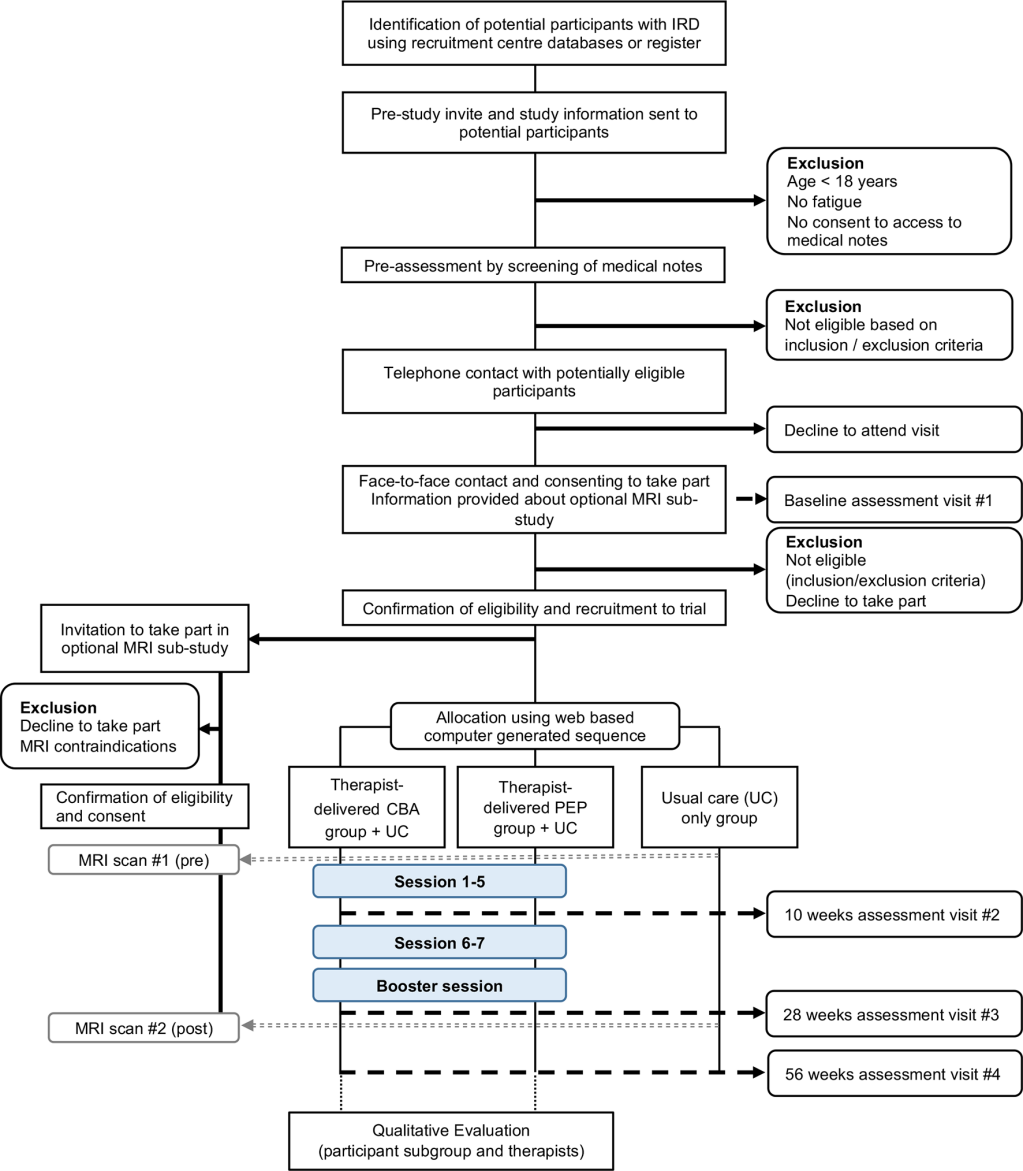
Study flow chart. Patients diagnosed with IRD will be invited by post and asked to return the prestudy screening questions. Screening is a two-step process using screening questions and review of medical records to establish fatigue state and identify other exclusion criteria. Potential participants will be contacted by phone to verify eligibility criteria and invited to the baseline assessment visit. After obtaining informed consent, their eligibility is confirmed (including fatigue states as well as determination of TSH, Hb and eGFR values if these were not available in the medical notes), baseline data are collected and participants are randomised into the study. At baseline, participants are also given a participant information sheet about the MRI substudy. Randomised participants will be contacted by the Trial Office in Aberdeen if they are also eligible to take part in the MRI substudy. CBA, cognitive-behavioural approach; eGFR, estimated glomerular filtration rate; Hb, haemoglobin; IRD, inflammatory rheumatic disease; PEP, personalised exercise programme; TSH, thyroid stimulating hormone.

Lessening the Impact of Fatigue in Inflammatory Rheumatic Diseases: a Randomised Trial (LIFT) is a multicentre, three-arm randomised controlled trial testing usual care with additional adapted CBA or PEP interventions versus usual care alone. Eligible participants will be identified from approximately 3600 patients with IRD attending major secondary care rheumatology services in the UK (figure 1). Recruitment for the study began in August 2017. We anticipate recruiting 375 participants previously diagnosed with IRD.

**Figure 2:**
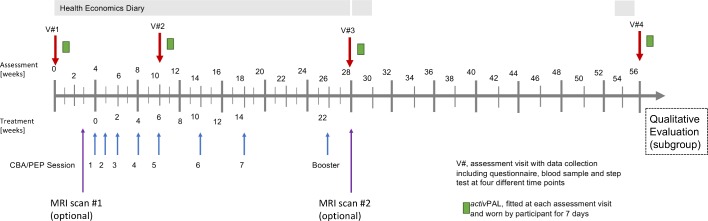
Timeline intervention and assessment. CBA, cognitive-behavioural approach; PEP, personalised exercise programme.

The timeline for assessment and delivery of interventions is summarised in figure 2. We anticipate that active CBA and PEP interventions will start between 2 and 8 weeks post-randomisation, with an average delay of 4 weeks after randomisation. Follow-up data will be collected from all participants at 10 weeks, 28 weeks and 56 weeks after randomisation adjusted for the average delay of 4 weeks, therefore all participants will remain part of the study for 13 months.

### Participant recruitment and eligibility

Enrolment is by invitation only and all potential participants will undergo a two-step screening process consisting of a prestudy invite and an assessment of their hospital medical notes. Patients with rheumatologist diagnosed IRDs (eg, RA, SLE and AxSpA, psoriatic arthritis, vasculitis or Sjögren’s syndrome) will receive a mailed prestudy invite consisting of a cover letter (see online supplementary file 1 for details on study within a trial), study information and questions to explore interest and eligibility.

10.1136/bmjopen-2018-026793.supp1 Supplementary data

After return of the required prestudy screening questions, the research personnel at each site will assess their medical notes to determine eligibility based on the inclusion and exclusion criteria (table 1).

**Table 1:** Inclusion and exclusion criteria

In order to be considered eligible for participation in the study they must:	
Criterion	Characteristics of eligible participants
1.	Be male or female aged ≥18 years at the time of consent.
2.	Have been diagnosed with an IRD such as RA, SLE or AxSpA by a consultant rheumatologist.
3.	Report fatigue to be a persistent problem as evidenced by answering both questions: Have you had problems with fatigue for more than 3 months? (Yes). Please circle the number that shows your average level of fatigue during the past 7 days. (≥6 based on a numerical rating scale of 0 (no fatigue) to 10 (totally exhausted)).
4.	Have access to a telephone landline or mobile telephone and/or internet-based audio/video calls.
5.	Give permission for researchers to access their hospital medical notes.
6.	Have stable disease as evidenced by no change in immunomodulatory therapy within the last 3 months based on hospital medical records.
7.	Currently be under the care of a secondary care physician.

Participants will be excluded if:	
Criterion	Characteristics of ineligible participants
1.	There are significant abnormalities in thyroid function (TSH levels) in the most recent blood test done within the last 3 months.
2.	There is evidence of severe anaemia (haemoglobin levels) in the most recent blood test done within the last 3 months.
3.	There is evidence of severe renal dysfunction (eGFR) in the most recent blood test done within the last 3 months.
4.	They have a medical condition which would make the proposed interventions unsuitable, for example, significant heart disease.
5.	They are pregnant.
6.	They are unable to understand English sufficiently to take part in the intervention.
7.	They are unable to provide written informed consent.
8.	They are not willing to be randomised.
9.	They are currently participating in an interventional clinical trial.

AxSpA, axial spondyloarthritis; eGFR, estimated glomerular filtration rate; IRD, inflammatory rheumatic disease; RA, rheumatoid arthritis; SLE, systemic lupus erythematosus; TSH, thyroid stimulating hormone.

A trained rheumatology research nurse will review and confirm eligibility. In addition, the local rheumatology consultant will have the opportunity to withdraw or exclude participants. If a participant has been identified as potentially eligible, she/he will be invited to an appointment for a baseline assessment visit at the local study site.

### Consenting participants

Patients will make a final decision to participate when they attend the local study site for the baseline assessment visit. No study-specific procedures will take place before written consent has been obtained. At the baseline assessment visit, a designated member of the local research team will confirm eligibility (including reconfirmation of fatigue state). It will also be determined whether the participant has ever met the relevant classification criteria for RA (mandatory, 2010 American College of Rheumatology (ACR)/European League Against Rheumatology (EULAR)^33^ or 1987 ACR^34^) or other IRDs (non-mandatory; SLE 1997 ACR SLE criteria,^35^ Assessment of SpondyloArthritis international Society (ASAS criteria for AxSpA^36^).

### Randomisation

Participants will be allocated to receive either of the two treatments or usual care alone (1:1:1 ratio) using a computer-generated sequence. Randomisation will be minimised by diagnosis (RA, SLE, or AxSpA or other IRD) and the presence/absence of depressive symptoms (Hospital Anxiety and Depression Scale (HADS)^37^ subscale score >10) and will include a random element set at 20%.

### Blinding

Full blinding will not be possible due to the need for participants to engage in specific behavioural change interventions. However, to reduce detection bias, we will aim to ‘blind’ research personnel undertaking outcome assessments to participants’ treatment allocation. To facilitate blinding, we will remind participants to refrain from discussing (and subsequently revealing) their treatment allocation at follow-up assessments with research personnel. Finally, all data will be analysed blind to allocation.

### Interventions and treatment protocol

#### Usual care

All participants will receive usual care and will receive a Versus Arthritis (formerly Arthritis Research UK) education booklet for self-management of fatigue^38^ by post. It represents usual care in almost all UK rheumatology centres and is freely available. The booklet covers the major relevant topics (including fatigue validation, energy management, priorities, sleep, stress and assertiveness) underpinned by goal setting and self-monitoring of activity. It encourages at several key points that the patient asks their rheumatology team for support to work through the booklet. We will not restrict what usual care may involve, but will monitor the care received for all participants as part of our health economics analysis.

Participants randomised to the active treatments will also receive either CBA or PEP. The CBA and PEP treatments are adapted from previous fatigue-specific cognitive behavioural^31 32^ and exercise interventions^31^ to ensure that they are suitable for a remote delivery via telephone or internet-based audio/video call and applicable to the broad spectrum of IRD.

#### Cognitive-behavioural approach

CBA is a structured psychological intervention, which explicitly aims to replace unhelpful beliefs and behaviours with more adaptive ones. In this study, the CBA will target a number of unhelpful behavioural patterns such as ‘activity avoidance’ and ‘all or nothing’. These can lead to negative mood states, which exacerbates fatigue even further. Following a brief assessment of individual beliefs and behaviours surrounding fatigue, the aim of the treatment is to change unhelpful beliefs and behavioural factors through the application of participant-centred strategies and behavioural activities, which are supported by written materials and regular consultations with rheumatology healthcare professionals. The participants will receive additional leaflets and diaries to assist them with making changes to manage fatigue.

#### Personalised exercise programme

PEP is based on the premise that chronic fatigue relates to exercise intolerance, supported by unhelpful illness beliefs (such as fear avoidance) and deconditioning, with a consequent increase of effort (perceived or otherwise). PEP aims to disrupt this vicious cycle by a graded exposure behavioural therapy, which is symptom contingent, to gradually optimise patients’ levels of exercise with a view to modifying their altered perception of effort, improve their tolerance of exercise, fitness and function, reverse the deconditioning and ultimately reduce the severity and impact of fatigue. In this study, participants will receive an individually tailored graded exercise programme, delivered according to their physical capacity and gradually increased in duration and then intensity. The participants will receive additional information and diaries to assist with the intervention. The intervention will use pedometers and/or heart rate monitors to enhance motivation. Overall, the times and duration of exercise will be recorded in exercise diaries during the intervention.

Both CBA and PEP interventions will be delivered by healthcare professionals. PEP may be delivered by a rheumatology specialist physiotherapist and CBA may be delivered by a rheumatology nurse or an equally qualified and trained allied health professional (eg, occupational therapist) who are members of local NHS staff. Participants will be offered seven one-to-one telephone or internet-based audio/video call (based on patient preference) sessions (up to 45 min) of CBA or PEP interventions over a period of 14 weeks with a trained therapist (figure 2). The first session of PEP, however, will be delivered face-to-face. A booster session will be conducted at 22 weeks after the start of therapy by the relevant healthcare professional.

#### Training of healthcare professionals as therapists before the study

Separate CBA and PEP training will be provided for the NHS staff delivering the interventions. This will comprise an intensive 2-day group course delivered by experienced designated investigators supplemented with the therapist manuals. The course will use a range of methods including skills practice with specific feedback using fictitious but typical fatigued cases.

#### Supervision and support of therapists during the study

Supervision will be provided by designated investigators on a fortnightly basis, or as required, to the therapists either face-to-face or by telephone depending on feasibility and preference. In addition, support will be available in cases where a therapist requires assistance with respect to a particular participant. In addition to the option to contact the supervisor directly, we will have a notification system incorporated in the database.

#### Treatment fidelity

Some of the intervention sessions will be recorded and used in supervision to provide feedback to therapists and to ensure treatment fidelity. We aim to take a 5% sample of intervention sessions for those in CBA and PEP from participants who agree to be recorded which is based on a random sample generated from an algorithm that takes into account session number, therapist, site location, patient gender. This is equivalent to approximately 89 recordings per intervention and will be subject to treatment adherence (no sessions completed) and participant permission to record sessions.

### Outcome measures

**Figure 3:**
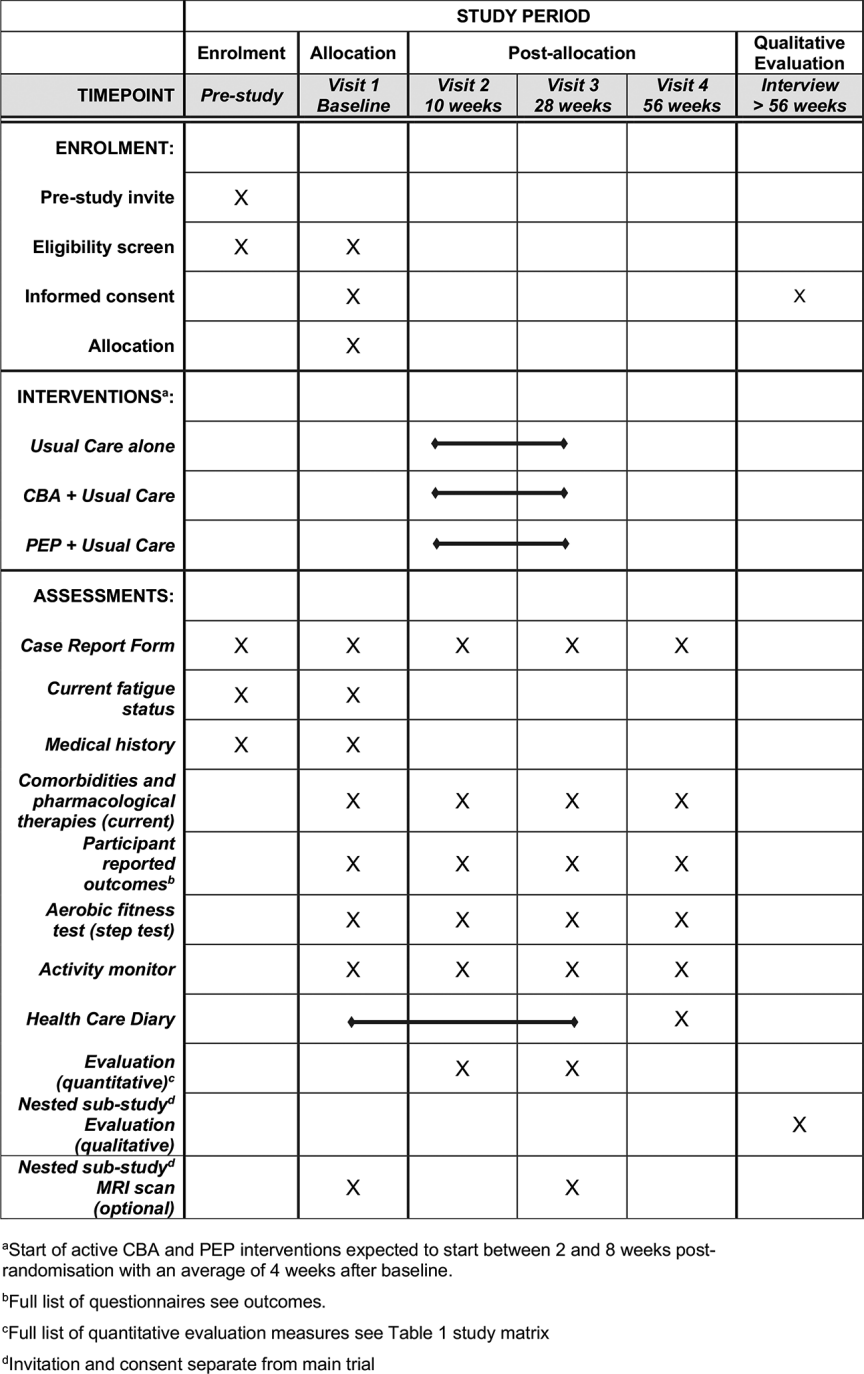
Standard Protocol Items: Recommendations for Interventional Trials (SPIRIT) schedule of enrolment, interventions and assessments. CBA, cognitive-behavioural approach; PEP, personalised exercise programme.

All outcome and mediator measures will be assessed at randomisation (baseline) and then, on average, 10 weeks, 28 weeks and 56 weeks thereafter (by questionnaire unless stated) in all participants (figure 3). The outcome measures at each time point and their source (ie, questionnaire, medical record, blood sample, diary) are summarised in a study matrix (online supplementary table 1).

10.1136/bmjopen-2018-026793.supp2 Supplementary data

#### Primary outcome measures

The primary outcome, fatigue, is measured with the Chalder Fatigue Scale (CF)^39^ which assesses the physical and mental symptoms of fatigue using Likert scoring, and the Fatigue Severity Scale (FSS)^40^ assessing the impact of fatigue at 56 weeks post randomisation. If the effect of the intervention is positive on the CF, then the FSS outcome will be formally analysed. Should the intervention have no effect on the CF, then an explorative analysis of the FSS data will be performed.

#### Secondary outcome measures

Secondary outcomes are:

Fatigue: Bristol Rheumatoid Arthritis Fatigue MultiDimensional Questionnaire^41^ assessing the physical, living, cognition and emotional aspects of fatigue.

Quality of life and Health Utility Index: Short Form-12 (SF-12)^42^ assessing functional health and well-being from the participant’s perspective.

Pain: Pain numerical rating scale^43^ assessing pain intensity.

Anxiety and depression: HADS.^37^

Sleep: Sleep problem scale.^44^

Impact on work: Work Productivity and Activity Impairment Questionnaire: Specific Health Problem.^45^

Impact on activities: Short form of Valued Life Activities Scale.^46^

Global outcome: Change of global health.

#### Mediator/moderator measures

While many secondary outcome measures may also function as mediators, or their baseline values as moderators, more detailed cognitive, behavioural, clinical and physical data will be collected in order to fully characterise these factors.

Cognitions and behaviours: Brief Illness Perception Questionnaire,^47^ Behavioural Response to Illness Questionnaire.^48^

Clinical: Presence of fibromyalgia,^49^ serological status, erosive status, disease duration, previous and current pharmacological therapies, disease activity: self-reported using a Numeric Rating Scale, Disease Activity Score 28 (DAS28) for RA (mandatory), other disease-specific activity measures (non-mandatory), inflammation (C-reactive protein (CRP)/Erythrocyte sedimentation rate (ESR), presence of other comorbidities (Charlson Comorbidity Index).^50^

Physical: Physical activity and sedentary behaviour profiles will be measured by an activity monitor (*activ*PAL, Paltechnologies, Glasgow). The *activ*PAL will be fitted to the participant at each assessment visit and participants will be instructed to remove the device and post it back to the research team after 7 days in the stamped addressed envelope provided to them.

Quantifying aerobic fitness: A step test, which involves participants wearing a heart rate monitor and stepping onto a 10-inch high box for 3 min at different stepping rates will assess aerobic fitness. Participants stop the test if their heart reaches 65% of predicted maximal heart rate (220-age or 190-age if the participant is prescribed beta-blocker) at the end of any stage. One minute of rest is given between stages and maximal oxygen uptake is estimated from heart rate recordings according to established equations.^51^ In addition, values of Borg Rating of Perceived Exertion are collected.

Neuroimaging data: Participants will have an option to undertake a multimodal MRI brain scan (see online supplementary file 2).

10.1136/bmjopen-2018-026793.supp3 Supplementary data

Furthermore, participants will be given the option to provide additional blood samples which will be stored in a designated freezer at the University of Aberdeen. These will be a maximum of three tubes per visit of 1x PAXgene DNA (visit 1 only), 1x PAXgene RNA (visit 1, 2, 3), 1x serum (visit 1, 2, 3). Additional consent for their use in future unspecified studies will be obtained.

### Quantitative process evaluation

Participant preference: Participants will be given a short synopsis of all three treatments, usual care, CBA and PEP interventions as treatment for IRD-related fatigue at baseline. They will then be asked about which treatment they would choose if they had a choice, as well as about their strength of preference.

Participant adherence: Adherence to the interventions will be monitored via attendance records kept by the therapists. In addition, participants receiving the CBA or PEP interventions will be contacted by a member of Trial Office in Aberdeen at time of session 4 (approximately week 8) and session 8 (approximately week 26) for a telephone interview. They will be asked to indicate on a scale from 0 (not at all) to 10 (completely), if they think that this treatment is the right approach and their willingness to engage and adhere to the intervention. At the same time, the therapists will be asked during supervision to what extent they think that the participant has engaged with treatment and adhered to the agreed actions and plans.

Intervention acceptance: Acceptance by participants will be evaluated in all three treatment arms using the Client Satisfaction Questionnaire^52^ at 28 weeks.

### Qualitative evaluation

A subgroup of participants will be invited to take part in a nested qualitative evaluation study. We will conduct qualitative evaluations of both participants who received CBA or PEP after they completed the 56-week follow-up and all therapists who will deliver the interventions. To ensure integrity, participants and therapists will be invited to take part once they have left or completed the study. Data will be collected through semistructured interviews conducted by telephone or by internet-based audio/video calls. In addition to the practical considerations of offering options, this approach acknowledges that both participants and therapists will have been involved in a remotely delivered intervention. All interviews will be audio recorded, anonymised during transcription and checked for accuracy.

Participant experiences of the interventions will be analysed using a framework analysis^53^ to assess the content, mode of delivery, acceptability, barriers and facilitators, helpfulness and subsequent impact of the interventions on their daily lives. We will use a maximum variation sampling strategy^54^ to include participants with a range of IRDs, gender, age, disease duration and primary outcomes in the randomised controlled trial. To achieve this, a minimum of 40 interviews is required. All selected participants will receive additional information after they have completed the last assessment visit (week 56) and provide separate written informed consent for the qualitative component.

All therapists will be invited to take part in a telephone interview to explore experiences of intervention training and delivery, including challenges and benefits of learning and using new skills, and barriers and facilitators to supporting patients remotely using a structured manual. This evaluation will be analysed using inductive thematic analysis.^55^ All therapists will receive additional information after they have either left the study or after the intervention phase and provide separate written informed consent for the qualitative component.

### Patient safety

There are unlikely to be major safety issues with our proposed non-pharmacological interventions. However, if the therapists delivering CBA and PEP or any study personnel have any safety concerns, we will follow a specific standard operating procedure for adverse events in non-Clinical Trial of an Investigational Medicinal Product (non-CTIMP) studies.

### Laboratory and sample analysis

This multicentre study involves a number of standard blood investigations, that is, thyroid-stimulating hormone (TSH), haemoglobin, serum creatinine, CRP and ESR, which are routinely processed by local NHS laboratories. Blood samples for CRP and ESR analysis and sample storage for future ethically approved research will be taken at each assessment as specified in the study matrix by trained personnel only following established procedures. Blood samples for TSH, haemoglobin and serum creatinine analysis will only be taken at baseline if they are required to confirm eligibility. Sample coding, preparation, storage, analysis and transfer of results and optional blood samples for long-term storage at the trial centre will be performed according to the analytical protocol based on national laboratory guidelines. The exact logistic and procedure will be agreed with each site before the start of the study and every effort will be made to standardise the workflow across sites to reduce bias. Additional optional blood samples will be stored for future ethically approved research. All study blood results (abnormal or otherwise) will also be directed towards the local principal investigator (PI) who can determine locally how these will be handled.

### Withdrawal procedures

Participants will have the option to withdraw at any time during the study period of 13 months. The participant needs to request this formally and a withdrawal document will be completed and signed by the designated research staff at the local sites. They have the option to either withdraw from the study completely or from parts of it (prestudy invite, treatment or follow-up). If participants withdraw from the study completely or from follow-up assessments, they will not receive further invitations but we will use the data collected prior to the withdrawal (depending on permission). Those withdrawing from the treatment only will continue to be sent invitations to attend and complete follow-up assessment visits, unless they request to withdraw completely later on. Failure of any participant to complete a follow-up at any particular time point will not be counted as a withdrawal unless the participant formally requests to withdraw. In addition, a participant can also be withdrawn by others, for example, the local PI or primary consultant, should the need arise at any stage throughout the study period. This also includes loss of capacity for ongoing consent.

### Statistical issues

#### Sample size

Our planned primary intention-to-treat analyses will compare PEP+usual care versus usual care alone, and CBA+usual care versus usual care alone separately. We base our calculations on a standardised effect size of 0.50 (considered credible in other pragmatic effectiveness studies). This would equate to being powered to detect a minimal important clinical difference of 2 units in the CF Scale, assuming a common SD across the randomised groups of 4 units. Assuming an overall significance level of 5% (by calculating the two prespecified randomised groups comparisons, PEP+usual care vs usual care alone and CBA+usual care vs usual care alone, at 2.5%, to maintain an overall level of not more than 5%) and a power of 90%, we require 100 evaluable participants in each of the three groups.

The data of participants are evaluable when outcomes at the 56 weeks follow-up are available. Based on our own previous studies, we estimate a drop-out rate of 20%, and therefore, we anticipate recruiting 375 participants randomly allocating 125 into each treatment group.

#### Statistical analysis

All statistical analyses will be governed by a comprehensive statistical analysis plan, which will be authored by the trial statistician and approved by the trial steering committee (TSC) and the data monitoring committee before the main study outcome data are examined. All analyses will be carried out using standard statistical software. In accordance with Consolidated Standards of Reporting Trials guidelines,^56^ we will report all participant flow through the study. Descriptive statistics of recruitment, drop-out and completeness of interventions will be provided.

#### Effectiveness analysis

The main effectiveness analysis will be via intention-to-treat including all participants, with no planned interim analysis for early termination for either overwhelming evidence of effectiveness or abandoning for futility. Baseline characteristics will be presented by randomised group without formal statistical tests. We will test the primary hypothesis for between-group change in the primary outcome for each of the two prespecified comparisons (CBA+usual care vs usual care alone and PEP+usual care vs usual care alone) using repeated measures mixed model, with subject as a random effect, and a suitably specified covariance structure (eg, autoregressive[1] (AR[1]), therapist as a random effect (to adjust for any clustering by therapist), with baseline outcome measure, and any other strongly predictive baseline measures, including the minimisation factors of presence/absence of depression and centre. Treatment and its interaction with time will be fitted as fixed effects, and we will apply standard regression diagnostics. The analysis will use statistical techniques for handling missing outcome data using multiple imputation under a missing at random (MAR) assumption. The secondary outcomes will be analysed using an analogous method. The main estimate of treatment effect will focus on the 56 weeks after baseline.

#### Mediation analysis

We will use modern causal inference methods to investigate the set of mediator measures. If the effectiveness analysis shows significance between-group differences in the mediators, then we will use parametric regression models to test for the effect of mediator on an outcome and the residual direct effect of treatment on outcome. Since all the measures are continuous, the indirect effects will be calculated by multiplying relevant pathways and bootstrapping will be used to produce valid SEs for the indirect effects. All analyses will adjust for baseline measures of the mediators, outcome and putative measured confounders. Mediation analyses are potentially biased by measurement error in mediators and hidden confounding between mediators and outcomes; we will build on our previous methodological and applied work in this context to include repeated measurement of mediators and outcomes to account for classical measurement error and baseline confounding.

#### Moderation analysis

We will examine differential treatment effects using the set of moderator measures by extending the intention-to-treat analysis models to include an interaction term between intervention and each of the moderators separately. We will use bias correction/cross-validation methods to identify robust evidence for individual moderation and for a moderation index, both on the overall effect and also along the steps of the mediation pathway.

Every effort will be made to ensure data collection is complete. However, we will use statistical techniques for handling missing outcome data. Multiple imputation under an MAR assumption will be used in the first instance, with additional sensitivity analysis if the MAR assumption is not satisfied.

### Health economics evaluation

An economic evaluation will be conducted from both a healthcare system and societal perspective. Participants will be asked to record, in a diary, all types and duration of hospital admissions, a frequency of visits to the hospital for outpatient attendances and other visits to or from relevant health professionals (eg, general practitioners, nurse practitioners, physiotherapists) and specify whether the main reason for the visit was fatigue. Each participant will be asked to keep the diaries between the baseline and third assessment visit (approximately 28 weeks). Furthermore, they will be asked to keep diaries for 2 weeks after the third assessment visit and 2 weeks before they return for the last assessment visit. National sources of unit cost data will be applied to value resource use (Healthcare Resource Group (HRG) Reference Costs, Unit Costs of Health and Social Care). The costs associated with a delivery of the interventions will be estimated using records kept by therapists of the number and duration of calls per participant.

Participants will be asked to report any contacts with private practitioners, and the costs of over-the-counter medication/complementary therapies purchased. This also includes additional expenses related to their condition or fatigue as well as if there was an impact on paid and unpaid work.

Health-related quality of life data will be collected using the SF-12 and these data will be converted to the quality of life weights using published tariffs. These data will then be used to calculate quality-adjusted life years. As the intervention may affect general well-being as well as reduce fatigue, we will also collect values for changes in well-being data using the Investigating Choice Experiements for the preference of older people CAPability measure for Adults (ICECAP) instrument^57^ and changes in life satisfaction.^58^

### Study management and conduct

The study will be coordinated by a trial management group, consisting of the grant holder chief investigator (CI), additional members of the research group, a study coordinator and a representative from Centre for Healthcare and Randomised Trials (CHaRT).

A TSC has been established. The TSC comprises an independent chair who has expertise in trials and other members, both independent and study investigators, with a background relevant to IRD or type of interventions including a lay representative, who has lived experience of IRD-related fatigue, and a clinician working with people with IRD.

An independent data monitoring committee has been established to oversee study progress. The data monitoring committee comprises a chair who has expertise in trials, a biostatistician and two other independent members with a rheumatology background.

### End of study

The end of study is defined as last data collection of either the qualitative evaluation study phase or during the last follow-up visit at 56 weeks from the last participant after CBA/PEP intervention or usual care start date —whichever comes last.

### Data management, protection, storage and archiving of study documents

All investigators and study staff involved with this study will comply with the requirements of the Data Protection Act 2018 with regard to the collection, storage, processing and disclosure of personal information and will uphold the Act’s core principles. The investigators and study staff will also adhere, if appropriate, to the current version of the NHS Scotland Code of Practice on Protecting Patient Confidentiality. Access to collated participant data will be restricted to the CI and appropriate study staff as needed. All laboratory specimens, evaluation forms, reports and other records will be identified using unique participant ID numbers to maintain participant confidentiality. All records will be kept in a secure storage area with limited access to study staff only. Personal data, including postal address, phone numbers (ie, landline and mobile), email addresses and anonymised data files for study outcomes are stored in locked filing cabinets (hard copy) and in a bespoke database provided and maintained by CHaRT as well as secured shared drives with access via password-controlled computers (university and NHS networks) by study staff only (electronic data).

All study documentation will be kept for a minimum of 5 years from the protocol defined end of study point in the University of Aberdeen archive. When the minimum retention period has elapsed, study documentation will not be destroyed without permission from the sponsor.

### Patient and public involvement

Patients and their carers were involved in the development of the research question and the prioritisation of fatigue as a subject for research. We have benefited from collaborations with representatives of the National Rheumatoid Arthritis Society, the National Ankylosing Spondylitis Society and Lupus UK who have significantly influenced the trial design. In addition, we have worked with patient advisory groups which provided valuable input into the content and layout of the information material and other documents that will be given to the participants to assess the burden of the intervention and data collection visits. Although patients were not actively involved in the recruitment, they are involved in the conduct of the study as lay representatives on the TSC. After completion of the study, the main results and conclusions of the study will be disseminated to participants in lay language in form of a newsletter.

## Discussion

The LIFT trial is a pragmatic multicentre, three-arm randomised controlled trial. It has been designed to test the effectiveness and cost-effectiveness of remotely delivering two non-pharmacological interventions, CBA and PEP, over a period of 22 weeks so as to answer whether treatment can move beyond face-to-face disease-specific interventions for IRD-related fatigue. In order to achieve the study aims, the CBA and PEP interventions were informed by previous evidence-based cognitive behavioural and exercise interventions^31 32^ for fatigue to ensure suitability across IRD conditions, as well as to ensure that both interventions could be delivered via telephone or internet-based audio/video calls by a range of rheumatology healthcare professionals trained as therapists. To reduce selection bias, all patients with a relevant diagnosis, managed at each recruiting site who report fatigue as a persistent problem will be considered, either through database screening or manual screening by research nurses, and invited to take part in the trial. Participants will be asked to provide information about fatigue, our primary outcome, at baseline and weeks 10, 28 and 56 using the CF and FSS to measure severity and impact at critical times throughout the trial. In addition, detailed information on cognitions and behaviours (eg, illness perception), clinical features (eg, disease activity, presence of fibromyalgia), as well as physical activity and fitness levels will allow for the identification of potential moderators and mediators of the treatment effect in this trial. Although complete blinding is not possible due to the provision of an active behavioural change intervention, we aim to reduce detection bias by asking participants to refrain from revealing their treatment allocation to the research nurses undertaking their outcome assessments.

Fatigue remains a significant patient-identified priority, as well as a significant challenge to manage clinically. To provide the necessary evidence to bring about change in health services consistently offered for IRD-related fatigue across the spectrum of IRD conditions, both a quantitative and qualitative evaluation will also be undertaken within the trial. Trial findings will be used to inform future triage of patients into the most suitable intervention, as well as provide evidence of which mediating factors play a role in treatment effect among patients with IRD-related fatigue.

### Ethics and dissemination

The study will be conducted in accordance with the principles of Good Clinical Practice.

Extension of recruitment for 6 months was approved and implemented as minor amendment 09 on 17 September 2018 (protocol V.7, current). Management approval from all NHS health boards was obtained as required by the ethics committee before the start of the study.

Consent will be sought for the recording of interventions sessions for quality assurance and treatment fidelity. Written consent will be obtained to record interviews and to include anonymised statements in the publication of the qualitative evaluation outcomes.

Ownership of the data arising from this study resides with the University of Aberdeen.

A clinical study report will be prepared in accordance with International Council for Harmonisation (ICH) authorship guidelines which will be used for dissemination of findings via the publication and presentation at scientific meetings. Investigators have the right to publish orally or in writing the results of the study.

There are no plans to place study outcome data in a repository at the current time.

### Trial status

The LIFT study began recruitment in August 2017 and will be ongoing until March 2019 (anticipated). It is expected that data collection will be completed by April 2020.

## Supplementary Material

Reviewer comments

Author's manuscript
