# Supplementary material for: Protocol for a multicentre randomised controlled parallel-group trial to compare the effectiveness of remotely delivered cognitive-behavioural and graded exercise interventions with usual care alone to lessen the impact of fatigue in inflammatory rheumatic diseases (LIFT)
Source: BMJ Open. 2019 Jan 30;9(1):e026793. doi: 10.1136/bmjopen-2018-026793 (PMC6359876; doi:10.1136/bmjopen-2018-026793)
Supplement: Supplementary data [file bmjopen-2018-026793supp001.pdf]

Additional File 1 SWAT 24

Background

Recruiting and retaining participants for randomised trials can be extremely difficult. It is likely that less than 50% of trials meet their recruitment target, or meet their target without extending the length of the trial [1-3]. Moreover, poor recruitment can lead to an underpowered study, which may report clinically relevant effects to be statistically non-significant. A non-significant finding increases the risk that an effective intervention will be abandoned before its true value is established, or that there will be a delay in demonstrating this value while more studies or meta-analyses are done. Moreover, if non-responses to the study invitation differ between the patients with diagnosis of RA, SLE and AxSpA, a systematic bias may be introduced that may undermine confidence in the results of the trial. Finally, poor recruitment and subsequently retention can lead to a trial being extended, increasing costs.

Trialists recognise the challenge and use many interventions to improve recruitment and retention but it is generally difficult to predict their effect. The Cochrane systematic review of strategies to improve recruitment [4] and the Cochrane review of strategies it improve retention [5] both found only a handful of interventions with high quality evidence of benefit. Given how central recruitment and retention are to all trials, it is crucial that more rigorous evaluations of recruitment and retention interventions are done.

Rationale for SWAT

One way of doing this is to do a Study Within A Trial (SWAT) [6]. A SWAT provides a protocol for the evaluation of an intervention to improve some part of the trial process, such as recruitment or retention. This evaluation is then embedded within a host trial, such as LIFT. Several teams can follow the same SWAT protocol, meaning the results can be combined in a meta-analysis. This coordinated and collaborative approach means trialists will have faster access to high-quality evidence to inform their trial design, conduct, analysis and reporting decisions.

The SWAT 24 which describes the use of a theory-based cover letter was initially developed to increase response rates of questionnaires sent during follow-up to collect outcome data direct from participants. A low response rate to these questionnaires puts the validity and generalisability of the trial results in jeopardy. Since returning the questionnaire is a behaviour, this opens up the possibility of designing a behaviour change intervention to influence the willingness of participants to do that behaviour. We propose to use SWAT 24 in LIFT to improve response rates to the pre-study invitation letter used to make initial contact with potential participants identified as described in the main protocol to explore interest and eligibility. The SWAT 24 study is part of the Trial Forge initiative to improve trial efficiency [7].

Objective for SWAT 24

To assess the effects of a theory-based cover letter on response rate to a pre-study invite to explore interest and eligibility

Outcome

Primary outcome:

Response rate

Secondary outcomes:

Response time

Consent rate

Study retention

Intervention adherence

Intervention

The Theoretical Domains Framework (TDF) is a tool for identifying theoretical targets for behaviour change interventions [8]. The TDF and behaviour change techniques were used by the IQuaD trial team [9] to produce a template that trial teams can use to structure a theory-informed cover letter.

Comparator

A standard cover letter

Method for allocating to intervention or comparator

Participating study centres will be randomly allocated to send the standard letter or the theory-informed letter.

Analysis plan

The primary analysis is the difference in primary and secondary outcomes between those receiving the theory-based cover letter and those receiving the standard cover letter.

References

1. Sully BGO, Julious SA, Nicholl J. A reinvestigation of recruitment to randomised, controlled, multicenter trials: a review of trials funded by two UK funding agencies. *Trials* 2013, 14(1): 166.
2. McDonald AM, Knight RC, Campbell MK, Entwistle VA, Grant AM, Cook JA, Elbourne DR, Francis D, Garcia J, Roberts I, Snowdon C. What influences recruitment to randomised controlled trials? A review of trials funded by two UK funding agencies. *Trials* 2006;7(1):9.
3. Foy R, Parry J, Duggan A, Delaney B, Wilson S, Lewin-van den Broek NTh, Lassen A, Vickers L, Myres P. How evidence-based are recruitment strategies for randomized controlled trials in primary care? Experience from seven studies. *Family Practice* 2003;20(1):83-92.
4. Treweek S, Mitchell E, Pitkethly M, Cook J, Kjeldstrøm M, Johansen M, Taskila TK, Sullivan F, Wilson S, Jackson C, Jones R, Lockhart P. Strategies to improve recruitment to randomised controlled trials. The Cochrane Library. 2010.

5. Brueton VC, Tierney J, Stenning S, Harding S, Meredith S, Nazareth I, Rait G. Strategies to improve retention in randomised trials. The Cochrane Database of Systematic Reviews. 2013;12:1.
6. Smith V, Clarke M, Devane D, Begley C, Shorter G, Maguire L. SWAT 1: what effects do site visits by the principal investigator have on recruitment in a multicentre randomized trial? *J Evid Based Med* 2013; 6(3):136–7.
7. Treweek S, Altman DG, Bower P, Campbell M, Chalmers I, Cotton S, et al. Making randomised trials more efficient: report of the first meeting to discuss the Trial Forge platform. *Trials* 2015; 16(1):261.
8. Michie et al. Making psychological theory useful for implementing evidence based practice. *BMJ Quality & Safety.* 2005; 14(1):26–33
9. Clarkson JE, Ramsay CR, Averley P, Bonetti D, Boyers D, Campbell L, Chadwick GR, Duncan A, Elders A, Gouick J, Hall AF et al. IQuaD dental trial; improving the quality of dentistry: a multicentre randomised controlled trial comparing oral hygiene advice and periodontal instrumentation for the prevention and management of periodontal disease in dentate adults attending dental primary care. *BMC Oral Health.* 2013; 13(1):58.
