# Supplementary material for: Protocol for a multicentre randomised controlled parallel-group trial to compare the effectiveness of remotely delivered cognitive-behavioural and graded exercise interventions with usual care alone to lessen the impact of fatigue in inflammatory rheumatic diseases (LIFT)
Source: BMJ Open. 2019 Jan 30;9(1):e026793. doi: 10.1136/bmjopen-2018-026793 (PMC6359876; doi:10.1136/bmjopen-2018-026793)
Supplement: Supplementary data [file bmjopen-2018-026793supp002.pdf]

Table 1. Summary of outcome measures for each time point and their source (Study Matrix)

	Source	Items		**Proposed assessment [weeks]**			
			S	0	10	28	56
**Demographic data**							
Date of birth, gender, marital status, employment status, level of education	Q	5		✓			
**Characteristics of study population**							
Overall health	Q	1		✓			
Physical activity (typical self-reported)	Q	1		✓			
Experience of fatigue for more than 3 month	Q / CRF	1	✓	✓			
Average level of fatigue	Q / CRF	1	✓	✓			
Thyroid function test	B or MR			✓			
Urea and electrolytes	B or MR			✓			
Full blood count	B or MR			✓			
Serological status	MR			✓			
Erosive status	MR			✓			
Disease duration	MR			✓			
Presence of other co-morbidities (Charlson Index)	MR/CRF			✓	✓	✓	✓
History of suicide attempts	MR/CRF			✓			
Inflammation (CRP/ESR)	B			✓	✓	✓	✓
Current pharmacological therapies	MR			✓	✓	✓	✓
Blood pressure	T			✓			
**Primary Outcome**							
Chalder Fatigue Scale (Likert scoring)	Q	11		✓	✓	✓	✓
Fatigue Severity Scale	Q	9		✓	✓	✓	✓

	Source	Items		Proposed assessment [weeks]			
			S	0	10	28	56
**Secondary Outcomes**							
Bristol Rheumatoid Arthritis Fatigue Multi-Dimensional Questionnaire (fatigue)	Q	20		✓	✓	✓	✓
Hospital anxiety and depression scale (anxiety and depression)	Q	14		✓	✓	✓	✓
Short Form-12	Q	12		✓	✓	✓	✓
Pain numerical rating scale	Q	1		✓	✓	✓	✓
Sleep problem scale	Q	4		✓	✓	✓	✓
Work Productivity and Activity Impairment Questionnaire	Q	6		✓	✓	✓	✓
Valued Life Activities Scale (short 14 items)	Q	14		✓	✓	✓	✓
Global outcome	Q	1			✓	✓	✓
**Additional mediator/moderator data**							
Cognitions and behaviours							
Brief Illness Perception Questionnaire	Q	9		✓	✓	✓	✓
Behavioural Response to Illness Questionnaire	Q	21		✓	✓	✓	✓
Clinical							
Presence of fibromyalgia	CRF	8		✓			✓
Disease activity (self-reported)	CRF	2		✓	✓	✓	✓
Disease activity DAS28 for RA (mandatory), other disease specific activity measures (non-mandatory)				✓	✓	✓	✓
Physical							
Physical activity profiles, over a 7 day period	T			✓	✓	✓	✓

	Source	Items		**Proposed assessment [weeks]**			
			S	0	10	28	56
Quantifying aerobic fitness (step) test (weight, VO_2 max and Borg Rating of Perceived Exertion)	T			✓	✓	✓	✓
Neuroimaging (optional)							
Multi-modal MRI scan	SC			x		x	
**Quantitative evaluation**							
Patient preference	Q	2		✓			
Patient adherence (attendance records)	SR			x	x	x	
Patient engagement and adherence (telephone call)	Q	3			x	x	
Patent engagement and adherence (therapist perspective)	Q	2			x	x	
Patient acceptability (Client Satisfaction Questionnaire)	Q	8				✓	
**Qualitative process evaluation**							
Qualitative evaluation in participants (5% sample)	I						
Qualitative evaluation in therapists (all)	I						
**Economic evaluation**							
Health care costs from participants per diaries	D			✓	✓	✓	✓
Additional (personal) costs for participant per diaries	D			✓	✓	✓	✓
Cost associated with delivery of therapy	SR			✓	✓	✓	✓
Health related quality of life using SF-12 for calculation of QALY				✓	✓	✓	✓
Changes in well-being data using ICECAP	Q	5		✓	✓	✓	✓
Changes in life satisfaction	Q	1		✓	✓	✓	✓

	Source	Items		**Proposed assessment [weeks]**			
			S	0	10	28	56
**Optional blood sample for future ethically approved research**							
1 x PAXgene RNA				✓	✓	✓	
1 x PAXgene DNA				✓			
1 x serum				✓	✓	✓	

Key: B, blood sample taken specifically for LIFT; **CRF**, part of case report form completed by research nurse during visit; **D**, separate diary for participant to keep; **I**, interviews performed after the last follow-up visit; **ICECAP**, Investigating Choice Experiments for the preference of older people CAPability measures for adults; **MR**, information extracted from medical record; **Q**, data derived from questionnaire; **QALY**, Quality Adjusted Life Years; **S**, information obtained during pre-study invite; **SC**, Scanner; **SR**, information extracted from LIFT study records and logs; **T**, test done specifically for LIFT; **x**, outcome collection at same time frame but separate from assessment visit
