# Supplementary material for: Protocol for a multicentre randomised controlled parallel-group trial to compare the effectiveness of remotely delivered cognitive-behavioural and graded exercise interventions with usual care alone to lessen the impact of fatigue in inflammatory rheumatic diseases (LIFT)
Source: BMJ Open. 2019 Jan 30;9(1):e026793. doi: 10.1136/bmjopen-2018-026793 (PMC6359876; doi:10.1136/bmjopen-2018-026793)
Supplement: Supplementary data [file bmjopen-2018-026793supp003.pdf]

Appendix 2: Delineating the neural mediators of inflammatory rheumatic disease (An optional LIFT sub-study)

Background

One of the objectives of the LIFT trial is to understand the mediators of treatment effect which will enable optimising of interventions and inferences to be made regarding the mechanisms of fatigue. We are collecting putative patient reported, clinical and physical activity profile mediator data. In addition we would like to provide the option for participants to provide their neuroimaging data by undertaking additional MRI scans of their brain.

Neuroimaging has provided consistent mechanistic insights into fatigue, reinforcing our epidemiological investigations of RA related fatigue which have identified strong associations with central factors such as mental health and cognitive dysfunctions but not peripheral measures of inflammation[4,5]. We were the first to test these methods in a fatigued chronic inflammatory disease cohort. We identified multiple neural correlates of fatigue in patients with vasculitis using different structural and functional magnetic resonance imaging (MRI) modes [6,7] and we have since observed similar fatigue specific findings in RA.

Our original studies were small (n=12) and cross-sectional but did implicate a potential role for a striato-thalamo-frontal network. Among fatigued vasculitis patients, we observed an apparent overuse of the cingulum and fornix white matter tracts, as identified by Diffusion Tensor Imaging (DTI), which coincided with the high functional activity within some of their source and destination grey matter structures, specifically the thalamus, medial globus pallidus, medial frontal and cingulate gyri and paracentral lobule.

Our most recent pilot was larger and although uncontrolled included a repeat multi-modal scan at 6 months that enabled the longitudinal evaluation of fatigue in the context of standard care (which did not include fatigue specific therapy). Of those n=54 completing follow up, n=22 reported modest, albeit clinically relevant, improvements in their fatigue. Interestingly, widespread reductions in cortical grey matter volumes were measured, using voxel-based morphometry (VBM), among the non-improvers at follow-up however no such volume changes were observed among improvers. In contrast, sub-cortical grey matter volumes exhibited large significant increases in non-improvers. The sub-cortical grey matter volumes of improvers also increased, although the changes were small in comparison.

In terms of white matter integrity, as measured by DTI, widespread abnormalities were observed among improvers. Within this group, significantly reduced fractional anisotropy (FA) was measured at follow-up compared to baseline in major white matter tracts. Strikingly, no significant longitudinal FA changes were measured within the non-improvers group. Similarly, improvers- and not non-improvers- evidenced widespread imbalances in functional connectivity imbalance over time.

Overall, these data strongly associate central neural pathways in RA related fatigue. Although the different MRI metrics provide complementary evidence which implicate frontal networks, a targeted approach (e.g. with non-invasive neuromodulation devices) will demand much greater knowledge of the precise culprit frontal regions, moreover several non-frontal regions seem also to be relevant. Until now, no studies have been adequately designed to pin-point those brain regions which mediate changes in fatigue (and so demonstrate causal potential).

The problem with our existing studies is that they were either cross-sectional, or longitudinally followed individuals who had spontaneous changes in their fatigue (thus the changes in fatigue from one time point to the next were modest). We propose that the best way to identify which of these brain regions are most important in mediating fatigue is to perform controlled longitudinal imaging studies in individuals prior to and then following an intervention that reliably improves fatigue in most individuals.

This Lessening the Impact of Fatigue Trial (LIFT) sub-study provides a timely opportunity to address this research void.

Sub-study objective

- Which functional and structural brain mediators best explain fatigue improvement and are they potentially trans-cranially accessible?

Study design

An optional nested mechanistic observational sub-study within the Lessening the Impact of Fatigue Trial (LIFT).

Participants

All consenting LIFT participants will be invited to participate in this optional sub-study which involves an additional MRI brain scan prior to and 26 weeks after their first treatment session (if CBA or PEP) or within 8 weeks of randomisation and 6 months thereafter if usual care. The only additional exclusion criteria is any contra-indications to MRI scanning (e.g. pacemaker).

Participant selection

At the baseline visit, all participants will be provided a Participant Information Sheet on this sub-study. The research team will then contact the participants a few days later to establish interest and the absence of MRI contraindications. If suitable and interested, the participant will be offered an appointment to attend their nearest participating MRI research facility (Glasgow, Edinburgh or Aberdeen) within a month. A research team member, recorded in the Delegation Log and with GCP training, will be responsible for taking additional full written informed consent (i.e. specific to this sub-study) on attendance at the imaging centre prior to the MRI assessment and then conduct a final MRI safety screen.

Participant withdrawal

All participants will be free to withdraw at any time from the MRI sub-study, without giving reasons and without prejudicing further treatment or their participation in the LIFT trial.

MRI assessment

Prior to entering the scanner, subjects will have the opportunity to practice a cognitive task required for the standard fMRI aspect of analysis. As with our previous work[6], the validated PASAT will be employed to transiently fatigue the subject. The task is a measure of cognitive function; specifically auditory processing, calculation, working memory and attention. Participants will be asked to listen to a series of numbers ranging from 1 to 9. They are required to sum consecutive numbers (i.e. the first to the second, the second to the third etc.) and to record, via a button press, every occasion two consecutive numbers sum to the number 10. Concurrently, they will be asked to focus on a computer screen displaying three boxes containing random, rapidly changing numbers. This visual stimulus is intended to distract the participants from the auditory task and hence increase difficulty. They will be instructed not to process the visual numbers in any way.

Participants will then be asked to lie supine in the 3T Phillips Achieva X-series MRI scanner in Aberdeen or the equivalent scanner in Edinburgh or Glasgow. The multi-modal MR will consist of structural and functional sequences:

Structural: We will collect images to allow volumetric analysis. We will also acquire images to allow determination of white matter hyperintensity lesion load and measures of white matter structural integrity (e.g. DTI).

Functional Imaging: Images sensitive to BOLD contrast will be acquired during rest to investigate metrics such as intrinsic network connectivity as well as during the PASAT task (3x3minute periods interspersed by 30s rest periods).

In total these can will take approximately 45 minutes to conduct and will be repeated at approximately 6 months (when we predict to observe the greatest effect from the interventions).

Analysis

Following pre-processing of the MRI data the following analysis will be undertaken which will integrate data which will have been collected as part of the parent trial:

Longitudinal comparisons (paired t-tests as implemented by SPSS for ROI based variables and Freesurfer for voxel based variables) of structural and functional change indices in relation to subjects' change in fatigue will be performed. Putative confounders will be individually introduced as co-variates of interest. The individual analyses will focus upon those regions of interest previously identified by our studies, but since we recognise that these are not comprehensive we will also conduct agnostic (data-driven) whole brain analyses. All analyses will be adjusted for multiple testing.

Having identified and validated key neural areas, group differences in mediators of treatment effects on outcomes will be assessed via mediation analysis methods in order to tackle the secondary objective. These involve causal inference methods, such as structural equation modelling, to account for measurement error in the imaging data, and repeated measures to allow for the inclusion of all available data. Those resultant neural mediators which are common to both interventions and accessible to non-invasive neuromodulation will serve as our future therapeutic targets.

Finally we will be using whole brain statistical pattern recognition techniques on the neuroimaging data and mediation effects identified in answer to the previous objective. In the Pattern Recognition for Neuroimaging Toolbox (PRoNTo) brain scans are treated as spatial patterns and statistical

learning models are used to identify statistical properties of the data that can be used to discriminate between, or classify, experimental groups of subjects.

Sample size

We aim to recruit 120 participants (who will have been randomised to receive either usual care alone, CBA in addition to usual care or PEP in addition to usual care in the parent trial).

Data handling

MRI scan data will be stored in an anonymised format in the University of Aberdeen and Edinburgh imaging archive system on the university drive, with a back-up disc stored in a fireproof safe. The code for the images will be held on a separate computer relating the patient information to the participant ID. The participant ID will be used on the MRI images. Images may be stored on disc in anonymised format for research team discussions out-with the imaging department.

Safety assessments

The MRI scanner is very safe and does not expose participants to any harmful radiation. Given its reliance on a strong magnetic field, it is essential that certain metallic instruments/objects are not taken into the scan room.

This is avoided by:

1) All participants are clothed in 'theatre greens' so to avoid the danger of concealed metal objects within clothes.

2) Patients will be screened for absolute exclusion to MRI scanning.

In addition, all participants must undertake a strict and comprehensive checklist prior to scanning. This includes questions about heart valves, pacemakers and other potential metallic implants.

If a participant becomes distressed during the MRI scan, he/she will be able to access to a “panic button” which will immediately terminate the procedure.

Sub-study matrix

The study connects to the 1-year LIFT recruitment phase which will begin in August 2017 (month 0).

Study steps	-3–0 m	0–6 m	6–12 m	12–18 m	18–24 m	24–30 m
Governance approvals						
RA Subject recruitment						
MRI scan #1 (pre-intervention)						
MRI scan #2 (post-intervention)						
Data processing, analysis & report						

References

1. Hewlett S, Cockshott Z, Byron M, et al. Patients' perceptions of fatigue in rheumatoid arthritis: overwhelming, uncontrollable, ignored. *Arthritis and Rheumatism.* 2005;53(5):697-702.
2. Davies H, Brophy S, Dennis M, Cooksey R, Irvine E, Siebert S. Patient perspectives of managing fatigue in Ankylosing Spondylitis, and views on potential interventions: a qualitative study. *BMC Musculoskelet Disord.* 2013;14(1):163.
3. Patton MQ. *Qualitative Research & Evaluation Methods.* 3rd ed. Thousand Oaks, Calif. ; London: Sage; 2002.
4. Druce KL, Jones GT, Macfarlane GJ, Basu N. Determining pathways to improvements in fatigue in rheumatoid arthritis: results from the British Society for Rheumatology Biologics Register for Rheumatoid Arthritis. *Arthritis & Rheumatology.* 2015;67:2303-2310.
5. Druce KL, Jones GT, Macfarlane GJ, Basu N. The changes in pain which mediate Reductions in fatigue likely reflect central not peripheral mechanisms: Results from the BSRBR-RA. *Arthritis Care & Research.* 2016; 68(7):922-6.
6. Basu N, Murray AD, Jones GT, Reid DM, Macfarlane GJ, Waiter GD. Neural correlates of fatigue in granulomatosis with polyangiitis: a functional magnetic resonance imaging study. *Rheumatology.* 2014;53:2080-2087.
7. Basu N, Murray AD, Jones GT, Reid DM, Macfarlane GJ, Waiter GD. Fatigue-related brain white matter changes in granulomatosis with polyangiitis. *Rheumatology.* 2013; 52(8):1429-34.
