# Supplementary material for: Protocol for a multicentre randomised controlled parallel-group trial to compare the effectiveness of remotely delivered cognitive-behavioural and graded exercise interventions with usual care alone to lessen the impact of fatigue in inflammatory rheumatic diseases (LIFT)
Source: BMJ Open. 2019 Jan 30;9(1):e026793. doi: 10.1136/bmjopen-2018-026793 (PMC6359876; doi:10.1136/bmjopen-2018-026793)
Supplement: Author's manuscript [file bmjopen-2018-026793.draft_revisions.pdf]

BMJ Open is committed to open peer review. As part of this commitment we make the peer review history of every article we publish publicly available.

When an article is published we post the peer reviewers' comments and the authors' responses online. We also post the versions of the paper that were used during peer review. These are the versions that the peer review comments apply to.

The versions of the paper that follow are the versions that were submitted during the peer review process. They are not the versions of record or the final published versions. They should not be cited or distributed as the published version of this manuscript.

BMJ Open is an open access journal and the full, final, typeset and author-corrected version of record of the manuscript is available on our site with no access controls, subscription charges or pay-per-view fees (http://bmjopen.bmj.com).

If you have any questions on BMJ Open's open peer review process please email info.bmjopen@bmj.com

BMJ Open

Protocol for a multi-centre randomised controlled parallel-group trial to compare the effectiveness of remotely delivered cognitive behavioural and graded exercise interventions with usual care alone to lessen the impact of fatigue in inflammatory rheumatic diseases (LIFT)

Journal:	*BMJ Open*
Manuscript ID	bmjopen-2018-026793
Article Type:	Protocol
Date Submitted by the Author:	19-Sep-2018
Complete List of Authors:	Martin, Kathryn; University of Aberdeen, Epidemiology Group, Institute of Applied Health Sciences<br>Bachmair, Eva-Maria; University of Aberdeen, Epidemiology Group, Institute of Applied Health Sciences<br>Aucott, Lorna; University of Aberdeen , Centre of Healthcare and Randomised Trials (CHaRT), Health Services Research Unit<br>Dures, Emma; University of the West of England, Nursing and Midwifery<br>Emsley, Richard; King's College London, Department of Biostatistics and Health Informatics, Institute of Psychiatry, Psychology and Neuroscience<br>Gray, Stuart; University of Glasgow, Institute of Cardiovascular and Medical Sciences, BHF Glasgow Cardiovascular Research Centre<br>Hewlett, Sarah; University of the West of England,<br>Kumar, Vinod; Ninewells Hospital, NHS Tayside<br>Lovell, Karina; The University of Manchester, School of Health Sciences<br>Macfarlane, Gary; University of Aberdeen, Epidemiology Group, Institute of Applied Health Sciences<br>Maclennan, Graeme; University of Aberdeen, Centre of Healthcare and Randomised Trials (CHaRT), Health Services Research Unit<br>McNamee, Paul; University of Aberdeen<br>Norrie, John; Edinburgh Clinical Trials Unit, University of Edinburgh No. 9, Bioquarter<br>Paul, Lorna; Glasgow Caledonian University School of Health and Life Sciences,<br>Ralston, Stuart; University of Edinburgh, Rheumatology and Bone Disease<br>Siebert, Stefan; University of Glasgow, Institute of Infection, Immunity and Inflammation; NHS Greater Glasgow and Clyde,<br>Wearden, Alison; University of Manchester, School of Health Sciences<br>White, Peter; Queen Mary University of London,, Department of Psychological Medicine, Bartholomew's Hospital<br>Basu, Neil; University of Glasgow, Institute of Infection, Immunity and Inflammation
Keywords:	LIFT, Fatigue, Inflammatory rheumatic disease, randomised controlled trial, Cognitive behavioural, exercise

For peer review only - http://bmjopen.bmj.com/site/about/guidelines.xhtml

Protocol for a multi-centre randomised controlled parallel-group trial to compare the effectiveness of remotely delivered cognitive behavioural and graded exercise interventions with usual care alone to lessen the impact of fatigue in inflammatory rheumatic diseases (LIFT)

Kathryn R Martin[1,2*,**], Eva-Maria Bachmair[1,2*], Lorna Aucott[3], Emma Dures[4], Richard Emsley[5], Stuart R Gray[6], Sarah Hewlett[4], Vinod Kumar[7], Karina Lovell[8], Gary J Macfarlane[1,2], Graeme MacLennan[3], Paul McNamee[9], John Norrie[10], Lorna Paul[11], Stuart Ralston[12], Stefan Siebert[13], Alison Wearden[14], Peter D White[15], Neil Basu[13]

[*] joint first authors

[1] Epidemiology Group, University of Aberdeen, Health Science Building 1st Floor, Foresterhill, Aberdeen AB25 2ZD, UK; [2] Aberdeen Centre for Arthritis and Musculoskeletal Health, University of Aberdeen, Foresterhill, Aberdeen AB25 2ZD, UK; Email: e.bachmair@abdn.ac.uk, g.j.macfarlane@abdn.ac.uk, kathryn.martin@abdn.ac.uk;

[3] Centre of Healthcare and Randomised Trials (CHaRT), Health Service Research Unit, University of Aberdeen, Health Science Building 3rd Floor, Foresterhill, Aberdeen AB25 2ZD, UK; Email: g.maclennan@abdn.ac.uk, l.aucott@abdn.ac.uk

[4] Department of Nursing and Midwifery University of the West of England, Academic Rheumatology Unit, Bristol Royal Infirmary, Bristol BS2 8HW, UK; Email: Emma2.dures@uwe.ac.uk; Sarah.hewlett@uwe.ac.uk

[5] Department of Biostatistics and Health Informatics, Institute of Psychiatry, Psychology and Neuroscience, King's College London, De Crespigny Park,

For peer review only - http://bmjopen.bmj.com/site/about/guidelines.xhtml

Denmark Hill, London SE5 8AF, UK; Email: Richard.Emsley@kcl.ac.uk

[6] Institute of Cardiovascular and Medical Sciences, BHF Glasgow Cardiovascular Research Centre, University of Glasgow, Glasgow G12 8TA, UK; Email: stuart.gray@glasgow.ac.uk

[7] Ninewells Hospital, NHS Tayside, Dundee DD1 9SY, UK; Email: Kumar.vinod@nhs.net

[8] School of Health Sciences, University of Manchester, Jean McFarlane Building, University of Manchester, Oxford Road, Manchester M13 9PL, UK; Email: karina.lovell@manchester.ac.uk

[9] Health Economics Research Unit, University of Aberdeen, Polwarth Building, Foresterhill, Aberdeen AB25 2ZD, UK; Email: p.mcnamee@abdn.ac.uk

[10] Edinburgh Clinical Trials Unit , University of Edinburgh, Outpatients Building, Floor 2, Western General Hospital, Crewe Road South Edinburgh EH4 2XU, UK; Email: j.norrie@ed.ac.uk

[11] School of Health and Life Science, Glasgow Caledonian University, Cowcaddens Road, Glasgow G4 0BA, UK; Email: lorna.paul@gcu.ac.uk

[12] Rheumatology and Bone Disease, University of Edinburgh, Western General Hospital, Edinburgh EH4 2XU, UK; Email: stuart.ralston@ed.ac.uk

[13] Institute of Infection, Immunity and Inflammation, University of Glasgow, 120 University Place, Glasgow G12 8TA, UK; Email: stefan.siebert@glasgow.ac.uk; neil.basu@glasgow@ac.uk

[14] School of Health Sciences, University of Manchester, Coupland 1 Building, Oxford Road, Manchester, United Kingdom M13 9PL, UK; Email: alison.wearden@manchester.ac.uk

For peer review only - http://bmjopen.bmj.com/site/about/guidelines.xhtml

[15] Department of Psychological Medicine, Queen Mary University of London, Bartholomew's Hospital, William Harvey House, West Smithfield, London EC1A 7BE, UK; Email: peter.white6@qmul.ac.uk

**Corresponding author: Dr Kathryn R Martin, Epidemiology Group, University of Aberdeen, Health Science Building 1st Floor, Foresterhill, Aberdeen AB25 2ZD, United Kingdom; Email: kathryn.martin@abdn.ac.uk; Telephone: +44 (0) 1224 437117

Word count: 5800

For peer review only

ABSTRACT

Introduction: Fatigue remains pervasive, disabling and challenging to manage across all inflammatory rheumatic diseases (IRDs). Non-pharmacological interventions, specifically cognitive behavioural approaches and graded exercise programmes designed to support and increase exercise, are valuable treatments which help IRD patients manage their fatigue. Yet, health care systems have encountered substantial barriers to the implementation of these therapeutic options. LIFT is designed to give insights into the effectiveness of a remotely delivered standardised intervention for a range of IRD patients. It will also enable the exploration of putative moderating factors which may allow for the future triage of patients and to investigate the precise mediators of treatment effect in IRD-related fatigue.

Methods and Analysis: LIFT (Lessening the Impact of Fatigue in inflammatory rheumatic diseases: a randomised Trial) is a pragmatic, multi-centre, three-arm randomised, controlled trial, which will test whether adapted cognitive behavioural approach (CBA) and personalised exercise programme (PEP) interventions can individually reduce the impact and severity of fatigue. This will be conducted with up to 375 eligible patients diagnosed with IRD and interventions will be delivered by rheumatology health care professionals, using telephone or internet-based audio/video calls.

Ethics approval and dissemination: Ethical approval has been granted by Wales REC 7 (17/WA/0065). Results of this study will be disseminated through presentation at scientific conferences and in scientific journal. A lay summary of the results will be sent to participants.

Article Summary

For peer review only

Strengths and limitations of this study

- LIFT is designed to give insights into the effectiveness of a remotely delivered standardised intervention for a range of IRD patients.
- It will also enable the exploration of putative moderating factors which may allow for the future triage of patients and to investigate the precise mediators of treatment effect in IRD-related fatigue.
- Although participants and therapist are not blinded to treatment allocation, all research staff at study sites performing assessments are blinded.

Trial registration: ClinicalTrials.gov ID NCT03248518, registered on 14 August 2017

Keywords: LIFT, fatigue, inflammatory rheumatic disease, randomised controlled trial, cognitive behavioural, exercise, telephone, telehealth

For peer review only

INTRODUCTION

Despite major advances in the management of inflammatory rheumatic diseases (IRDs), patients remain burdened by their disease and cite fatigue as a principal problem. In rheumatoid arthritis (RA), for example, as many as 80% of patients report significant fatigue [1] and over 70% consider fatigue to be equal to pain in terms of burden [2]. Moreover, fatigue is a crucial determinant of impaired quality of life [3, 4] and a predictor of work disability [5, 6]. Indeed, over 75% of patients identify fatigue as the main barrier to remaining in employment [7]. Studies in other chronic IRDs, such as axial spondyloarthritis (AxSpA) and systemic lupus erythematosus (SLE), have reported similar fatigue prevalence of 66-85% [8, 9] and report the impact of fatigue on quality of life and employment to be equally pronounced [10-12]. In spite of these profound consequences, patients feel this symptom is clinically neglected [13, 14] and rheumatologists admit uncertainty regarding its management [15].

This situation reflects the poor availability of suitable interventions within traditional health care systems such as the NHS, but this is not to say that effective treatments do not exist. There is now considerable consensus across the health care community that non-pharmacological interventions, specifically cognitive behavioural approaches (CBA) and programmes designed to support increased exercise, are valuable treatments which help IRD patients manage the fatigue associated with their chronic diseases [16, 17].

Our current team has made key contributions to the evidence base, which supports the use of these treatments for fatigue in IRDs [18-20]. However, current health care systems pose to substantial barriers to the implementation of these therapeutic options as part of standard clinical care.

For peer review only

Firstly, existing studies – including our own – have only developed bespoke disease-specific models of care, which vary in content, structure and method of delivery. Inevitably, this necessitates the development of multiple particular skill sets and duplicated pathways for the care providers if they are to equitably serve their diverse patient populations – a time consuming, costly and inefficient undertaking.

Secondly, patients find it challenging to commit to regular face-to-face treatment sessions (a common underpinning of existing CBA and exercise interventions). This is often due to a combination of health complications and the time-constrained nature of modern life, particularly relevant to those patients still in employment.

Thirdly, individual patients report substantial variation in their preference and response to the distinct interventions of CBA and exercise [21, 22].

Rationale for Study

It has becoming increasingly clear that:

a) similarities exist across chronic IRDs regarding the nature and likely mechanisms which maintain fatigue – such as dysfunctional activity behaviours [23-25] and illness beliefs [14, 26, 27] ,- and so the application of standardised generic, rather than disease-specific, interventions may prove effective;

b) alternative, more flexible, methods of remote delivery such as telephone and internet-based audio/video calls can be just as effective as traditional face to face interventions [28, 29]; and

c) in the future, the identification of baseline patient preferences and characteristics which can predict differential treatment effects (moderators) will be vital to inform a personalised triage approach to care [22, 30].

For peer review only - http://bmjopen.bmj.com/site/about/guidelines.xhtml

Therefore, a pragmatic study is proposed, which will use CBA and personalised exercise programme (PEP) interventions, informed by previously developed interventions [31, 32] which will be generically applicable across IRD-fatigue populations. We will test whether these two key non-pharmacological interventions compared with usual care alone can individually reduce fatigue, when delivered by the rheumatology team, across a mixture of IRDs using telephone or internet-based audio/video calls. In doing so, this will enable us to explore potential moderating factors which may allow for the future triage of patients according to the most suitable intervention, but in addition investigate the precise mediators of the effect of treatment on IRD-related fatigue.

Objectives

Primarily, we seek to test the hypothesis that either standardised CBA or PEP interventions, in addition to usual care, is more effective than usual care alone to lessen the impact and severity of fatigue. We will also explore the underlying moderators (in order to inform future patient triage) and mediators (in order to optimise future interventions) of IRD-related fatigue.

For peer review only - http://bmjopen.bmj.com/site/about/guidelines.xhtml

METHODS AND ANALYSIS

Study description

LIFT is a multi-centre, three-arm randomised controlled trial testing usual care with additional adapted CBA or PEP interventions versus usual care alone. Eligible participants will be identified from approximately 3600 patients with IRD attending major secondary care rheumatology services in the UK (Figure 1). Recruitment for the study began in August 2017. We anticipate recruiting 375 participants previously diagnosed with IRD.

The timeline for assessment and delivery of interventions is summarised in Figure 2. We anticipate that active CBA and PEP interventions will start between 2 and 8 weeks post-randomisation, with an average delay of 4 weeks after randomisation. Follow-up data will be collected from all participants at 10 weeks, 28 weeks and 56 weeks after randomisation adjusted for the average delay of 4 weeks, therefore all participants will remain part of the study for 13 months.

Participant Recruitment and Eligibility

Enrolment is by invitation only and all potential participants will undergo a two-step screening process consisting of a pre-study invite and an assessment of their hospital medical notes. Patients with rheumatologist diagnosed IRDs (e.g. RA, SLE and AxSpA, psoriatic arthritis, vasculitis or Sjogren's Syndrome) will receive a mailed pre-study invite consisting of a cover letter (see Supplementary material, Additional File 1 for details on Study within a Trial), study information and questions to explore interest and eligibility.

For peer review only - http://bmjopen.bmj.com/site/about/guidelines.xhtml

After return of the required pre-study screening questions, the research personnel at each site will assess their medical notes to determine eligibility based on the inclusion and exclusion criteria (Table 1).

Table 1 Inclusion and exclusion criteria

In order to be considered eligible for participation in the study they must:	
Criterion	Characteristics of eligible participants
1.	Be male or female aged ≥ 18 years at the time of consent
2.	Have been diagnosed with an IRD such as RA, SLE or AxSpA by a consultant rheumatologist
3.	Report fatigue to be a persistent problem as evidenced by answering both questions:<br>1. Have you had problems with fatigue for more than three months? (Yes)<br>2. Please circle the number that shows your average level of fatigue during the past 7 days. (≥ 6 based on a numerical rating scale of 0 (no fatigue) to 10 (totally exhausted))
4.	Have access to a telephone landline or mobile telephone and/or internet based audio/video calls
5.	Give permission for researchers to access their hospital medical notes
6.	Have stable disease as evidenced by no change in immunomodulatory therapy within the last three months based

For peer review only

	on hospital medical records
7.	Currently be under the care of a secondary care physician
Participants will be excluded if:	
Criterion	Characteristics of ineligible participants
1.	there are significant abnormalities in thyroid function (TSH levels) in the most recent blood test done within the last three months
2.	there is evidence of severe anaemia (haemoglobin levels) in the most recent blood test done within the last three months
3.	there is evidence of severe renal dysfunction (eGFR) in the most recent blood test done within the last three months
4.	they have a medical condition which would make the proposed interventions unsuitable, e.g. significant heart disease
5.	they are pregnant
6.	they are unable to understand English sufficiently to take part in the intervention
7.	they are unable to provide written informed consent
8.	they are not willing to be randomised
9.	they are currently participating in an interventional clinical trial

Abbreviations: AxSpA, Axial Spondyloarthritis; eGFR, estimated glomerular filtration rate; RA, rheumatoid arthritis; SLE, Systemic Lupus Erythematosus, TSH, Thyroid Stimulating Hormone

A trained rheumatology research nurse will review and confirm eligibility. In addition, the local rheumatology consultant will have the opportunity to withdraw or exclude

For peer review only - http://bmjopen.bmj.com/site/about/guidelines.xhtml

participants. If a participant has been identified as potentially eligible s/he will be invited to an appointment for a baseline assessment visit at the local study site.

Consenting participants

Patients will make a final decision to participate when they attend the local study site for the baseline assessment visit. No study specific procedures will take place before written consent has been obtained. At the baseline assessment visit, a designated member of the local research team will confirm eligibility (including re-confirmation of fatigue state). It will also be determined whether the participant has ever met the relevant classification criteria for RA (mandatory, 2010 ACR/EULAR [33] or 1987 ACR [34]), or other IRDs (non-mandatory; SLE 1997 ACR SLE criteria [35], ASAS criteria for AxSpA [36]).

Randomisation

Participants will be allocated to receive either of the two treatments or usual care (1:1:1 ratio) using a computer generated sequence. Randomisation will be minimised by diagnosis (RA, SLE, or AxSpA or other IRD) and the presence/absence of depressive symptoms (Hospital Anxiety & Depression Scale (HADS) [37] subscale score >10) and will include a random element set at 20%.

Blinding

Full blinding will not be possible due to the need for participants to engage in specific behavioural change interventions. However, to reduce detection bias, we will aim to “blind” research personnel undertaking outcome assessments to participants’ treatment allocation. To facilitate blinding we will remind participants to refrain from

For peer review only - http://bmjopen.bmj.com/site/about/guidelines.xhtml

discussing (and subsequently revealing) their treatment allocation at follow-up assessments with research personnel. Finally, all data will be analysed blind to allocation.

Interventions and Treatment protocol

Usual Care

All participants will receive usual care and will receive an Arthritis Research UK education booklet for self-management of fatigue [38] by post. It represents usual care in almost all UK rheumatology centres and is freely available. The booklet covers the major relevant topics (including fatigue validation, energy management, priorities, sleep, stress, and assertiveness) underpinned by goal-setting and self-monitoring of activity. It encourages, at several key points that the patient asks their rheumatology team for support to work through the booklet. We will not restrict what usual care may involve, but will monitor the care received for all participants as part of our health economics analysis.

Participants randomised to the active treatments will also receive either CBA or PEP. The CBA and PEP treatments are adapted from previous fatigue specific cognitive behavioural [31, 32] and exercise interventions [31] to ensure that they are suitable for a remote delivery via telephone or internet-based audio/video call and applicable to the broad spectrum of IRD.

Cognitive Behavioural Approach (CBA)

For peer review only - http://bmjopen.bmj.com/site/about/guidelines.xhtml

CBA is a structured psychological intervention, which explicitly aims to replace unhelpful beliefs and behaviours with more adaptive ones. In this study, the CBA will target a number of unhelpful behavioural patterns such as 'activity avoidance' and 'all or nothing'. These can lead to negative mood states, which exacerbates fatigue even further. Following a brief assessment of individual patient beliefs and behaviours surrounding fatigue, the aim of the treatment is to change unhelpful beliefs and behavioural factors through the application of patient-centred strategies and behavioural activities, which are supported by written materials and regular consultations with rheumatology health care professionals. The participants will receive additional leaflets and diaries to assist them with making changes to manage fatigue.

Personalised Exercise Programme (PEP)

PEP is based on the premise that chronic fatigue relates to exercise intolerance, supported by unhelpful illness beliefs (such as fear avoidance) and deconditioning, with a consequent increase of effort (perceived or otherwise). PEP aims to disrupt this vicious cycle by a graded exposure behaviour therapy, which is symptom contingent, to gradually optimise patients' levels of exercise with a view to modifying their altered perception of effort, improve their tolerance of exercise, fitness and function, reverse the deconditioning and ultimately reduce the severity and impact of fatigue. Participants will receive an individually tailored graded exercise programme, delivered according to their physical capacity and gradually increased in duration and then intensity. The participants will receive additional information and diaries to assist with the intervention. The intervention will use pedometers and/or heart rate monitors

For peer review only - http://bmjopen.bmj.com/site/about/guidelines.xhtml

to enhance motivation. Overall, the times and duration of exercise will be recorded in exercise diaries during the intervention.

Both CBA and PEP interventions will be delivered by health care professionals. PEP may be delivered by a rheumatology specialist physiotherapist and CBA may be delivered by a rheumatology nurse or an equally qualified and trained allied health professional (e.g., occupational therapist) who are members of local NHS staff. Participants will be offered seven one-to-one telephone or internet-based audio/video call (based on patient preference) sessions (up to 45 minutes) of CBA or PEP interventions over a period of 14 weeks with a trained therapist (Figure 2). The first session of PEP, however, will be delivered face-to-face. A booster session will be conducted at 22 weeks after the start of therapy by the relevant therapist.

Training of therapists before the study

Separate CBA and PEP training will be provided for the NHS staff delivering the interventions. This will comprise an intensive 2-day group course delivered by experienced designated investigators supplemented with the therapist manuals. The course will use a range of methods including skills practice with specific feedback using fictitious but typical fatigued cases.

Supervision and support of therapists during the study

Supervision will be provided by designated investigators on a fortnightly basis, or as required, to the therapists either face to face or by telephone depending on feasibility and preference. In addition, support will be available in cases where a rheumatology health care professional requires assistance with respect to a particular participant. In

For peer review only - http://bmjopen.bmj.com/site/about/guidelines.xhtml

addition to the option to contact the supervisor directly, we will have a notification system incorporated in the database.

Treatment fidelity

Some of the intervention sessions will be recorded and used in supervision to provide feedback to rheumatology health care professionals and to ensure treatment fidelity. We aim to take a 5% sample of intervention sessions for those in CBA and PEP from participants who agree to be recorded which is based on a random sample generated from an algorithm that takes into account session number, therapist, site location, patient gender. This is equivalent to approximately 89 recordings per intervention and will be subject to treatment adherence (no sessions completed) and participant permission to record sessions.

Outcome measures

All outcome and mediator measures will be assessed at randomisation (baseline) and then, on average, 10 weeks, 28 weeks and 56 weeks thereafter (by questionnaire unless stated) in all participants (Figure 3). The outcome measures at each time point and their source (i.e. questionnaire, medical record, blood sample, diary) are summarised in a study matrix (Supplementary material Table 1).

Primary outcome measures

The primary outcome, fatigue, is measured with the Chalder Fatigue Scale (CF) [39] which assesses the physical and mental symptoms of fatigue using Likert scoring, and the Fatigue Severity Scale (FSS) [40] assessing the impact of fatigue at 56 weeks post randomisation. If the effect of the intervention is positive on the CF, then

For peer review only - http://bmjopen.bmj.com/site/about/guidelines.xhtml

the FSS outcome will be formally analysed. Should the intervention have no effect on the CF, then an explorative analysis of the FSS data will be performed.

Secondary outcome measures

Secondary outcomes are:

Fatigue: Bristol Rheumatoid Arthritis Fatigue Multi-Dimensional Questionnaire [41] (BRAF-MDQ) assessing the physical, living, cognition and emotional aspects of fatigue

Quality of life & health utility index: Short Form-12 [42] assessing functional health and wellbeing from the patient's perspective

Pain: Pain numerical rating scale [43] assessing pain intensity

Anxiety and depression: Hospital anxiety and depression scale (HADS) [37]

Sleep: Sleep problem scale [44]

Impact on work: Work Productivity and Activity Impairment Questionnaire: Specific Health Problem (WPAI:SHP) [45]

Impact on activities: Valued Life Activities Scale (short form S-VLA) [46]

Global outcome: Change of global health

Mediator/moderator measures

While many secondary outcome measures may also function as mediators, or their baseline values as moderators, more detailed cognitive, behavioural, clinical and physical data will be collected in order to fully characterise these factors.

Cognitions and behaviours: Brief Illness Perception Questionnaire [47], Behavioural Response to Illness Questionnaire [48].

For peer review only

Clinical: Presence of fibromyalgia [49], serological status, erosive status, disease duration, previous and current pharmacological therapies, disease activity: self-reported using a Numeric rating scale, DAS28 for RA (mandatory), other disease specific activity measures (non-mandatory), inflammation (CRP/ESR), presence of other co-morbidities (Charlson Comorbidity Index) [50].

Physical: Physical activity and sedentary behaviour profiles will be measured by an activity monitor (*activ*PAL, Paltechnologies Ltd, Glasgow). The *activ*PAL will be fitted to the participant at each assessment session and participants instructed to remove the device and post it back to the research team after seven days in the stamped addressed envelope provided to them.

Quantifying aerobic fitness: A step test, which involves participants wearing a heart rate monitor and stepping onto a 10 inch high box for three minutes at different stepping rates to assess aerobic fitness. Participants stop the test if their heart reaches 65% of predicted maximal heart rate (220-age or 190-age if participant is prescribed beta-blocker) at the end of any stage. One minute of rest is given between stages and maximal oxygen uptake is estimated from heart rate recordings according to established equations [51]. In addition, values of Borg Rating of Perceived Exertion (RPE) are collected.

Neuroimaging data: Participants will have an option to undertake a multi-modal magnetic resonance imaging (MRI) brain scan (see Supplementary material Additional file 2)

Furthermore, participants will be given the option to provide additional blood samples which will be stored in a designated freezer at the University of Aberdeen. These will be a maximum of 3 tubes per visit of 1x PAXgene DNA (visit 1 only), 1x PAXgene

For peer review only - http://bmjopen.bmj.com/site/about/guidelines.xhtml

RNA (visit 1, 2, 3), 1x serum (visit 1, 2, 3). Additional consent for their use in future unspecified studies will be obtained.

Quantitative process evaluation

Patient preference: Participants will be given a short synopsis of all three treatments, usual care, CBA and PEP interventions as treatment for IRD-related fatigue at baseline. They will then be asked about which treatment they would choose if they had a choice, as well as about their strength of preference.

Patient adherence: Adherence to the interventions will be monitored via attendance records kept by the therapists. In addition, participants receiving the CBA or PEP interventions will be contacted by a member of Trial Office in Aberdeen at time of session 4 (approximately week 8) and session 8 (approximately week 26) for a telephone interview. They will be asked to indicate on a scale from 0 (not at all) to 10 (completely), if they think that this treatment is the right approach and their willingness to engage and adhere to the intervention. At the same time, the therapists will be asked during supervision to what extent they think that the participant has engaged with treatment and adhered to the agreed actions and plans.

Intervention acceptance: Acceptance by participants will be evaluated in all three treatment arms using the Client Satisfaction Questionnaire [52] at 28 weeks.

Qualitative evaluation

A subgroup of participants will be invited to take part in a nested qualitative evaluation study. We will conduct qualitative evaluations of both participants who received CBA or PEP after they completed the 56 week follow-up and all therapists who will deliver the interventions. To ensure integrity, participants and therapists will

For peer review only - http://bmjopen.bmj.com/site/about/guidelines.xhtml

be invited to take part once they have left or completed the study. Data will be collected through semi-structured interviews conducted by telephone or by internet-based audio/video calls. In addition to the practical considerations of offering options, this approach acknowledges that both participants and therapists will have been involved in a remotely delivered intervention. All interviews will be audio recorded, anonymised during transcription, and checked for accuracy.

Participant experiences of the interventions will be analysed using a framework analysis [53] to assess the content, mode of delivery, acceptability, barriers and facilitators, helpfulness and subsequent impact of the interventions on their daily lives. We will use a maximum variation sampling strategy [54] to include participants with a range of IRDs, gender, age, disease duration and primary outcomes in the randomised controlled trial. To achieve this, a minimum of 40 interviews is required. All selected participants will receive additional information after they have completed the last assessment visit (week 56) and provide separate written informed consent for the qualitative component.

All therapists will be invited to take part in a telephone interview to explore experiences of intervention training and delivery, including challenges and benefits of learning and using new skills, and barriers and facilitators to supporting patients remotely using a structured manual. This evaluation will be analysed using inductive thematic analysis [55]. All therapists will receive additional information after they have either left the study or after the intervention phase and provide separate written informed consent for the qualitative component.

For peer review only

For peer review only - http://bmjopen.bmj.com/site/about/guidelines.xhtml

Patient safety

There are unlikely to be major safety issues with our proposed non-pharmacological interventions. However, if the rheumatology healthcare professional delivering CBA and PEP or any study personnel have any safety concerns, we will follow a specific Standard Operating Procedure for adverse events in non-CTIMP studies.

Laboratory and sample analysis

This multi-centre study involves a number of standard blood investigations, i.e. TSH, haemoglobin, serum creatinine, CRP and ESR, which are routinely processed by local NHS laboratories. Blood samples for CRP and ESR analysis and sample storage for future ethically approved research will be taken at each assessment as specified in the study matrix by trained personnel only following established procedures. Blood samples for TSH, haemoglobin and serum creatinine analysis will only be taken at baseline if they are required to confirm eligibility. Sample coding, preparation, storage, analysis and transfer of results and optional blood samples for long-term storage at the trial centre will be performed according to the analytical protocol based on national laboratory guidelines. The exact logistic and procedure will be agreed with each site before the start of the study and every effort will be made to standardise the workflow across sites to reduce bias. Additional optional blood samples will be stored for future ethically approved research. All study blood results (abnormal or otherwise) will also be directed towards the local PI who can determine locally how these will be handled.

For peer review only - http://bmjopen.bmj.com/site/about/guidelines.xhtml

Withdrawal Procedures

Participants will have the option to withdraw at any time during the study period of 13 months. The participant needs to request this formally and a withdrawal document will be completed and signed by the designated research staff at the local sites. They have the option to either withdraw from the study completely or from parts of it (pre-study invite, treatment or follow-up). If participants withdraw from the study completely or from follow-up assessments, they will not receive further invitations but we will use the data collected prior to the withdrawal (depending on permission). Those withdrawing from the treatment only will continue to be sent invitations to attend and complete follow-up assessment visits, unless they request to withdraw completely later on. Failure of any participant to complete a follow-up at any particular time-point will not be counted as a withdrawal unless the participant formally requests to withdraw. In addition, a participant can also be withdrawn by others, for example the local PI or primary consultant, should the need arise at any stage throughout the study period. This also includes loss of capacity for ongoing consent.

Statistical Issues

Sample size

Our planned primary intention to treat analyses will compare PEP + usual care versus usual care alone, and CBA + usual care versus usual care alone separately. We base our calculations on a standardised effect size of 0.50 (considered credible in other pragmatic effectiveness studies). This would equate to being powered to detect a minimal important clinical difference of 2 units in the CF Scale, assuming a common standard deviation across the randomised groups of 4 units. Assuming an

For peer review only

overall significance level of 5% (by calculating the two pre-specified randomised groups comparisons, PEP + usual care vs. usual care alone and CBA + usual care vs usual care alone, at 2.5%, to maintain an overall level of not more than 5%) and a power of 90%, we require 100 evaluable participants in each of the three groups. The data of participants are evaluable when outcomes at the 56 weeks follow-up are available. Based on our own previous studies, we estimate a dropout rate of 20% and therefore we anticipate recruiting 375 participants randomly allocating 125 into each treatment group.

Statistical analysis

All statistical analyses will be governed by a comprehensive Statistical Analysis Plan, which will be authored by the trial statistician and approved by the Trial Steering Committee and the Data Monitoring Committee before the main study outcome data are examined. All analyses will be carried out using standard statistical software. In accordance with CONSORT guidelines [56], we will report all participant flow. Descriptive statistics of recruitment, drop-out and completeness of interventions will be provided.

Effectiveness Analysis: The main effectiveness analysis will be via intention-to-treat including all participants, with no planned interim analysis for early termination for either overwhelming evidence of effectiveness or abandoning for futility. Baseline characteristics will be presented by randomised group without formal statistical tests. We will test the primary hypothesis for between-group change in the primary outcome for each of the two pre-specified comparisons (CBA + usual care vs usual care alone and PEP + usual care vs usual care alone) using repeated measures

For peer review only

mixed model, with subject as a random effect, and a suitably specified covariance structure (e.g. Autoregressive [1] (AR[1]), rheumatology healthcare professional as a random effect (to adjust for any clustering by rheumatology healthcare professional), with baseline outcome measure, and any other strongly predictive baseline measures, including the minimisation factors of presence/absence of depression and centre. Treatment and its interaction with time will be fitted as fixed effects, and we will apply standard regression diagnostics. The analysis will use statistical techniques for handling missing outcome data using multiple imputation under a missing at random (MAR) assumption. The secondary outcomes will be analysed using an analogous method. The main estimate of treatment effect will focus on the 56 weeks after baseline.

Mediation analysis: We will use modern causal inference methods to investigate the set of mediator measures. If the effectiveness analysis shows significance between group differences in the mediators, then we will use parametric regression models to test for effect of mediator on outcome, and the residual direct effect of treatment on outcome. Since all the measures are continuous, the indirect effects will be calculated by multiplying relevant pathways and bootstrapping will be used to produce valid standard errors for the indirect effects. All analyses will adjust for baseline measures of the mediators, outcome and putative measured confounders. Mediation analyses are potentially biased by measurement error in mediators and hidden confounding between mediators and outcomes; we will build on our previous methodological and applied work in this context to include repeated measurement of mediators and outcomes to account for classical measurement error and baseline confounding.

For peer review only

Moderation analysis: We will examine differential treatment effects using the set of moderator measures by extending the intention to treat analysis models to include an interaction term between intervention and each of the moderators separately. We will use bias correction/cross-validation methods to identify robust evidence for individual moderation and for a moderation index, both on the overall effect and also along the steps of the mediation pathway.

Every effort will be made to ensure data collection is complete. However, we will use statistical techniques for handling missing outcome data. Multiple imputation under a MAR assumption will be used in the first instance, with additional sensitivity analysis if the MAR assumption is not satisfied.

Health Economics evaluation

An economic evaluation will be conducted from both a health care system and societal perspective. Participants will be asked to record in a diary all types and duration of hospital admissions, frequency of visits to hospital for outpatient attendances, and other visits to or from relevant health professionals (e.g. general practitioners, nurse practitioners, physiotherapists) and specify whether the main reason for the visit was fatigue. The participant will be asked to keep the diaries between the baseline and third assessment visit (approximately 28 weeks). Furthermore they will be asked to keep diaries for two weeks after the third assessment visit and two weeks before they return for the last assessment visit. National sources of unit cost data will be applied to value resource use (HRG Reference Costs, Unit Costs of Health and Social Care). The costs associated with

For peer review only - http://bmjopen.bmj.com/site/about/guidelines.xhtml

delivery of the interventions will be estimated using records kept by therapists of the number and duration of calls per patient.

Patients will be asked to report any contacts with private practitioners, and the costs of over the counter medication/complementary therapies purchased. This also includes additional expenses related to their condition or fatigue as well as if there was an impact on paid and unpaid work.

Health-related quality of life data will be collected using the SF-12 and these data will be converted to quality of life weights using published tariffs. These data will then be used to calculate Quality Adjusted Life Years (QALYs). As the intervention may affect general well-being as well as reduce fatigue, we will also collect values for changes in well-being data using the ICECAP instrument [57] and changes in life satisfaction [58].

Study management and conduct

The study will be co-ordinated by a Trial Management Group, consisting of the grant holder (CI), additional members of the research group, a study co-ordinator and a representative from CHaRT.

A Trial Steering Committee (TSC) has been established. The TSC comprises an independent chair who has expertise in trials and other members, both independent and study investigators, with background relevant to IRD or type of interventions including a lay representative, who has lived experience of IRD related fatigue, and a clinician working with people with IRD.

An independent Data Monitoring Committee has been established to oversee study progress. The Data Monitoring Committee comprises of a chair who has expertise in

For peer review only - http://bmjopen.bmj.com/site/about/guidelines.xhtml

trials, a biostatistician and two other independent members with a rheumatology background.

End of Study

The end of study is defined as last data collection of either the qualitative evaluation study phase or during the last follow-up visit at 56 weeks from the last participant after CBA/PEP intervention or usual care start date – whichever comes last.

Data management, protection, storage and archiving of study documents

All Investigators and study staff involved with this study will comply with the requirements of the Data Protection Act 2018 with regard to the collection, storage, processing and disclosure of personal information and will uphold the Act's core principles. The investigators and study staff will also adhere, if appropriate, to the current version of the NHS Scotland Code of Practice on Protecting Patient Confidentiality. Access to collated participant data will be restricted to the CI and appropriate study staff as needed. All laboratory specimens, evaluation forms, reports, and other records will be identified using unique participant ID numbers to maintain participant confidentiality. All records will be kept in a secure storage area with limited access to study staff only. Personal data, including postal address, phone numbers (i.e. landline and mobile), email addresses, and anonymised data files for study outcomes are stored in locked filing cabinets (hard copy) and in a bespoke database provided and maintained by CHaRT as well as secured shared drives with access via password controlled computers (university and NHS networks) by study staff only (electronic data).

For peer review only - http://bmjopen.bmj.com/site/about/guidelines.xhtml

All study documentation will be kept for a minimum of 5 years from the protocol defined end of study point in the University of Aberdeen archive. When the minimum retention period has elapsed, study documentation will not be destroyed without permission from the sponsor.

DISCUSSION

The LIFT trial is a pragmatic multi-centre, three-arm randomised controlled trial. It has been designed to test the effectiveness and cost-effectiveness of remotely delivering two non-pharmacological interventions, CBA and PEP, over a period of 22 weeks so as to answer whether treatment can move beyond face-to-face disease-specific interventions for IRD-related fatigue. In order to achieve the study aims the CBA and PEP interventions were informed by previous evidence-based cognitive behavioural and exercise interventions [31, 32] for fatigue to ensure suitability across IRD conditions, as well as to ensure that both interventions could be delivered via telephone or internet-based audio/video calls by a range of trained rheumatology health care professionals. To reduce selection bias, all patients with a relevant diagnosis, managed at each recruiting site who report fatigue as a persistent problem will be considered, either through database screening or manual screening by research nurses, and invited to take part in the trial. Participants will be asked to provide information about fatigue, our primary outcome, at baseline and weeks 10, 28, and 56 using the Chalder Fatigue Scale and Fatigue Severity Scale to measure severity and impact at critical times throughout the trial. In addition, detailed information on cognitions and behaviours (i.e. illness perception), clinical features (i.e. disease activity, presence of fibromyalgia), as well as physical activity and fitness levels will allow for the identification of potential moderators and mediators of

For peer review only - http://bmjopen.bmj.com/site/about/guidelines.xhtml

the treatment effect in this trial. Although complete blinding is not possible due to provision of an active behavioural change intervention, we aim to reduce detection bias by asking participants to refrain from revealing their treatment allocation to the research nurses undertaking their outcome assessments.

Fatigue remains a significant patient-identified priority, as well as a significant challenge to manage clinically. To provide the necessary evidence to bring about change in health services consistently offered for IRD-related fatigue across the spectrum of IRD conditions, both a quantitative and qualitative evaluation will also be undertaken within the trial. Trial findings will be used inform future triage of patients into the most suitable intervention, as well as provide evidence of which mediating factors play a role in treatment effect among patients with IRD-related fatigue.

Supplementary material

TABLE 1 Study Matrix

ADDITIONAL FILE 1 SWAT 24

ADDITIONAL FILE 2 MRI SUB-STUDY

Abbreviations

AxSpA, Axial Spondyloarthritis; CBA, Cognitive Behavioural Approach; CF, Chalder Fatigue Scale; CHaRT, Centre of Healthcare Randomised Trials; CI, Chief Investigator; CRP, C-reactive protein; eGFR, Estimated glomerular filtration rate; ESR, Erythrocyte Sedimentation Rate; FSS, Fatigue Severity Scale; ICECAP, Investigating Choice Experiments for the preference of older people CAPability measures for adults; ICH, International Council for Harmonisation; IRD, Inflammatory Rheumatic Disease; LIFT, Lessening the Impact of Fatigue in inflammatory

For peer review only - http://bmjopen.bmj.com/site/about/guidelines.xhtml

rheumatic diseases: a randomised controlled Trial; MAR, missing at random; NHS, National Health Service; PEP, Personalised Exercise Programme; RA, Rheumatoid Arthritis; REC, Research Ethics Committee; SLE, Systemic Lupus Erythematosus; TSH, thyroid stimulating hormone;

Acknowledgements

The authors would like to thank representatives of the National Rheumatoid Arthritis Society, the National Ankylosing Spondylitis Society and Lupus UK for their contribution to the study design before the grant application. The authors would also like to thank all members of our Patient Advisory Groups in Aberdeen and Bristol for their review of study material.

Funding and sponsorship

This work was supported by Arthritis Research UK grant number 21175. The study is sponsored and led by the University of Aberdeen (King's College, Aberdeen, AB24 3FX). Arthritis Research UK (funder) and Sponsor had no input into writing the manuscript.

Availability of data and materials

Ownership of the data arising from this study resides with the University of Aberdeen. A clinical study report will be prepared in accordance with ICH authorship guidelines which will be used for publication and presentation at scientific meetings. Investigators have the right to publish orally or in writing the results of the study. There are no plans to place study outcome data in a repository at the current time.

For peer review only - http://bmjopen.bmj.com/site/about/guidelines.xhtml

Author Contributions

NB lead the conception and design of the study with all other authors providing substantial contributions. JN and GMcL planned the statistical analysis. RE planned the mediation analysis. PM planned the health economics analysis. ED planned the qualitative evaluation analysis. EMB, KRM and NB wrote the first draft of this protocol, all other authors critically revised the manuscript, and gave final approval for publication.

Competing interests

The authors declare no other competing interests.

Consent for publication

Not applicable

Ethics approval and dissemination

The study will be conducted in accordance with the principles of good clinical practice.

The Research Ethics Committee Wales REC 7 (17/WA/0065) gave approval to the study on 20.03.2017 (protocol version 2) and approved the substantial amendment 01 on 06.07.2017 (protocol version 3), substantial amendment 02 on 05.09.2017 (protocol version 4) and substantial amendment 03 on 01.12.2017 (protocol version 5) and substantial amendment 04 on 08.03.2018, protocol version 6).Extension of recruitment for 6 months was approved and implemented as minor amendment 09 on 17.09.2018 (protocol version 7, current). Management approval from all NHS health

For peer review only

boards was obtained as required by the ethics committee before the start of the study.

Consent will be sought for recording of interventions sessions for quality assurance and treatment fidelity. Written consent will be obtained to record interviews and to include anonymised statements in publication of the qualitative evaluation outcomes.

Trial status

The LIFT study began recruitment in August 2017 and will be ongoing until March 2019 (anticipated). It is expected that data collection will be completed by April 2020.

REFERENCES

1 Repping-Wuts H, Van Riel P, Van Achterberg T. Fatigue in patients with rheumatoid arthritis: What is known and what is needed, *Rheumatology (UK)* 2009;48:207-9.

2 van Hoogmoed D, Fransen J, Bleijenberg G, et al. Physical and psychosocial correlates of severe fatigue in rheumatoid arthritis, *Rheumatology (UK)* 2010;49:1294-302.

3 Suurmeijer TPBM, Waltz M, Moum T, et al. Quality of life profiles in the first years of rheumatoid arthritis: Results from the EURIDISS longitudinal study, *Arthritis Care Res* 2001;45:111-21.

4 Rupp I, Boshuizen HC, Jacobi CE, et al. Impact of fatigue on health-related quality of life in rheumatoid arthritis, *Arthritis Care Res* 2004;51:578-85.

5 Gignac MAM, Sutton D, Badley EM. Reexamining the arthritis-employment interface: Perceptions of arthritis-work spillover among employed adults, *Arthritis Care Res* 2006;55:233-40.

6 De Croon EM, Sluiter JK, Nijssen TF, et al. Work ability of Dutch employees with rheumatoid arthritis, *Scand J Rheumatol* 2005;34:277-83.

7 National Rheumatoid Arthritis Society. Employment and Rheumatoid Arthritis in Scotland. A national picture (RA and work). 2014.

For peer review only

8 Aissaoui N, Rostom S, Hakkou J, et al. Fatigue in patients with ankylosing spondylitis: Prevalence and relationships with disease-specific variables, psychological status, and sleep disturbance, *Rheumatol Int* 2012;32:2117-24.

9 Zonana-Nacach A, Roseman JM, McGwin Jr. G, et al. Systemic lupus erythematosus in three ethnic groups. VI: Factors associated with fatigue within 5 years of criteria diagnosis, *Lupus* 2000;9:101-9.

10 Wang B, Gladman DD, Urowitz MB. Fatigue in lupus is not correlated with disease activity, *J Rheumatol* 1998;25:892-5.

11 Turan Y, Duruöz MT, Bal S, et al. Assessment of fatigue in patients with ankylosing spondylitis, *Rheumatol Int* 2007;27:847-52.

12 Mau W, Listing J, Huscher D, et al. Employment across chronic inflammatory rheumatic diseases and comparison with the general population, *J Rheumatol* 2005;32:721-8.

13 Hewlett S, Cockshott Z, Byron M, et al. Patients' perceptions of fatigue in rheumatoid arthritis: Overwhelming, uncontrollable, ignored, *Arthritis Care Res* 2005;53:697-702.

14 Davies H, Brophy S, Dennis M, et al. Patient perspectives of managing fatigue in Ankylosing Spondylitis, and views on potential interventions: A qualitative study, *BMC Musculoskelet Disord* 2013;14.

15 Repping-Wuts H, van Riel P, van Achterberg T. Rheumatologists' knowledge, attitude and current management of fatigue in patients with rheumatoid arthritis (RA), *Clin Rheumatol* 2008;27:1549-55.

16 National Institute for Health and Care Excellence (NICE). Rheumatoid arthritis: the management of rheumatoid arthritis in adults, *Clinical Guideline* 2009.

17 Arthritis and Musculoskeletal Alliance (ARMA). Standards of Care for people with Connective Tissue Diseases. 2007.

18 Tench CM, McCarthy J, McCurdie I, et al. Fatigue in systemic lupus erythematosus: A randomized controlled trial of exercise, *Rheumatology (UK)* 2003;42:1050-4.

19 Cramp F, Hewlett S, Almeida C, et al. Non-pharmacological interventions for fatigue in rheumatoid arthritis. *Cochrane Database Syst Rev* 2013;8.

20 Hewlett S, Ambler N, Almeida C, et al. Self-management of fatigue in rheumatoid arthritis: A randomised controlled trial of group cognitive-behavioural therapy, *Ann Rheum Dis* 2011;70:1060-7.

21 Jones E. Studying the influence of patient treatment preference on outcome in a clinical trial. 2012.

For peer review only

For peer review only - http://bmjopen.bmj.com/site/about/guidelines.xhtml

22 Johnson RE, Jones GT, Wiles NJ, et al. Active exercise, education, and cognitive behavioral therapy for persistent disabling low back pain: A randomized controlled trial, *Spine* 2007;32:1578-85.

23 Tench C, Bentley D, Vleck V, et al. Aerobic fitness, fatigue, and physical disability in systemic lupus erythematosus, *J Rheumatol* 2002;29:474-81.

24 Rongen-Van Dartel SAA, Repping-Wuts H, Van Hoogmoed D, et al. Relationship between objectively assessed physical activity and fatigue in patients with rheumatoid arthritis: Inverse correlation of activity and fatigue, *Arthritis Care Res* 2014;66:852-60.

25 Brophy S, Cooksey R, Davies H, et al. The effect of physical activity and motivation on function in ankylosing spondylitis: A cohort study, *Semin Arthritis Rheum* 2013;42:619-26.

26 Treharne GJ, Lyons AC, Hale ED, et al. Predictors of fatigue over 1 year among people with rheumatoid arthritis, *Psychol Health Med* 2008;13:494-504.

27 Burgos PI, Alarcón GS, McGwin Jr. G, et al. Disease activity and damage are not associated with increased levels of fatigue in systemic lupus erythematosus patients from a multiethnic cohort: LXVII, *Arthritis Care Res* 2009;61:1179-86.

28 McBeth J, Prescott G, Scotland G, et al. Cognitive behavior therapy, exercise, or both for treating chronic widespread pain, *Arch Intern Med* 2012;172:48-57.

29 Mohr DC, Vella L, Hart S, et al. The effect of telephone-administered psychotherapy on symptoms of depression and attrition: A meta-analysis, *Clin Psychol Sci Pract* 2008;15:243-53.

30 Druce KL, Jones GT, Macfarlane GJ, et al. Patients receiving anti-TNF therapies experience clinically important improvements in RA-related fatigue: results from the British Society for Rheumatology Biologics Register for Rheumatoid Arthritis, *Rheumatology (Oxford)* 2015;54:964-71.

31 White PD, Goldsmith K, Johnson AL, et al. Comparison of adaptive pacing therapy, cognitive behaviour therapy, graded exercise therapy, and specialist medical care for chronic fatigue syndrome (PACE): A randomised trial, *Lancet* 2011;377:823-36.

32 Hewlett S, Ambler N, Almeida C, et al. Protocol for a randomised controlled trial for Reducing Arthritis Fatigue by clinical Teams (RAFT) using cognitive-behavioural approaches, *BMJ Open* 2015;5.

33 Aletaha D, Neogi T, Silman AJ, et al. 2010 Rheumatoid arthritis classification criteria: An American College of Rheumatology/European League Against Rheumatism collaborative initiative, *Arthritis Rheum* 2010;62:2569-81.

For peer review only

34 Arnett FC, Edworthy SM, Bloch DA, et al. The american rheumatism association 1987 revised criteria for the classification of rheumatoid arthritis, *Arthritis Rheum* 1988;31:315-24.

35 Hochberg MC. Updating the American College of Rheumatology revised criteria for the classification of systemic lupus erythematosus. *Arthritis Rheum* 1997;40:1725.

36 Rudwaleit M, Landewé R, Van Der Heijde D, et al. The development of Assessment of SpondyloArthritis international Society classification criteria for axial spondyloarthritis (part I): Classification of paper patients by expert opinion including uncertainty appraisal, *Ann Rheum Dis* 2009;68:770-6.

37 Zigmond AS, Snaith RP. The hospital anxiety and depression scale, *Acta Psychiatr Scand* 1983;67:361-70.

38 Arthritis Research UK. Fatigue and Arthritis - Self-help and daily living. *2269/FATIG/14-1* 2014:1-32.

39 Cella M, Chalder T. Measuring fatigue in clinical and community settings, *J Psychosom Res* 2010;69:17-22.

40 Krupp LB, Larocca NG, Muir Nash J, et al. The fatigue severity scale: Application to patients with multiple sclerosis and systemic lupus erythematosus, *Arch Neurol* 1989;46:1121-3.

41 Nicklin J, Cramp F, Kirwan J, et al. Measuring fatigue in rheumatoid arthritis: a cross-sectional study to evaluate the Bristol Rheumatoid Arthritis Fatigue Multi-Dimensional questionnaire, visual analog scales, and numerical rating scales. *Arthritis Care Res (Hoboken)* 2010;62:1559-68.

42 Ware Jr. JE, Kosinski M, Keller SD. A 12-Item Short-Form Health Survey: Construction of Scales and Preliminary Tests of Reliability and Validity, *Med Care* 1996;34:220-33.

43 McCaffery M, Beebe A. Pain: Clinical manual for nursing practice. *Pain: Clinical Manual for Nursing Practice* 1989:1-353.

44 Jenkins CD, Stanton B-, Niemcryk SJ, et al. A scale for the estimation of sleep problems in clinical research, *J Clin Epidemiol* 1988;41:313-21.

45 Reilly MC, Zbrozek AS, Dukes EM. The Validity and Reproducibility of a Work Productivity and Activity Impairment Instrument, *Pharmacoeconomics* 1993;4:353-65.

46 Katz PP, Radvanski DC, Allen D, et al. Development and validation of a short form of the valued life activities disability questionnaire for rheumatoid arthritis, *Arthritis Care Res* 2011;63:1664-71.

47 Broadbent E, Petrie KJ, Main J, et al. The Brief Illness Perception Questionnaire, *J Psychosom Res* 2006;60:631-7.

For peer review only - http://bmjopen.bmj.com/site/about/guidelines.xhtml

48 Skerrett TN, Moss-Morris R. Fatigue and social impairment in multiple sclerosis: The role of patients' cognitive and behavioral responses to their symptoms, *J Psychosom Res* 2006;61:587-93.

49 Wolfe F, Clauw DJ, Fitzcharles M-, et al. Fibromyalgia criteria and severity scales for clinical and epidemiological studies: A modification of the ACR preliminary diagnostic criteria for fibromyalgia, *J Rheumatol* 2011;38:1113-22.

50 Charlson ME, Pompei P, Ales KL, et al. A new method of classifying prognostic comorbidity in longitudinal studies: development and validation, *J Chronic Dis* 1987;40:373-83.

51 Siconolfi SF, Garber CE, Lasater TM, et al. A simple, valid step test for estimating maximal oxygen uptake in epidemiologic studies, *Am J Epidemiol* 1985;121:382-90.

52 Larsen DL, Attkisson CC, Hargreaves WA, et al. Assessment of client/patient satisfaction: Development of a general scale, *Eval Program Plann* 1979;2:197-207.

53 Gale NK, Heath G, Cameron E, et al. Using the framework method for the analysis of qualitative data in multi-disciplinary health research, *BMC Med Res Methodol* 2013;13.

54 Patton MQ. Qualitative research & evaluation methods. Thousand Oaks, California: Sage Publications 2002.

55 Braun V, Clarke V. Using thematic analysis in psychology, *Qual Res Psychol* 2006;3:77-101.

56 Schulz KF, Altman DG, Moher D, et al. CONSORT 2010 statement: updated guidelines for reporting parallel group randomised trials, *BMJ* 2010;340:c332.

57 Al-Janabi H, Flynn TN, Coast J. Development of a self-report measure of capability wellbeing for adults: The ICECAP-A, *Qual Life Res* 2012;21:167-76.

58 McNamee P, Mendolia S. The effect of chronic pain on life satisfaction: Evidence from Australian data, *Soc Sci Med* 2014;121:65-73.

Figure 1 Study flowchart

Patients diagnosed with IRD will be invited by post and asked to return the pre-study screening questions. Screening is a two-step process using screening questions and review of medical records to establish fatigue state and identify other exclusion criteria. Potential participants will be contacted by phone to verify eligibility criteria and invited to the baseline assessment visit. After obtaining informed consent, their eligibility is confirmed (including fatigue state as well as determination of TSH, Hb

For peer review only

For peer review only - http://bmjopen.bmj.com/site/about/guidelines.xhtml

and eGFR values if these were not available in the medical notes), baseline data are collected and participants are randomised into the study.

At baseline, patients are also given a participant information sheet about the MRI sub-study. Randomised participants will be contacted by the Trial Office in Aberdeen if they are also eligible to take part in the MRI sub-study.

CBA, cognitive behavioural approach; eGFR, estimated glomerular filtration rate; Hb, haemoglobin; IRD, inflammatory rheumatic disease; PEP, personalised exercise programme; TSH, thyroid stimulating hormone; UC, usual care

Figure 2 Timeline intervention and assessment

Figure 3 Standard Protocol Items Recommendations for Interventional Trials (SPIRIT) Schedule of enrolment, interventions and assessments

For peer review only - http://bmjopen.bmj.com/site/about/guidelines.xhtml

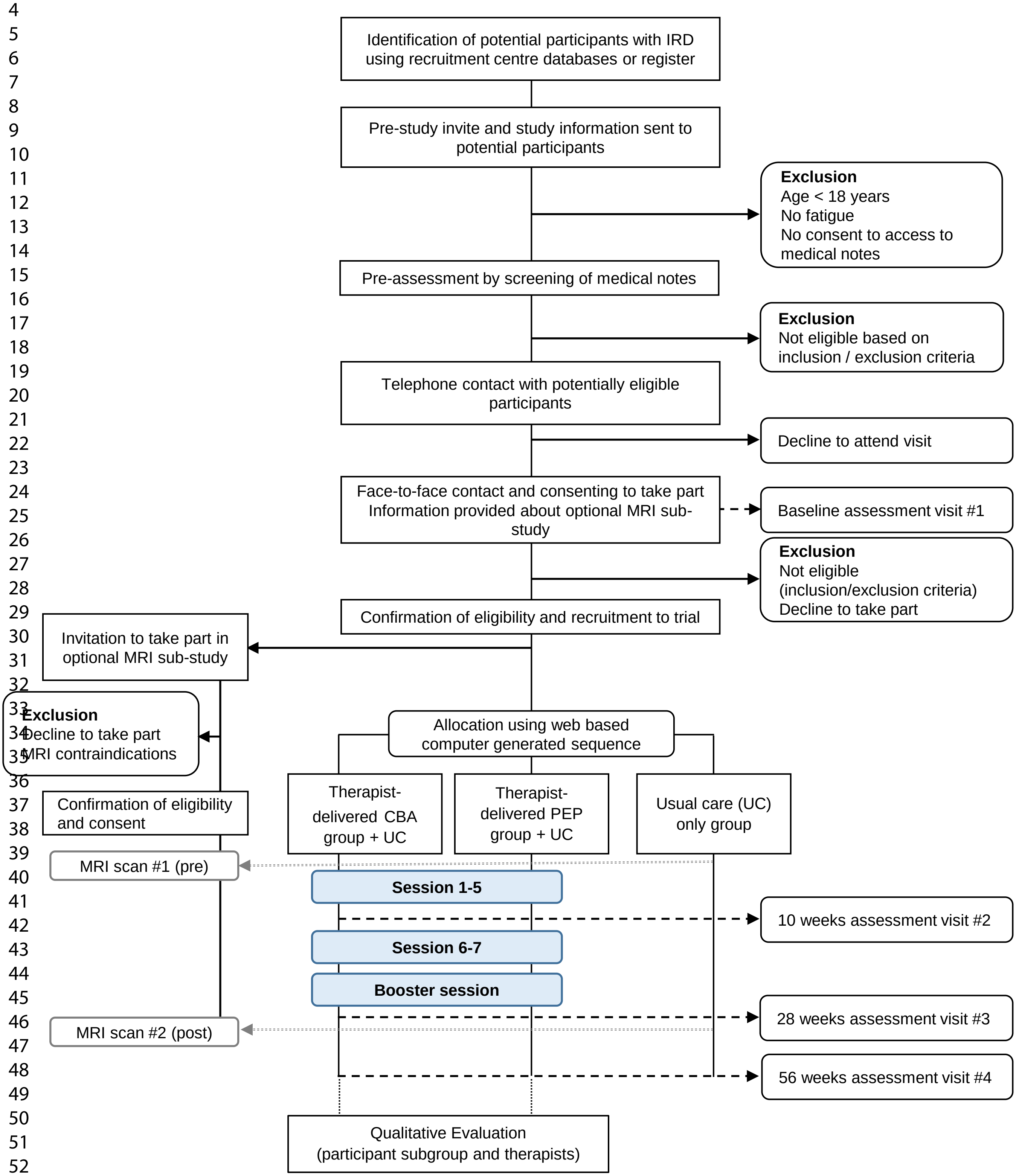
Identification of potential participants with IRD using recruitment centre databases or register
Pre-study invite and study information sent to potential participants
Exclusion
Age < 18 years
No fatigue
No consent to access to medical notes
Pre-assessment by screening of medical notes
Exclusion
Not eligible based on inclusion / exclusion criteria
Telephone contact with potentially eligible participants
Decline to attend visit
Face-to-face contact and consenting to take part
Information provided about optional MRI sub-study
Baseline assessment visit #1
Exclusion
Not eligible (inclusion/exclusion criteria)
Decline to take part
Confirmation of eligibility and recruitment to trial
Invitation to take part in optional MRI sub-study
Exclusion
Decline to take part
MRI contraindications
Allocation using web based computer generated sequence
Confirmation of eligibility and consent
Therapist-delivered CBA group + UC
Therapist-delivered PEP group + UC
Usual care (UC) only group
MRI scan #1 (pre)
Session 1-5
10 weeks assessment visit #2
Session 6-7
Booster session
28 weeks assessment visit #3
MRI scan #2 (post)
56 weeks assessment visit #4
Qualitative Evaluation
(participant subgroup and therapists)

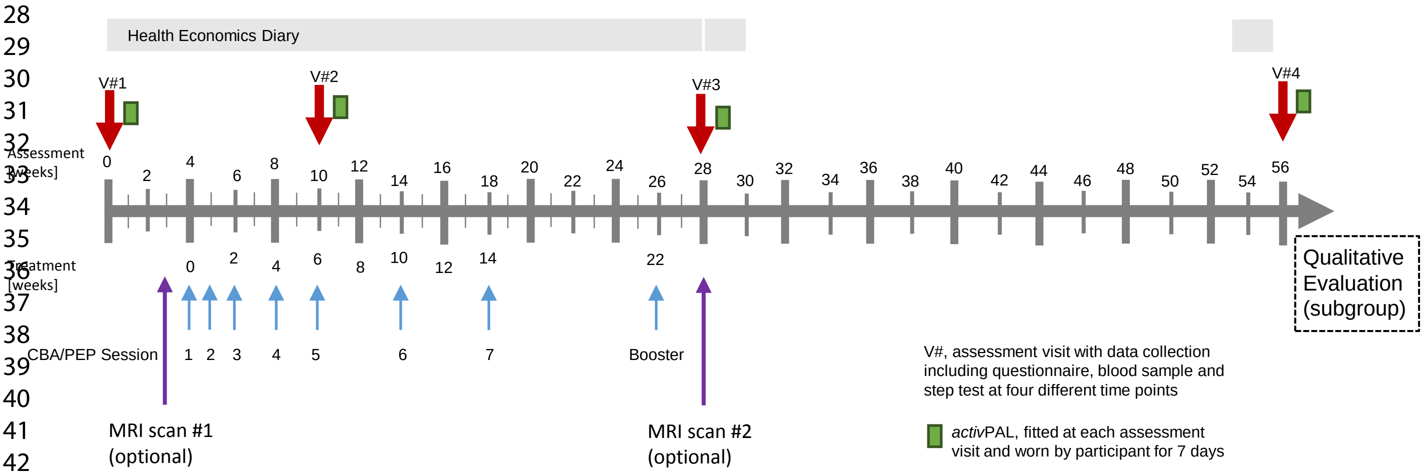
Health Economics Diary
V#1
V#2
V#3
V#4
Assessment [weeks]
0 2 4 6 8 10 12 14 16 18 20 22 24 26 28 30 32 34 36 38 40 42 44 46 48 50 52 54 56
Treatment [weeks]
0 2 4 6 8 10 12 14 22
CBA/PEP Session
1 2 3 4 5 6 7 Booster
MRI scan #1 (optional)
MRI scan #2 (optional)
Qualitative Evaluation (subgroup)
V#, assessment visit with data collection including questionnaire, blood sample and step test at four different time points
activPAL, fitted at each assessment visit and worn by participant for 7 days

Figure 3. Example template of recommended content for the schedule of enrolment, interventions, and assessments.

	**STUDY PERIOD**					
	**Enrolment**	**Allocation**	**Post-allocation**			**Qualitative Evaluation**
**TIMEPOINT**	***Pre-study***	***Visit 1 Baseline***	***Visit 2 10 weeks***	***Visit 3 28 weeks***	***Visit 4 56 weeks***	***Interview > 56 weeks***
**ENROLMENT:**						
**Pre-study invite**	X					
**Eligibility screen**	X	X				
**Informed consent**		X				X
**Allocation**		X				
**INTERVENTIONS[a]:**						
Usual Care alone			◆———	———◆		
CBA + Usual Care			◆———	———◆		
PEP + Usual Care			◆———	———◆		
**ASSESSMENTS:**						
Case Report Form	X	X	X	X	X	
Current fatigue status	X	X				
Medical history	X	X				
Comorbidities and pharmacological therapies (current)		X	X	X	X	
Participant reported outcomes[b]		X	X	X	X	
Aerobic fitness test (step test)		X	X	X	X	
Activity monitor		X	X	X	X	
Health Care Diary		◆———	———	———◆	X	

For peer review only

Evaluation (quantitative) [c]			X	X		
Nested sub-study [d] ***Evaluation (qualitative)***						X
Nested sub-study [d] ***MRI scan (optional)***		X		X		

[a] Start of active CBA and PEP interventions expected to start between 2 and 8 weeks post-randomisation with an average of 4 weeks after baseline.
[b] Full list of questionnaires see outcomes.
[c] Full list of quantitative evaluation measures see Table 1 study matrix
[d] Invitation and consent separate from main trial

For peer review only

Additional File 1 SWAT 24

Background

Recruiting and retaining participants for randomised trials can be extremely difficult. It is likely that less than 50% of trials meet their recruitment target, or meet their target without extending the length of the trial [1-3]. Moreover, poor recruitment can lead to an underpowered study, which may report clinically relevant effects to be statistically non-significant. A non-significant finding increases the risk that an effective intervention will be abandoned before its true value is established, or that there will be a delay in demonstrating this value while more studies or meta-analyses are done. Moreover, if non-responses to the study invitation differ between the patients with diagnosis of RA, SLE and AxSpA, a systematic bias may be introduced that may undermine confidence in the results of the trial. Finally, poor recruitment and subsequently retention can lead to a trial being extended, increasing costs.

Trialists recognise the challenge and use many interventions to improve recruitment and retention but it is generally difficult to predict their effect. The Cochrane systematic review of strategies to improve recruitment [4] and the Cochrane review of strategies it improve retention [5] both found only a handful of interventions with high quality evidence of benefit. Given how central recruitment and retention are to all trials, it is crucial that more rigorous evaluations of recruitment and retention interventions are done.

Rationale for SWAT

One way of doing this is to do a Study Within a Trial (SWAT) [6]. A SWAT provides a protocol for the evaluation of an intervention to improve some part of the trial process, such as recruitment or retention. This evaluation is then embedded within a host trial, such as LIFT. Several teams can follow the same SWAT protocol, meaning the results can be combined in a meta-analysis. This coordinated and collaborative approach means trialists will have faster access to high-quality evidence to inform their trial design, conduct, analysis and reporting decisions.

The SWAT 24 which describes the use of a theory-based cover letter was initially developed to increase response rates of questionnaires sent during follow-up to collect outcome data direct from participants. A low response rate to these questionnaires puts the validity and generalisability of the trial results in jeopardy. Since returning the questionnaire is a behaviour, this opens up the possibility of designing a behaviour change intervention to influence the willingness of participants to do that behaviour. We propose to use SWAT 24 in LIFT to improve response rates to the pre-study invitation letter used to make initial contact with potential participants identified as described in the main protocol to explore interest and eligibility. The SWAT 24 study is part of the Trial Forge initiative to improve trial efficiency [7].

Objective for SWAT 24

To assess the effects of a theory-based cover letter on response rate to a pre-study invite to explore interest and eligibility

For peer review only

Outcome

Primary outcome:

Response rate

Secondary outcomes:

Response time

Consent rate

Study retention

Intervention adherence

Intervention

The Theoretical Domains Framework (TDF) is a tool for identifying theoretical targets for behaviour change interventions [8]. The TDF and behaviour change techniques were used by the IQuaD trial team [9] to produce a template that trial teams can use to structure a theory-informed cover letter.

Comparator

A standard cover letter

Method for allocating to intervention or comparator

Participating study centres will be randomly allocated to send the standard letter or the theory-informed letter.

Analysis plan

The primary analysis is the difference in primary and secondary outcomes between those receiving the theory-based cover letter and those receiving the standard cover letter.

References

[1] Ben G O Sully, Julious SA, Nicholl J. A reinvestigation of recruitment to randomised, controlled, multicenter trials: a review of trials funded by two UK funding agencies. Trials 2013, 14: 166.

[2] McDonald AM, Knight RC, Campbell MK, Entwistle VA, Grant AM, Cook JA, Elbourne DR, Francis D, Garcia J, Roberts I, Snowdon C. What influences recruitment to randomised controlled trials? A review of trials funded by two UK funding agencies. Trials 2006;7:9.

For peer review only

[3] Foy R, Parry J, Duggan A, Delaney B, Wilson S, Lewin-van den Broek NTh, Lassen A, Vickers L, Myres P. How evidence-based are recruitment strategies for randomized controlled trials in primary care? Experience from seven studies. Family Practice 2003;20:83-92.

[4] Treweek S, Mitchell E, Pitkethly M, Cook J, Kjeldstrøm M, Johansen M, Taskila TK, Sullivan F, Wilson S, Jackson C, Jones R, Lockhart P. Strategies to improve recruitment to randomised controlled trials. Cochrane Database of Systematic Reviews 2010, Issue 4. Art. No.: MR000013. DOI: 10.1002/14651858.MR000013.pub5.

[5] Brueton VC, Tierney J, Stenning S, Harding S, Meredith S, Nazareth I, Rait G. Strategies to improve retention in randomised trials. Cochrane Database of Systematic Reviews 2013, Issue 12. Art. No.: MR000032. DOI: 10.1002/14651858.MR000032.pub2.

[6] Smith V, Clarke M, Devane D, Begley C, Shorter G, Maguire L. SWAT 1: what effects do site visits by the principal investigator have on recruitment in a multicentre randomized trial? Journal of Evidence-based Medicine 2013; 6:136–7.

[7] Treweek S, Altman DG, Bower P, Campbell M, Chalmers I, Cotton S, et al. Making randomised trials more efficient: report of the first meeting to discuss the Trial Forge platform. Trials 2015; 16:261.

[8] Michie et al. Making psychological theory useful for implementing evidence based practice. Qual &Safety in Health Care 2005; 14:26–33

[9] Jan E Clarkson, Craig R Ramsay, Paul Averely, D Bonetti, Dwayne Boyers, Louise Campbell, Graham R Chadwick, Anne Duncan, Andrew Elders, Jill Gouick, Nigel B Pitts, Marjon van der Pol, David Nj Ricketts, Margaret K Ross, James G Steele, Moira Swan, Martin Tickle, Pauline D Watt, Helen V Worthington, Linda Young. IQuaD dental trial; improving the quality of dentistry: a multicentre randomised controlled trial comparing oral hygiene advice and periodontal instrumentation for the prevention and management of periodontal disease in dentate adults attending dental primary care. BMC Oral Health 10/2013; 13(1):58.

For peer review only

Table 1. Summary of outcome measures for each time point and their source (Study Matrix)

	Source	Items		**Proposed assessment [weeks]**			
			S	0	10	28	56
**Demographic data**							
Date of birth, gender, marital status, employment status, level of education	Q	5		✓			
**Characteristics of study population**							
Overall health	Q	1		✓			
Physical activity (typical self-reported)	Q	1		✓			
Experience of fatigue for more than 3 month	Q / CRF	1	✓	✓			
Average level of fatigue	Q / CRF	1	✓	✓			
Thyroid function test	B or MR			✓			
Urea and electrolytes	B or MR			✓			
Full blood count	B or MR			✓			
Serological status	MR			✓			
Erosive status	MR			✓			
Disease duration	MR			✓			
Presence of other co-morbidities (Charlson Index)	MR/CRF			✓	✓	✓	✓
History of suicide attempts	MR/CRF			✓			
Inflammation (CRP/ESR)	B			✓	✓	✓	✓
Current pharmacological therapies	MR			✓	✓	✓	✓
Blood pressure	T			✓			
**Primary Outcome**							
Chalder Fatigue Scale (Likert scoring)	Q	11		✓	✓	✓	✓
Fatigue Severity Scale	Q	9		✓	✓	✓	✓

For peer review

	Source	Items		Proposed assessment [weeks]			
			S	0	10	28	56
**Secondary Outcomes**							
Bristol Rheumatoid Arthritis Fatigue Multi-Dimensional Questionnaire (fatigue)	Q	20		✓	✓	✓	✓
Hospital anxiety and depression scale (anxiety and depression)	Q	14		✓	✓	✓	✓
Short Form-12	Q	12		✓	✓	✓	✓
Pain numerical rating scale	Q	1		✓	✓	✓	✓
Sleep problem scale	Q	4		✓	✓	✓	✓
Work Productivity and Activity Impairment Questionnaire	Q	6		✓	✓	✓	✓
Valued Life Activities Scale (short 14 items)	Q	14		✓	✓	✓	✓
Global outcome	Q	1			✓	✓	✓
**Additional mediator/moderator data**							
Cognitions and behaviours							
Brief Illness Perception Questionnaire	Q	9		✓	✓	✓	✓
Behavioural Response to Illness Questionnaire	Q	21		✓	✓	✓	✓
Clinical							
Presence of fibromyalgia	CRF	8		✓			✓
Disease activity (self-reported)	CRF	2		✓	✓	✓	✓
Disease activity DAS28 for RA (mandatory), other disease specific activity measures (non-mandatory)				✓	✓	✓	✓
Physical							
Physical activity profiles, over a 7 day period	T			✓	✓	✓	✓
Quantifying aerobic fitness (step) test (weight, VO_2 max and Borg Rating of	T			✓	✓	✓	✓

For peer review

	Source	Items		Proposed assessment [weeks]			
			S	0	10	28	56
Perceived Exertion)							
Neuroimaging (optional)							
Multi-modal MRI scan	SC			x		x	
**Quantitative evaluation**							
Patient preference	Q	2		✓			
Patient adherence (attendance records)	SR			x	x	x	
Patient engagement and adherence (telephone call)	Q	3			x	x	
Patent engagement and adherence (therapist perspective)	Q	2			x	x	
Patient acceptability (Client Satisfaction Questionnaire)	Q	8				✓	
**Qualitative process evaluation**							
Qualitative evaluation in participants (5% sample)	I						
Qualitative evaluation in therapists (all)	I						
**Economic evaluation**							
Health care costs from participants per diaries	D			✓	✓	✓	✓
Additional (personal) costs for participant per diaries	D			✓	✓	✓	✓
Cost associated with delivery of therapy	SR			✓	✓	✓	✓
Health related quality of life using SF-12 for calculation of QALY				✓	✓	✓	✓
Changes in well-being data using ICECAP	Q	5		✓	✓	✓	✓
Changes in life satisfaction	Q	1		✓	✓	✓	✓

For peer review only

	Source	Items		Proposed assessment [weeks]			
			S	0	10	28	56
**Optional blood sample for future ethically approved research**							
1 x PAXgene RNA				✓	✓	✓	
1 x PAXgene DNA				✓			
1 x serum				✓	✓	✓	

Key: B, blood sample taken specifically for LIFT; **CRF**, part of case report form completed by research nurse during visit; **D**, separate diary for participant to keep; **I**, interviews performed after the last follow-up visit; **ICECAP**, Investigating Choice Experiments for the preference of older people CAPability measures for adults; **MR**, information extracted from medical record; **Q**, data derived from questionnaire; **QALY**, Quality Adjusted Life Years; **S**, information obtained during pre-study invite; **SC**, Scanner; **SR**, information extracted from LIFT study records and logs; **T**, test done specifically for LIFT; **x**, outcome collection at same time frame but separate from assessment visit

For peer review only

Appendix 2: Delineating the neural mediators of inflammatory rheumatic disease (An optional LIFT sub-study)

Background

One of the objectives of the LIFT trial is to understand the mediators of treatment effect which will enable optimising of interventions and inferences to be made regarding the mechanisms of fatigue. We are collecting putative patient reported, clinical and physical activity profile mediator data. In addition we would like to provide the option for participants to provide their neuroimaging data by undertaking additional MRI scans of their brain.

Neuroimaging has provided consistent mechanistic insights into fatigue, reinforcing our epidemiological investigations of RA related fatigue which have identified strong associations with central factors such as mental health and cognitive dysfunctions but not peripheral measures of inflammation[4,5]. We were the first to test these methods in a fatigued chronic inflammatory disease cohort. We identified multiple neural correlates of fatigue in patients with vasculitis using different structural and functional magnetic resonance imaging (MRI) modes [6,7] and we have since observed similar fatigue specific findings in RA.

Our original studies were small (n=12) and cross-sectional but did implicate a potential role for a striato-thalamo-frontal network. Among fatigued vasculitis patients, we observed an apparent overuse of the cingulum and fornix white matter tracts, as identified by Diffusion Tensor Imaging (DTI), which coincided with the high functional activity within some of their source and destination grey matter structures, specifically the thalamus, medial globus pallidus, medial frontal and cingulate gyri and paracentral lobule.

Our most recent pilot was larger and although uncontrolled included a repeat multi-modal scan at 6 months that enabled the longitudinal evaluation of fatigue in the context of standard care (which did not include fatigue specific therapy). Of those n=54 completing follow up, n=22 reported modest, albeit clinically relevant, improvements in their fatigue. Interestingly, widespread reductions in cortical grey matter volumes were measured, using voxel-based morphometry (VBM), among the non-improvers at follow-up however no such volume changes were observed among improvers. In contrast, sub-cortical grey matter volumes exhibited large significant increases in non-improvers. The sub-cortical grey matter volumes of improvers also increased, although the changes were small in comparison.

In terms of white matter integrity, as measured by DTI, widespread abnormalities were observed among improvers. Within this group, significantly reduced fractional anisotropy (FA) was measured at follow-up compared to baseline in major white matter tracts. Strikingly, no significant longitudinal FA changes were measured within the non-improvers group. Similarly, improvers- and not non-improvers- evidenced widespread imbalances in functional connectivity imbalance over time.

Overall, these data strongly associate central neural pathways in RA related fatigue. Although the different MRI metrics provide complementary evidence which implicate frontal networks, a targeted approach (e.g. with non-invasive neuromodulation devices) will demand much greater knowledge of the precise culprit frontal regions, moreover several non-frontal regions seem also to be relevant. Until now, no studies have been adequately designed to pin-point those brain regions which mediate changes in fatigue (and so demonstrate causal potential).

The problem with our existing studies is that they were either cross-sectional, or longitudinally followed individuals who had spontaneous changes in their fatigue (thus the changes in fatigue from one time point to the next were modest). We propose that the best way to identify which of these brain regions are most important in mediating fatigue is to perform controlled longitudinal imaging studies in individuals prior to and then following an intervention that reliably improves fatigue in most individuals.

This Lessening the Impact of Fatigue Trial (LIFT) sub-study provides a timely opportunity to address this research void.

Sub-study objective

- Which functional and structural brain mediators best explain fatigue improvement and are they potentially trans-cranially accessible?

Study design

An optional nested mechanistic observational sub-study within the Lessening the Impact of Fatigue Trial (LIFT).

Participants

All consenting LIFT participants will be invited to participate in this optional sub-study which involves an additional MRI brain scan prior to and 26 weeks after their first treatment session (if CBA or PEP) or within 8 weeks of randomisation and 6 months thereafter if usual care. The only additional exclusion criteria is any contra-indications to MRI scanning (e.g. pacemaker).

Participant selection

At the baseline visit, all participants will be provided a Participant Information Sheet on this sub-study. The research team will then contact the participants a few days later to establish interest and the absence of MRI contraindications. If suitable and interested, the participant will be offered an appointment to attend their nearest participating MRI research facility (Glasgow, Edinburgh or Aberdeen) within a month. A research team member, recorded in the Delegation Log and with GCP training, will be responsible for taking additional full written informed consent (specific to this sub-study) on attendance at the imaging centre prior to the MRI assessment and then conduct a final MRI safety screen.

Participant withdrawal

All participants will be free to withdraw at any time from the MRI sub-study, without giving reasons and without prejudicing further treatment or their participation in the LIFT trial.

For peer review only

MRI assessment

Prior to entering the scanner, subjects will have the opportunity to practice a cognitive task required for the standard fMRI aspect of analysis. As with our previous work[6], the validated PASAT will be employed to transiently fatigue the subject. The task is a measure of cognitive function; specifically auditory processing, calculation, working memory and attention. Participants will be asked to listen to a series of numbers ranging from 1 to 9. They are required to sum consecutive numbers (i.e. the first to the second, the second to the third etc.) and to record, via a button press, every occasion two consecutive numbers sum to the number 10. Concurrently, they will be asked to focus on a computer screen displaying three boxes containing random, rapidly changing numbers. This visual stimulus is intended to distract the participants from the auditory task and hence increase difficulty. They will be instructed not to process the visual numbers in any way.

Participants will then be asked to lie supine in the 3T Phillips Achieva X-series MRI scanner in Aberdeen or the equivalent scanner in Edinburgh or Glasgow. The multi-modal MR will consist of structural and functional sequences:

Structural- We will collect images to allow volumetric analysis. We will also acquire images to allow determination of white matter hyperintensity lesion load and measures of white matter structural integrity (e.g. DTI).

Functional Imaging- Images sensitive to BOLD contrast will be acquired during rest to investigate metrics such as intrinsic network connectivity as well as during the PASAT task (3x3minute periods interspersed by 30s rest periods).

In total these can will take approximately 45 minutes to conduct and will be repeated at approx. 6 months (when we predict to observe the greatest effect from the interventions).

Analysis

Following pre-processing of the MRI data the following analysis will be undertaken which will integrate data which will have been collected as part of the parent trial:

Longitudinal comparisons (paired t-tests as implemented by SPSS for ROI based variables and Freesurfer for voxel based variables) of structural and functional change indices in relation to subjects' change in fatigue will be performed. Putative confounders will be individually introduced as co-variates of interest. The individual analyses will focus upon those regions of interest previously identified by our studies, but since we recognise that these are not comprehensive we will also conduct agnostic (data-driven) whole brain analyses. All analyses will be adjusted for multiple testing.

Having identified and validated key neural areas, group differences in mediators of treatment effects on outcomes will be assessed via mediation analysis methods in order to tackle the secondary objective. These involve causal inference methods, such as structural equation modelling, to account for measurement error in the imaging data, and repeated measures to allow for the inclusion of all available data. Those resultant neural mediators which are common to both interventions and accessible to non-invasive neuromodulation will serve as our future therapeutic targets.

Finally we will be using whole brain statistical pattern recognition techniques on the neuroimaging data and mediation effects identified in answer to the previous objective. In the Pattern Recognition

For peer review only

for Neuroimaging Toolbox (PRoNTo) brain scans are treated as spatial patterns and statistical learning models are used to identify statistical properties of the data that can be used to discriminate between, or classify, experimental groups of subjects.

Sample size

We aim to recruit 120 participants (who will have been randomised to receive either usual care alone, CBA in addition to usual care or PEP in addition to usual care in the parent trial).

Data handling

MRI scan data will be stored in an anonymised format in the University of Aberdeen and Edinburgh imaging archive system on the university drive, with a back-up disc stored in a fireproof safe. The code for the images will be held on a separate computer relating the patient information to the participant ID. The participant ID will be used on the MRI images. Images may be stored on disc in anonymised format for research team discussions out-with the imaging department.

Safety assessments

The MRI scanner is very safe and does not expose participants to any harmful radiation. Given its reliance on a strong magnetic field, it is essential that certain metallic instruments/objects are not taken into the scan room.

This is avoided by:

1) All participants are clothed in 'theatre greens' so to avoid the danger of concealed metal objects within clothes.

2) Patients will be screened for absolute exclusion to MRI scanning.

In addition, all participants must undertake a strict and comprehensive checklist prior to scanning. This includes questions about heart valves, pacemakers and other potential metallic implants.

If a participant becomes distressed during the MRI scan, he/she will be able to access to a "panic button" which will immediately terminate the procedure.

For peer review only

Sub-study matrix

The study connects to the 1-year LIFT recruitment phase which will begin in August 2017 (month 0).

Study steps	-3-0 m	0-6 m	6-12 m	12-18 m	18-24 m	24-30 m
Governance approvals						
RA Subject recruitment						
MRI scan #1 (pre-intervention)						
MRI scan #2 (post-intervention)						
Data processing, analysis & report						

References

1. Hewlett S, Cockshott Z, Byron M, et al. Patients' perceptions of fatigue in rheumatoid arthritis: overwhelming, uncontrollable, ignored. *Arthritis and rheumatism.* 2005;53(5):697-702.
2. Davies H, Brophy S, Dennis M, Cooksey R, Irvine E, Siebert S. Patient perspectives of managing fatigue in Ankylosing Spondylitis, and views on potential interventions: a qualitative study. *BMC Musculoskelet Disord.* 2013;14:163.
3. Patton MQ, Patton MQQe, research m. *Qualitative research & evaluation methods.* 3rd ed. ed. Thousand Oaks, Calif. ; London: Sage; 2002.
4. Druce KL, Jones GT, Macfarlane GJ, Basu N. Determining pathways to improvements in fatigue in rheumatoid arthritis: results from the British Society for Rheumatology Biologics Register for Rheumatoid Arthritis. *Arthritis & Rheumatology.* 2015;67:2303-2310.
5. Druce KL, Jones GT, Macfarlane GJ, Basu N. The changes in pain which mediate Reductions in fatigue likely reflect central not peripheral mechanisms: Results from the BSRBR-RA. *Arthritis Care & Research.* 2016.
6. Basu N, Murray AD, Jones GT, Reid DM, Macfarlane GJ, Waiter GD. Neural correlates of fatigue in granulomatosis with polyangiitis: a functional magnetic resonance imaging study. *Rheumatology.* 2014;53:2080-2087.
7. Basu N, Murray AD, Jones GT, Reid DM, Macfarlane GJ, Waiter GD. Fatigue-related brain white matter changes in granulomatosis with polyangiitis. *Rheumatology.* 2013:ket135.

For peer review only

SPIRIT

STANDARD PROTOCOL ITEMS: RECOMMENDATIONS FOR INTERVENTIONAL TRIALS

SPIRIT 2013 Checklist: Recommended items to address in a clinical trial protocol and related documents*

Section/item	Item No	Description	Addressed on page number
**Administrative information**			
Title	1	Descriptive title identifying the study design, population, interventions, and, if applicable, trial acronym	1
Trial registration	2a	Trial identifier and registry name. If not yet registered, name of intended registry	5
	2b	All items from the World Health Organization Trial Registration Data Set	n/a
Protocol version	3	Date and version identifier	31
Funding	4	Sources and types of financial, material, and other support	30
Roles and responsibilities	5a	Names, affiliations, and roles of protocol contributors	1-3, 31
	5b	Name and contact information for the trial sponsor	30
	5c	Role of study sponsor and funders, if any, in study design; collection, management, analysis, and interpretation of data; writing of the report; and the decision to submit the report for publication, including whether they will have ultimate authority over any of these activities	30
	5d	Composition, roles, and responsibilities of the coordinating centre, steering committee, endpoint adjudication committee, data management team, and other individuals or groups overseeing the trial, if applicable (see Item 21a for data monitoring committee)	26

For peer review only

For peer review only - http://bmjopen.bmj.com/site/about/guidelines.xhtml

**Introduction**			
Background and rationale	6a	Description of research question and justification for undertaking the trial, including summary of relevant studies (published and unpublished) examining benefits and harms for each intervention	5-8
	6b	Explanation for choice of comparators	5-8
Objectives	7	Specific objectives or hypotheses	8
Trial design	8	Description of trial design including type of trial (eg, parallel group, crossover, factorial, single group), allocation ratio, and framework (eg, superiority, equivalence, noninferiority, exploratory)	9
**Methods: Participants, interventions, and outcomes**			
Study setting	9	Description of study settings (eg, community clinic, academic hospital) and list of countries where data will be collected. Reference to where list of study sites can be obtained	9
Eligibility criteria	10	Inclusion and exclusion criteria for participants. If applicable, eligibility criteria for study centres and individuals who will perform the interventions (eg, surgeons, psychotherapists)	9-11
Interventions	11a	Interventions for each group with sufficient detail to allow replication, including how and when they will be administered	13-16
	11b	Criteria for discontinuing or modifying allocated interventions for a given trial participant (eg, drug dose change in response to harms, participant request, or improving/worsening disease)	n/a
	11c	Strategies to improve adherence to intervention protocols, and any procedures for monitoring adherence (eg, drug tablet return, laboratory tests)	16, 19
	11d	Relevant concomitant care and interventions that are permitted or prohibited during the trial	13
Outcomes	12	Primary, secondary, and other outcomes, including the specific measurement variable (eg, systolic blood pressure), analysis metric (eg, change from baseline, final value, time to event), method of aggregation (eg, median, proportion), and time point for each outcome. Explanation of the clinical relevance of chosen efficacy and harm outcomes is strongly recommended	16-20, Supplement Table 1
Participant timeline	13	Time schedule of enrolment, interventions (including any run-ins and washouts), assessments, and visits for participants. A schematic diagram is highly recommended (see Figure)	9, 16, Figure 1, Figure 2, Figure 3

For peer review only - http://bmjopen.bmj.com/site/about/guidelines.xhtml

Sample size	14	Estimated number of participants needed to achieve study objectives and how it was determined, including clinical and statistical assumptions supporting any sample size calculations	22
Recruitment	15	Strategies for achieving adequate participant enrolment to reach target sample size	9
**Methods: Assignment of interventions (for controlled trials)**			
Allocation:			
Sequence generation	16a	Method of generating the allocation sequence (eg, computer-generated random numbers), and list of any factors for stratification. To reduce predictability of a random sequence, details of any planned restriction (eg, blocking) should be provided in a separate document that is unavailable to those who enrol participants or assign interventions	12
Allocation concealment mechanism	16b	Mechanism of implementing the allocation sequence (eg, central telephone; sequentially numbered, opaque, sealed envelopes), describing any steps to conceal the sequence until interventions are assigned	12
Implementation	16c	Who will generate the allocation sequence, who will enrol participants, and who will assign participants to interventions	12
Blinding (masking)	17a	Who will be blinded after assignment to interventions (eg, trial participants, care providers, outcome assessors, data analysts), and how	12-13
	17b	If blinded, circumstances under which unblinding is permissible, and procedure for revealing a participant's allocated intervention during the trial	n/a
**Methods: Data collection, management, and analysis**			
Data collection methods	18a	Plans for assessment and collection of outcome, baseline, and other trial data, including any related processes to promote data quality (eg, duplicate measurements, training of assessors) and a description of study instruments (eg, questionnaires, laboratory tests) along with their reliability and validity, if known. Reference to where data collection forms can be found, if not in the protocol	16-21
	18b	Plans to promote participant retention and complete follow-up, including list of any outcome data to be collected for participants who discontinue or deviate from intervention protocols	22

For peer review only - http://bmjopen.bmj.com/site/about/guidelines.xhtml

Data management	19	Plans for data entry, coding, security, and storage, including any related processes to promote data quality (eg, double data entry; range checks for data values). Reference to where details of data management procedures can be found, if not in the protocol	27
Statistical methods	20a	Statistical methods for analysing primary and secondary outcomes. Reference to where other details of the statistical analysis plan can be found, if not in the protocol	22-26
	20b	Methods for any additional analyses (eg, subgroup and adjusted analyses)	24-25
	20c	Definition of analysis population relating to protocol non-adherence (eg, as randomised analysis), and any statistical methods to handle missing data (eg, multiple imputation)	25
**Methods: Monitoring**			
Data monitoring	21a	Composition of data monitoring committee (DMC); summary of its role and reporting structure; statement of whether it is independent from the sponsor and competing interests; and reference to where further details about its charter can be found, if not in the protocol. Alternatively, an explanation of why a DMC is not needed	26-27
	21b	Description of any interim analyses and stopping guidelines, including who will have access to these interim results and make the final decision to terminate the trial	n/a
Harms	22	Plans for collecting, assessing, reporting, and managing solicited and spontaneously reported adverse events and other unintended effects of trial interventions or trial conduct	21
Auditing	23	Frequency and procedures for auditing trial conduct, if any, and whether the process will be independent from investigators and the sponsor	n/a
**Ethics and dissemination**			
Research ethics approval	24	Plans for seeking research ethics committee/institutional review board (REC/IRB) approval	31-32
Protocol amendments	25	Plans for communicating important protocol modifications (eg, changes to eligibility criteria, outcomes, analyses) to relevant parties (eg, investigators, REC/IRBs, trial participants, trial registries, journals, regulators)	n/a

For peer review only - http://bmjopen.bmj.com/site/about/guidelines.xhtml

Consent or assent	26a	Who will obtain informed consent or assent from potential trial participants or authorised surrogates, and how (see Item 32)	12
	26b	Additional consent provisions for collection and use of participant data and biological specimens in ancillary studies, if applicable	19, 20
Confidentiality	27	How personal information about potential and enrolled participants will be collected, shared, and maintained in order to protect confidentiality before, during, and after the trial	27
Declaration of interests	28	Financial and other competing interests for principal investigators for the overall trial and each study site	31
Access to data	29	Statement of who will have access to the final trial dataset, and disclosure of contractual agreements that limit such access for investigators	30
Ancillary and post-trial care	30	Provisions, if any, for ancillary and post-trial care, and for compensation to those who suffer harm from trial participation	n/a
Dissemination policy	31a	Plans for investigators and sponsor to communicate trial results to participants, healthcare professionals, the public, and other relevant groups (eg, via publication, reporting in results databases, or other data sharing arrangements), including any publication restrictions	30
	31b	Authorship eligibility guidelines and any intended use of professional writers	n/a
	31c	Plans, if any, for granting public access to the full protocol, participant-level dataset, and statistical code	30
**Appendices**			
Informed consent materials	32	Model consent form and other related documentation given to participants and authorised surrogates	n/a
Biological specimens	33	Plans for collection, laboratory evaluation, and storage of biological specimens for genetic or molecular analysis in the current trial and for future use in ancillary studies, if applicable	18-19

*It is strongly recommended that this checklist be read in conjunction with the SPIRIT 2013 Explanation & Elaboration for important clarification on the items. Amendments to the protocol should be tracked and dated. The SPIRIT checklist is copyrighted by the SPIRIT Group under the Creative Commons "Attribution-NonCommercial-NoDerivs 3.0 Unported" license.

For peer review only - http://bmjopen.bmj.com/site/about/guidelines.xhtml

BMJ Open

Protocol for a multi-centre randomised controlled parallel-group trial to compare the effectiveness of remotely delivered cognitive behavioural and graded exercise interventions with usual care alone to lessen the impact of fatigue in inflammatory rheumatic diseases (LIFT)

Journal:	*BMJ Open*
Manuscript ID	bmjopen-2018-026793.R1
Article Type:	Protocol
Date Submitted by the Author:	06-Nov-2018
Complete List of Authors:	Martin, Kathryn; University of Aberdeen, Epidemiology Group, Institute of Applied Health Sciences<br>Bachmair, Eva-Maria; University of Aberdeen, Epidemiology Group, Institute of Applied Health Sciences<br>Aucott, Lorna; University of Aberdeen , Centre of Healthcare and Randomised Trials (CHaRT), Health Services Research Unit<br>Dures, Emma; University of the West of England, Nursing and Midwifery<br>Emsley, Richard; King's College London, Department of Biostatistics and Health Informatics, Institute of Psychiatry, Psychology and Neuroscience<br>Gray, Stuart; University of Glasgow, Institute of Cardiovascular and Medical Sciences, BHF Glasgow Cardiovascular Research Centre<br>Hewlett, Sarah; University of the West of England,<br>Kumar, Vinod; Ninewells Hospital, NHS Tayside<br>Lovell, Karina; The University of Manchester, School of Health Sciences<br>Macfarlane, Gary; University of Aberdeen, Epidemiology Group, Institute of Applied Health Sciences<br>Maclennan, Graeme; University of Aberdeen, Centre of Healthcare and Randomised Trials (CHaRT), Health Services Research Unit<br>McNamee, Paul; University of Aberdeen<br>Norrie, John; Edinburgh Clinical Trials Unit, University of Edinburgh No. 9, Bioquarter<br>Paul, Lorna; Glasgow Caledonian University School of Health and Life Sciences,<br>Ralston, Stuart; University of Edinburgh, Rheumatology and Bone Disease<br>Siebert, Stefan; University of Glasgow, Institute of Infection, Immunity and Inflammation; NHS Greater Glasgow and Clyde,<br>Wearden, Alison; University of Manchester, School of Health Sciences<br>White, Peter; Queen Mary University of London,, Department of Psychological Medicine, Bartholomew's Hospital<br>Basu, Neil; University of Glasgow, Institute of Infection, Immunity and Inflammation
**Primary Subject Heading**:	Rheumatology
Secondary Subject Heading:	Medical management

For peer review only - http://bmjopen.bmj.com/site/about/guidelines.xhtml

Keywords:	LIFT, Fatigue, Inflammatory rheumatic disease, randomised controlled trial, Cognitive behavioural, exercise

SCHOLARONE™
Manuscripts

Protocol for a multi-centre randomised controlled parallel-group trial to compare the effectiveness of remotely delivered cognitive behavioural and graded exercise interventions with usual care alone to lessen the impact of fatigue in inflammatory rheumatic diseases (LIFT)

For peer review only

Kathryn R Martin[1,2*,**], Eva-Maria Bachmair[1,2*], Lorna Aucott[3], Emma Dures[4], Richard Emsley[5], Stuart R Gray[6], Sarah Hewlett[4], Vinod Kumar[7], Karina Lovell[8], Gary J Macfarlane[1,2], Graeme MacLennan[3], Paul McNamee[9], John Norrie[10], Lorna Paul[11], Stuart Ralston[12], Stefan Siebert[13], Alison Wearden[14], Peter D White[15], Neil Basu[13]

[*] joint first authors

[1] Epidemiology Group, University of Aberdeen, Health Science Building 1st Floor, Foresterhill, Aberdeen AB25 2ZD, UK; [2] Aberdeen Centre for Arthritis and Musculoskeletal Health, University of Aberdeen, Foresterhill, Aberdeen AB25 2ZD, UK; Email: e.bachmair@abdn.ac.uk, g.j.macfarlane@abdn.ac.uk, kathryn.martin@abdn.ac.uk;

[3] Centre of Healthcare and Randomised Trials (CHaRT), Health Service Research Unit, University of Aberdeen, Health Science Building 3rd Floor, Foresterhill, Aberdeen AB25 2ZD, UK; Email: g.maclennan@abdn.ac.uk, l.aucott@abdn.ac.uk

[4] Department of Nursing and Midwifery University of the West of England, Academic Rheumatology Unit, Bristol Royal Infirmary, Bristol BS2 8HW, UK; Email: Emma2.dures@uwe.ac.uk; Sarah.hewlett@uwe.ac.uk

[5] Department of Biostatistics and Health Informatics, Institute of Psychiatry, Psychology and Neuroscience, King’s College London, De Crespigny Park,

Denmark Hill, London SE5 8AF, UK; Email: Richard.Emsley@kcl.ac.uk

[6] Institute of Cardiovascular and Medical Sciences, BHF Glasgow Cardiovascular Research Centre, University of Glasgow, Glasgow G12 8TA, UK; Email: stuart.gray@glasgow.ac.uk

[7] Ninewells Hospital, NHS Tayside, Dundee DD1 9SY, UK; Email: Kumar.vinod@nhs.net

[8] School of Health Sciences, University of Manchester, Jean McFarlane Building, University of Manchester, Oxford Road, Manchester M13 9PL, UK; Email: karina.lovell@manchester.ac.uk

[9] Health Economics Research Unit, University of Aberdeen, Polwarth Building, Foresterhill, Aberdeen AB25 2ZD, UK; Email: p.mcnamee@abdn.ac.uk

[10] Edinburgh Clinical Trials Unit , University of Edinburgh, Outpatients Building, Floor 2, Western General Hospital, Crewe Road South Edinburgh EH4 2XU, UK; Email: j.norrie@ed.ac.uk

[11] School of Health and Life Science, Glasgow Caledonian University, Cowcaddens Road, Glasgow G4 0BA, UK; Email: lorna.paul@gcu.ac.uk

[12] Rheumatology and Bone Disease, University of Edinburgh, Western General Hospital, Edinburgh EH4 2XU, UK; Email: stuart.ralston@ed.ac.uk

[13] Institute of Infection, Immunity and Inflammation, University of Glasgow, 120 University Place, Glasgow G12 8TA, UK; Email: stefan.siebert@glasgow.ac.uk; neil.basu@glasgow.ac.uk

[14] School of Health Sciences, University of Manchester, Coupland 1 Building, Oxford Road, Manchester, United Kingdom M13 9PL, UK; Email: alison.wearden@manchester.ac.uk

For peer review only - http://bmjopen.bmj.com/site/about/guidelines.xhtml

For peer review only

[15] Department of Psychological Medicine, Queen Mary University of London, Bartholomew's Hospital, William Harvey House, West Smithfield, London EC1A 7BE, UK; Email: peter.white6@qmul.ac.uk

**Corresponding author: Dr Kathryn R Martin, Epidemiology Group, University of Aberdeen, Health Science Building 1st Floor, Foresterhill, Aberdeen AB25 2ZD, United Kingdom; Email: kathryn.martin@abdn.ac.uk; Telephone: +44 (0) 1224 437117

Word count: 6295

For peer review only

For peer review only - http://bmjopen.bmj.com/site/about/guidelines.xhtml

ABSTRACT

Introduction: Fatigue remains pervasive, disabling and challenging to manage across all inflammatory rheumatic diseases (IRDs). Non-pharmacological interventions, specifically cognitive behavioural approaches and graded exercise programmes designed to support and increase exercise, are valuable treatments which help IRD patients manage their fatigue. Yet, health care systems have encountered substantial barriers to the implementation of these therapeutic options. LIFT is designed to give insights into the effectiveness of a remotely delivered standardised intervention for a range of IRD patients. It will also enable the exploration of putative moderating factors which may allow for the future triage of patients and to investigate the precise mediators of treatment effect in IRD-related fatigue.

Methods and Analysis: LIFT (Lessening the Impact of Fatigue in inflammatory rheumatic diseases: a randomised Trial) is a pragmatic, multi-centre, three-arm randomised, controlled trial, which will test whether adapted cognitive behavioural approach (CBA) and personalised exercise programme (PEP) interventions can individually reduce the impact and severity of fatigue. This will be conducted with up to 375 eligible patients diagnosed with IRD and interventions will be delivered by rheumatology health care professionals, using telephone or internet-based audio/video calls.

Ethics approval and dissemination: Ethical approval has been granted by Wales REC 7 (17/WA/0065). Results of this study will be disseminated through presentation at scientific conferences and in scientific journal. A lay summary of the results will be sent to participants.

For peer review only - http://bmjopen.bmj.com/site/about/guidelines.xhtml

Strengths and limitations of this study

- LIFT is designed to give insights into the effectiveness of a remotely delivered standardised intervention for a range of IRD patients.
- It will also enable the exploration of putative moderating factors which may allow for the future triage of patients and to investigate the precise mediators of treatment effect in IRD-related fatigue.
- Although participants and therapist are not blinded to treatment allocation, all research staff at study sites performing assessments are blinded.
- The availability of rheumatology healthcare professionals to be trained as therapists for the study may be restricted.
- Patients responding to study invitation may not represent the entire population, however we seek to mitigate this by considering all patients, with a relevant diagnosis, managed at each recruiting hospital.

Trial registration: ClinicalTrials.gov ID NCT03248518, registered on 14 August 2017

Keywords: LIFT, fatigue, inflammatory rheumatic disease, randomised controlled trial, cognitive behavioural, exercise, telephone, telehealth

For peer review only

INTRODUCTION

Despite major advances in the management of inflammatory rheumatic diseases (IRDs), patients remain burdened by their disease and cite fatigue as a principal problem. In rheumatoid arthritis (RA), for example, as many as 80% of patients report significant fatigue [1] and over 70% consider fatigue to be equal to pain in terms of burden [2]. Moreover, fatigue is a crucial determinant of impaired quality of life [3, 4] and a predictor of work disability [5, 6]. Indeed, over 75% of patients identify fatigue as the main barrier to remaining in employment [7]. Studies in other chronic IRDs, such as axial spondyloarthritis (AxSpA) and systemic lupus erythematosus (SLE), have reported similar fatigue prevalence of 66-85% [8, 9] and report the impact of fatigue on quality of life and employment to be equally pronounced [10-12]. In spite of these profound consequences, patients feel this symptom is clinically neglected [13, 14] and rheumatologists admit uncertainty regarding its management [15].

This situation reflects the poor availability of suitable interventions within traditional health care systems such as the NHS, but this is not to say that effective treatments do not exist. There is now considerable consensus across the health care community that non-pharmacological interventions, specifically cognitive behavioural approaches (CBA) and programmes designed to support increased exercise, are valuable treatments which help IRD patients manage the fatigue associated with their chronic diseases [16, 17].

Our current team has made key contributions to the evidence base, which supports the use of these treatments for fatigue in IRDs [18-20]. However, current health care systems pose to substantial barriers to the implementation of these therapeutic options as part of standard clinical care.

For peer review only - http://bmjopen.bmj.com/site/about/guidelines.xhtml

Firstly, existing studies – including our own – have only developed bespoke disease-specific models of care, which vary in content, structure and method of delivery. Inevitably, this necessitates the development of multiple particular skill sets and duplicated pathways for the care providers if they are to equitably serve their diverse patient populations – a time consuming, costly and inefficient undertaking.

Secondly, patients find it challenging to commit to regular face-to-face treatment sessions (a common underpinning of existing CBA and exercise interventions). This is often due to a combination of health complications and the time-constrained nature of modern life, particularly relevant to those patients still in employment.

Thirdly, individual patients report substantial variation in their preference and response to the distinct interventions of CBA and exercise [21, 22].

Rationale for Study

It has becoming increasingly clear that:

a) similarities exist across chronic IRDs regarding the nature and likely mechanisms which maintain fatigue – such as dysfunctional activity behaviours [23-25] and illness beliefs [14, 26, 27],- and so the application of standardised generic, rather than disease-specific, interventions may prove effective;

b) alternative, more flexible, methods of remote delivery such as telephone and internet-based audio/video calls can be just as effective as traditional face-to-face interventions [28, 29]; and

c) in the future, the identification of baseline patient preferences and characteristics which can predict differential treatment effects (moderators) will be vital to inform a personalised triage approach to care [22, 30].

For peer review only - http://bmjopen.bmj.com/site/about/guidelines.xhtml

Therefore, a pragmatic study is proposed, which will use CBA and personalised exercise programme (PEP) interventions, informed by previously developed interventions [31, 32] which will be generically applicable across IRD-fatigue populations. We will test whether these two key non-pharmacological interventions compared with usual care alone can individually reduce fatigue, when delivered by the rheumatology team, across a mixture of IRDs using telephone or internet-based audio/video calls. In doing so, this will enable us to explore potential moderating factors which may allow for the future triage of patients according to the most suitable intervention, as well as to investigate the precise mediators of the effect of treatment on IRD-related fatigue.

Objectives

Primarily, we seek to test the hypothesis that either standardised CBA or PEP interventions, in addition to usual care, is more effective than usual care alone to lessen the impact and severity of fatigue. We will also explore the underlying moderators (in order to inform future patient triage) and mediators (in order to optimise future interventions) of IRD-related fatigue.

For peer review only - http://bmjopen.bmj.com/site/about/guidelines.xhtml

METHODS AND ANALYSIS

Study description

LIFT is a multi-centre, three-arm randomised controlled trial testing usual care with additional adapted CBA or PEP interventions versus usual care alone. Eligible participants will be identified from approximately 3600 patients with IRD attending major secondary care rheumatology services in the UK (Figure 1). Recruitment for the study began in August 2017. We anticipate recruiting 375 participants previously diagnosed with IRD.

The timeline for assessment and delivery of interventions is summarised in Figure 2. We anticipate that active CBA and PEP interventions will start between 2 and 8 weeks post-randomisation, with an average delay of 4 weeks after randomisation. Follow-up data will be collected from all participants at 10 weeks, 28 weeks and 56 weeks after randomisation adjusted for the average delay of 4 weeks, therefore all participants will remain part of the study for 13 months.

Participant Recruitment and Eligibility

Enrolment is by invitation only and all potential participants will undergo a two-step screening process consisting of a pre-study invite and an assessment of their hospital medical notes. Patients with rheumatologist diagnosed IRDs (e.g. RA, SLE and AxSpA, psoriatic arthritis, vasculitis or Sjogren's Syndrome) will receive a mailed pre-study invite consisting of a cover letter (see Supplementary material, Additional File 1 for details on Study within a Trial), study information and questions to explore interest and eligibility.

For peer review only - http://bmjopen.bmj.com/site/about/guidelines.xhtml

After return of the required pre-study screening questions, the research personnel at each site will assess their medical notes to determine eligibility based on the inclusion and exclusion criteria (Table 1).

Table 1 Inclusion and exclusion criteria

In order to be considered eligible for participation in the study they must:	
Criterion	Characteristics of eligible participants
1.	Be male or female aged ≥ 18 years at the time of consent
2.	Have been diagnosed with an IRD such as RA, SLE or AxSpA by a consultant rheumatologist
3.	Report fatigue to be a persistent problem as evidenced by answering both questions:<br>1. Have you had problems with fatigue for more than three months? (Yes)<br>2. Please circle the number that shows your average level of fatigue during the past 7 days. (≥ 6 based on a numerical rating scale of 0 (no fatigue) to 10 (totally exhausted))
4.	Have access to a telephone landline or mobile telephone and/or internet based audio/video calls
5.	Give permission for researchers to access their hospital medical notes

For peer review only - http://bmjopen.bmj.com/site/about/guidelines.xhtml

6.	Have stable disease as evidenced by no change in immunomodulatory therapy within the last three months based on hospital medical records
7.	Currently be under the care of a secondary care physician
Participants will be excluded if:	
Criterion	Characteristics of ineligible participants
1.	there are significant abnormalities in thyroid function (TSH levels) in the most recent blood test done within the last three months
2.	there is evidence of severe anaemia (haemoglobin levels) in the most recent blood test done within the last three months
3.	there is evidence of severe renal dysfunction (eGFR) in the most recent blood test done within the last three months
4.	they have a medical condition which would make the proposed interventions unsuitable, e.g. significant heart disease
5.	they are pregnant
6.	they are unable to understand English sufficiently to take part in the intervention
7.	they are unable to provide written informed consent
8.	they are not willing to be randomised
9.	they are currently participating in an interventional clinical trial

Abbreviations: AxSpA, Axial Spondyloarthritis; eGFR, estimated glomerular filtration rate; RA, rheumatoid arthritis; SLE, Systemic Lupus Erythematosus, TSH, Thyroid Stimulating Hormone

For peer review only - http://bmjopen.bmj.com/site/about/guidelines.xhtml

A trained rheumatology research nurse will review and confirm eligibility. In addition, the local rheumatology consultant will have the opportunity to withdraw or exclude participants. If a participant has been identified as potentially eligible s/he will be invited to an appointment for a baseline assessment visit at the local study site.

Consenting participants

Patients will make a final decision to participate when they attend the local study site for the baseline assessment visit. No study specific procedures will take place before written consent has been obtained. At the baseline assessment visit, a designated member of the local research team will confirm eligibility (including re-confirmation of fatigue state). It will also be determined whether the participant has ever met the relevant classification criteria for RA (mandatory, 2010 ACR/EULAR [33] or 1987 ACR [34]), or other IRDs (non-mandatory; SLE 1997 ACR SLE criteria [35], ASAS criteria for AxSpA [36]).

Randomisation

Participants will be allocated to receive either of the two treatments or usual care alone (1:1:1 ratio) using a computer generated sequence. Randomisation will be minimised by diagnosis (RA, SLE, or AxSpA or other IRD) and the presence/absence of depressive symptoms (Hospital Anxiety & Depression Scale (HADS) [37] subscale score >10) and will include a random element set at 20%.

Blinding

Full blinding will not be possible due to the need for participants to engage in specific behavioural change interventions. However, to reduce detection bias, we will aim to

For peer review only - http://bmjopen.bmj.com/site/about/guidelines.xhtml

"blind" research personnel undertaking outcome assessments to participants' treatment allocation. To facilitate blinding we will remind participants to refrain from discussing (and subsequently revealing) their treatment allocation at follow-up assessments with research personnel. Finally, all data will be analysed blind to allocation.

Interventions and Treatment protocol

Usual Care

All participants will receive usual care and will receive a Versus Arthritis (formerly Arthritis Research UK) education booklet for self-management of fatigue [38] by post. It represents usual care in almost all UK rheumatology centres and is freely available. The booklet covers the major relevant topics (including fatigue validation, energy management, priorities, sleep, stress, and assertiveness) underpinned by goal-setting and self-monitoring of activity. It encourages, at several key points that the patient asks their rheumatology team for support to work through the booklet. We will not restrict what usual care may involve, but will monitor the care received for all participants as part of our health economics analysis.

Participants randomised to the active treatments will also receive either CBA or PEP. The CBA and PEP treatments are adapted from previous fatigue specific cognitive behavioural [31, 32] and exercise interventions [31] to ensure that they are suitable for a remote delivery via telephone or internet-based audio/video call and applicable to the broad spectrum of IRD.

For peer review only - http://bmjopen.bmj.com/site/about/guidelines.xhtml

Cognitive Behavioural Approach (CBA)

CBA is a structured psychological intervention, which explicitly aims to replace unhelpful beliefs and behaviours with more adaptive ones. In this study, the CBA will target a number of unhelpful behavioural patterns such as ‘activity avoidance’ and ‘all or nothing’. These can lead to negative mood states, which exacerbates fatigue even further. Following a brief assessment of individual beliefs and behaviours surrounding fatigue, the aim of the treatment is to change unhelpful beliefs and behavioural factors through the application of participant-centred strategies and behavioural activities, which are supported by written materials and regular consultations with rheumatology health care professionals. The participants will receive additional leaflets and diaries to assist them with making changes to manage fatigue.

Personalised Exercise Programme (PEP)

PEP is based on the premise that chronic fatigue relates to exercise intolerance, supported by unhelpful illness beliefs (such as fear avoidance) and deconditioning, with a consequent increase of effort (perceived or otherwise). PEP aims to disrupt this vicious cycle by a graded exposure behaviour therapy, which is symptom contingent, to gradually optimise patients’ levels of exercise with a view to modifying their altered perception of effort, improve their tolerance of exercise, fitness and function, reverse the deconditioning and ultimately reduce the severity and impact of fatigue. In this study, participants will receive an individually tailored graded exercise programme, delivered according to their physical capacity and gradually increased in duration and then intensity. The participants will receive additional information and diaries to assist with the intervention. The intervention will use pedometers and/or

For peer review only - http://bmjopen.bmj.com/site/about/guidelines.xhtml

heart rate monitors to enhance motivation. Overall, the times and duration of exercise will be recorded in exercise diaries during the intervention.

Both CBA and PEP interventions will be delivered by health care professionals. PEP may be delivered by a rheumatology specialist physiotherapist and CBA may be delivered by a rheumatology nurse or an equally qualified and trained allied health professional (e.g. occupational therapist) who are members of local NHS staff. Participants will be offered seven one-to-one telephone or internet-based audio/video call (based on patient preference) sessions (up to 45 minutes) of CBA or PEP interventions over a period of 14 weeks with a trained therapist (Figure 2). The first session of PEP, however, will be delivered face-to-face. A booster session will be conducted at 22 weeks after the start of therapy by the relevant health care professional.

Training of health care professionals as therapists before the study

Separate CBA and PEP training will be provided for the NHS staff delivering the interventions. This will comprise an intensive 2-day group course delivered by experienced designated investigators supplemented with the therapist manuals. The course will use a range of methods including skills practice with specific feedback using fictitious but typical fatigued cases.

Supervision and support of therapists during the study

Supervision will be provided by designated investigators on a fortnightly basis, or as required, to the therapists either face-to-face or by telephone depending on feasibility and preference. In addition, support will be available in cases where a therapist

For peer review only

requires assistance with respect to a particular participant. In addition to the option to contact the supervisor directly, we will have a notification system incorporated in the database.

Treatment fidelity

Some of the intervention sessions will be recorded and used in supervision to provide feedback to therapists and to ensure treatment fidelity. We aim to take a 5% sample of intervention sessions for those in CBA and PEP from participants who agree to be recorded which is based on a random sample generated from an algorithm that takes into account session number, therapist, site location, patient gender. This is equivalent to approximately 89 recordings per intervention and will be subject to treatment adherence (no sessions completed) and participant permission to record sessions.

Outcome measures

All outcome and mediator measures will be assessed at randomisation (baseline) and then, on average, 10 weeks, 28 weeks and 56 weeks thereafter (by questionnaire unless stated) in all participants (Figure 3). The outcome measures at each time point and their source (i.e. questionnaire, medical record, blood sample, diary) are summarised in a study matrix (Supplementary material Table 1).

Primary outcome measures

The primary outcome, fatigue, is measured with the Chalder Fatigue Scale (CF) [39] which assesses the physical and mental symptoms of fatigue using Likert scoring, and the Fatigue Severity Scale (FSS) [40] assessing the impact of fatigue at 56

For peer review only - http://bmjopen.bmj.com/site/about/guidelines.xhtml

weeks post randomisation. If the effect of the intervention is positive on the CF, then the FSS outcome will be formally analysed. Should the intervention have no effect on the CF, then an explorative analysis of the FSS data will be performed.

Secondary outcome measures

Secondary outcomes are:

Fatigue: Bristol Rheumatoid Arthritis Fatigue Multi-Dimensional Questionnaire [41] (BRAF-MDQ) assessing the physical, living, cognition and emotional aspects of fatigue

Quality of life & health utility index: Short Form-12 [42] assessing functional health and wellbeing from the participant's perspective

Pain: Pain numerical rating scale [43] assessing pain intensity

Anxiety and depression: Hospital anxiety and depression scale (HADS) [37]

Sleep: Sleep problem scale [44]

Impact on work: Work Productivity and Activity Impairment Questionnaire: Specific Health Problem (WPAI:SHP) [45]

Impact on activities: Valued Life Activities Scale (short form S-VLA) [46]

Global outcome: Change of global health

Mediator/moderator measures

While many secondary outcome measures may also function as mediators, or their baseline values as moderators, more detailed cognitive, behavioural, clinical and physical data will be collected in order to fully characterise these factors.

Cognitions and behaviours: Brief Illness Perception Questionnaire [47], Behavioural Response to Illness Questionnaire [48].

For peer review only - http://bmjopen.bmj.com/site/about/guidelines.xhtml

Clinical: Presence of fibromyalgia [49], serological status, erosive status, disease duration, previous and current pharmacological therapies, disease activity: self-reported using a numeric rating scale, DAS28 for RA (mandatory), other disease specific activity measures (non-mandatory), inflammation (CRP/ESR), presence of other co-morbidities (Charlson Comorbidity Index) [50].

Physical: Physical activity and sedentary behaviour profiles will be measured by an activity monitor (*activ*PAL, Paltechnologies Ltd, Glasgow). The *activ*PAL will be fitted to the participant at each assessment visit and participants will be instructed to remove the device and post it back to the research team after seven days in the stamped addressed envelope provided to them.

Quantifying aerobic fitness: A step test, which involves participants wearing a heart rate monitor and stepping onto a 10 inch high box for three minutes at different stepping rates will assess aerobic fitness. Participants stop the test if their heart reaches 65% of predicted maximal heart rate (220-age or 190-age if participant is prescribed beta-blocker) at the end of any stage. One minute of rest is given between stages and maximal oxygen uptake is estimated from heart rate recordings according to established equations [51]. In addition, values of Borg Rating of Perceived Exertion (RPE) are collected.

Neuroimaging data: Participants will have an option to undertake a multi-modal magnetic resonance imaging (MRI) brain scan (see Supplementary material Additional file 2)

Furthermore, participants will be given the option to provide additional blood samples which will be stored in a designated freezer at the University of Aberdeen. These will be a maximum of 3 tubes per visit of 1x PAXgene DNA (visit 1 only), 1x PAXgene

For peer review only - http://bmjopen.bmj.com/site/about/guidelines.xhtml

RNA (visit 1, 2, 3), 1x serum (visit 1, 2, 3). Additional consent for their use in future unspecified studies will be obtained.

Quantitative process evaluation

Participant preference: Participants will be given a short synopsis of all three treatments, usual care, CBA and PEP interventions as treatment for IRD-related fatigue at baseline. They will then be asked about which treatment they would choose if they had a choice, as well as about their strength of preference.

Participant adherence: Adherence to the interventions will be monitored via attendance records kept by the therapists. In addition, participants receiving the CBA or PEP interventions will be contacted by a member of Trial Office in Aberdeen at time of session 4 (approximately week 8) and session 8 (approximately week 26) for a telephone interview. They will be asked to indicate on a scale from 0 (not at all) to 10 (completely), if they think that this treatment is the right approach and their willingness to engage and adhere to the intervention. At the same time, the therapists will be asked during supervision to what extent they think that the participant has engaged with treatment and adhered to the agreed actions and plans.

Intervention acceptance: Acceptance by participants will be evaluated in all three treatment arms using the Client Satisfaction Questionnaire [52] at 28 weeks.

Qualitative evaluation

A subgroup of participants will be invited to take part in a nested qualitative evaluation study. We will conduct qualitative evaluations of both participants who received CBA or PEP after they completed the 56 week follow-up and all therapists who will deliver the interventions. To ensure integrity, participants and therapists will

For peer review only - http://bmjopen.bmj.com/site/about/guidelines.xhtml

be invited to take part once they have left or completed the study. Data will be collected through semi-structured interviews conducted by telephone or by internet-based audio/video calls. In addition to the practical considerations of offering options, this approach acknowledges that both participants and therapists will have been involved in a remotely delivered intervention. All interviews will be audio recorded, anonymised during transcription, and checked for accuracy.

Participant experiences of the interventions will be analysed using a framework analysis [53] to assess the content, mode of delivery, acceptability, barriers and facilitators, helpfulness and subsequent impact of the interventions on their daily lives. We will use a maximum variation sampling strategy [54] to include participants with a range of IRDs, gender, age, disease duration and primary outcomes in the randomised controlled trial. To achieve this, a minimum of 40 interviews is required. All selected participants will receive additional information after they have completed the last assessment visit (week 56) and provide separate written informed consent for the qualitative component.

All therapists will be invited to take part in a telephone interview to explore experiences of intervention training and delivery, including challenges and benefits of learning and using new skills, and barriers and facilitators to supporting patients remotely using a structured manual. This evaluation will be analysed using inductive thematic analysis [55]. All therapists will receive additional information after they have either left the study or after the intervention phase and provide separate written informed consent for the qualitative component.

For peer review only

Patient safety

There are unlikely to be major safety issues with our proposed non-pharmacological interventions. However, if the therapist delivering CBA and PEP or any study personnel have any safety concerns, we will follow a specific Standard Operating Procedure for adverse events in non-CTIMP studies.

Laboratory and sample analysis

This multi-centre study involves a number of standard blood investigations, i.e. TSH, haemoglobin, serum creatinine, CRP and ESR, which are routinely processed by local NHS laboratories. Blood samples for CRP and ESR analysis and sample storage for future ethically approved research will be taken at each assessment as specified in the study matrix by trained personnel only following established procedures. Blood samples for TSH, haemoglobin and serum creatinine analysis will only be taken at baseline if they are required to confirm eligibility. Sample coding, preparation, storage, analysis and transfer of results and optional blood samples for long-term storage at the trial centre will be performed according to the analytical protocol based on national laboratory guidelines. The exact logistic and procedure will be agreed with each site before the start of the study and every effort will be made to standardise the workflow across sites to reduce bias. Additional optional blood samples will be stored for future ethically approved research. All study blood results (abnormal or otherwise) will also be directed towards the local PI who can determine locally how these will be handled.

For peer review only - http://bmjopen.bmj.com/site/about/guidelines.xhtml

Withdrawal Procedures

Participants will have the option to withdraw at any time during the study period of 13 months. The participant needs to request this formally and a withdrawal document will be completed and signed by the designated research staff at the local sites. They have the option to either withdraw from the study completely or from parts of it (pre-study invite, treatment or follow-up). If participants withdraw from the study completely or from follow-up assessments, they will not receive further invitations but we will use the data collected prior to the withdrawal (depending on permission). Those withdrawing from the treatment only will continue to be sent invitations to attend and complete follow-up assessment visits, unless they request to withdraw completely later on. Failure of any participant to complete a follow-up at any particular time-point will not be counted as a withdrawal unless the participant formally requests to withdraw. In addition, a participant can also be withdrawn by others, for example the local PI or primary consultant, should the need arise at any stage throughout the study period. This also includes loss of capacity for ongoing consent.

Statistical Issues

Sample size

Our planned primary intention to treat analyses will compare PEP + usual care versus usual care alone, and CBA + usual care versus usual care alone separately. We base our calculations on a standardised effect size of 0.50 (considered credible in other pragmatic effectiveness studies). This would equate to being powered to detect a minimal important clinical difference of 2 units in the CF Scale, assuming a common standard deviation across the randomised groups of 4 units. Assuming an

For peer review only

For peer review only - http://bmjopen.bmj.com/site/about/guidelines.xhtml

overall significance level of 5% (by calculating the two pre-specified randomised groups comparisons, PEP + usual care vs. usual care alone and CBA + usual care vs usual care alone, at 2.5%, to maintain an overall level of not more than 5%) and a power of 90%, we require 100 evaluable participants in each of the three groups. The data of participants are evaluable when outcomes at the 56 weeks follow-up are available. Based on our own previous studies, we estimate a dropout rate of 20% and therefore we anticipate recruiting 375 participants randomly allocating 125 into each treatment group.

Statistical analysis

All statistical analyses will be governed by a comprehensive Statistical Analysis Plan, which will be authored by the trial statistician and approved by the Trial Steering Committee and the Data Monitoring Committee before the main study outcome data are examined. All analyses will be carried out using standard statistical software. In accordance with CONSORT guidelines [56], we will report all participant flow through the study. Descriptive statistics of recruitment, drop-out and completeness of interventions will be provided.

Effectiveness Analysis: The main effectiveness analysis will be via intention-to-treat including all participants, with no planned interim analysis for early termination for either overwhelming evidence of effectiveness or abandoning for futility. Baseline characteristics will be presented by randomised group without formal statistical tests. We will test the primary hypothesis for between-group change in the primary outcome for each of the two pre-specified comparisons (CBA + usual care vs usual care alone and PEP + usual care vs usual care alone) using repeated measures

For peer review only

mixed model, with subject as a random effect, and a suitably specified covariance structure (e.g. Autoregressive [1] (AR[1]), therapist as a random effect (to adjust for any clustering by therapist), with baseline outcome measure, and any other strongly predictive baseline measures, including the minimisation factors of presence/absence of depression and centre. Treatment and its interaction with time will be fitted as fixed effects, and we will apply standard regression diagnostics. The analysis will use statistical techniques for handling missing outcome data using multiple imputation under a missing at random (MAR) assumption. The secondary outcomes will be analysed using an analogous method. The main estimate of treatment effect will focus on the 56 weeks after baseline.

Mediation analysis: We will use modern causal inference methods to investigate the set of mediator measures. If the effectiveness analysis shows significance between group differences in the mediators, then we will use parametric regression models to test for effect of mediator on outcome, and the residual direct effect of treatment on outcome. Since all the measures are continuous, the indirect effects will be calculated by multiplying relevant pathways and bootstrapping will be used to produce valid standard errors for the indirect effects. All analyses will adjust for baseline measures of the mediators, outcome and putative measured confounders. Mediation analyses are potentially biased by measurement error in mediators and hidden confounding between mediators and outcomes; we will build on our previous methodological and applied work in this context to include repeated measurement of mediators and outcomes to account for classical measurement error and baseline confounding.

For peer review only

Moderation analysis: We will examine differential treatment effects using the set of moderator measures by extending the intention to treat analysis models to include an interaction term between intervention and each of the moderators separately. We will use bias correction/cross-validation methods to identify robust evidence for individual moderation and for a moderation index, both on the overall effect and also along the steps of the mediation pathway.

Every effort will be made to ensure data collection is complete. However, we will use statistical techniques for handling missing outcome data. Multiple imputation under a MAR assumption will be used in the first instance, with additional sensitivity analysis if the MAR assumption is not satisfied.

Health Economics evaluation

An economic evaluation will be conducted from both a health care system and societal perspective. Participants will be asked to record, in a diary, all types and duration of hospital admissions, frequency of visits to hospital for outpatient attendances, and other visits to or from relevant health professionals (e.g. general practitioners, nurse practitioners, physiotherapists) and specify whether the main reason for the visit was fatigue. Each participant will be asked to keep the diaries between the baseline and third assessment visit (approximately 28 weeks). Furthermore they will be asked to keep diaries for two weeks after the third assessment visit and two weeks before they return for the last assessment visit. National sources of unit cost data will be applied to value resource use (HRG Reference Costs, Unit Costs of Health and Social Care). The costs associated with

For peer review only - http://bmjopen.bmj.com/site/about/guidelines.xhtml

delivery of the interventions will be estimated using records kept by therapists of the number and duration of calls per participant.

Participants will be asked to report any contacts with private practitioners, and the costs of over the counter medication/complementary therapies purchased. This also includes additional expenses related to their condition or fatigue as well as if there was an impact on paid and unpaid work.

Health-related quality of life data will be collected using the SF-12 and these data will be converted to quality of life weights using published tariffs. These data will then be used to calculate Quality Adjusted Life Years (QALYs). As the intervention may affect general well-being as well as reduce fatigue, we will also collect values for changes in well-being data using the ICECAP instrument [57] and changes in life satisfaction [58].

Study management and conduct

The study will be co-ordinated by a Trial Management Group, consisting of the grant holder (CI), additional members of the research group, a study co-ordinator and a representative from CHaRT.

A Trial Steering Committee (TSC) has been established. The TSC comprises an independent chair who has expertise in trials and other members, both independent and study investigators, with background relevant to IRD or type of interventions including a lay representative, who has lived experience of IRD related fatigue, and a clinician working with people with IRD.

An independent Data Monitoring Committee has been established to oversee study progress. The Data Monitoring Committee comprises of a chair who has expertise in

For peer review only

For peer review only - http://bmjopen.bmj.com/site/about/guidelines.xhtml

trials, a biostatistician and two other independent members with a rheumatology background.

End of Study

The end of study is defined as last data collection of either the qualitative evaluation study phase or during the last follow-up visit at 56 weeks from the last participant after CBA/PEP intervention or usual care start date – whichever comes last.

Data management, protection, storage and archiving of study documents

All investigators and study staff involved with this study will comply with the requirements of the Data Protection Act 2018 with regard to the collection, storage, processing and disclosure of personal information and will uphold the Act's core principles. The investigators and study staff will also adhere, if appropriate, to the current version of the NHS Scotland Code of Practice on Protecting Patient Confidentiality. Access to collated participant data will be restricted to the CI and appropriate study staff as needed. All laboratory specimens, evaluation forms, reports, and other records will be identified using unique participant ID numbers to maintain participant confidentiality. All records will be kept in a secure storage area with limited access to study staff only. Personal data, including postal address, phone numbers (i.e. landline and mobile), email addresses, and anonymised data files for study outcomes are stored in locked filing cabinets (hard copy) and in a bespoke database provided and maintained by CHaRT as well as secured shared drives with access via password controlled computers (university and NHS networks) by study staff only (electronic data).

For peer review only

All study documentation will be kept for a minimum of 5 years from the protocol defined end of study point in the University of Aberdeen archive. When the minimum retention period has elapsed, study documentation will not be destroyed without permission from the sponsor.

Patient and Public Involvement

Patients and their carers were involved in the development of the research question and the prioritisation of fatigue as a subject for research. We have benefitted from collaborations with representatives of the National Rheumatoid Arthritis Society, the National Ankylosing Spondylitis Society and Lupus UK who have significantly influenced the trial design. In addition, we have worked with patient advisory groups which provided valuable input into the content and layout of the information material and other documents that will be given to the participants to assess the burden of the intervention and data collection visits. Although patients were not actively involved in the recruitment, they are involved in the conduct of the study as lay representatives in the TSC. After completion of the study, the main results and conclusions of the study will be disseminated to participants in lay language in form of a newsletter.

DISCUSSION

The LIFT trial is a pragmatic multi-centre, three-arm randomised controlled trial. It has been designed to test the effectiveness and cost-effectiveness of remotely delivering two non-pharmacological interventions, CBA and PEP, over a period of 22 weeks so as to answer whether treatment can move beyond face-to-face disease-specific interventions for IRD-related fatigue. In order to achieve the study aims, the CBA and PEP interventions were informed by previous evidence-based cognitive

For peer review only - http://bmjopen.bmj.com/site/about/guidelines.xhtml

behavioural and exercise interventions [31, 32] for fatigue to ensure suitability across IRD conditions, as well as to ensure that both interventions could be delivered via telephone or internet-based audio/video calls by a range of rheumatology health care professionals trained as therapists. To reduce selection bias, all patients with a relevant diagnosis, managed at each recruiting site who report fatigue as a persistent problem will be considered, either through database screening or manual screening by research nurses, and invited to take part in the trial. Participants will be asked to provide information about fatigue, our primary outcome, at baseline and weeks 10, 28, and 56 using the CF and FSS to measure severity and impact at critical times throughout the trial. In addition, detailed information on cognitions and behaviours (e.g. illness perception), clinical features (e.g. disease activity, presence of fibromyalgia), as well as physical activity and fitness levels will allow for the identification of potential moderators and mediators of the treatment effect in this trial. Although complete blinding is not possible due to provision of an active behavioural change intervention, we aim to reduce detection bias by asking participants to refrain from revealing their treatment allocation to the research nurses undertaking their outcome assessments.

Fatigue remains a significant patient-identified priority, as well as a significant challenge to manage clinically. To provide the necessary evidence to bring about change in health services consistently offered for IRD-related fatigue across the spectrum of IRD conditions, both a quantitative and qualitative evaluation will also be undertaken within the trial. Trial findings will be used inform future triage of patients into the most suitable intervention, as well as provide evidence of which mediating factors play a role in treatment effect among patients with IRD-related fatigue.

For peer review only - http://bmjopen.bmj.com/site/about/guidelines.xhtml

Ethics approval and dissemination

The study will be conducted in accordance with the principles of good clinical practice.

The Research Ethics Committee Wales REC 7 (17/WA/0065) gave approval to the study on 20.03.2017 (protocol version 2) and approved the substantial amendment 01 on 06.07.2017 (protocol version 3), substantial amendment 02 on 05.09.2017 (protocol version 4) and substantial amendment 03 on 01.12.2017 (protocol version 5) and substantial amendment 04 on 08.03.2018, protocol version 6). Extension of recruitment for 6 months was approved and implemented as minor amendment 09 on 17.09.2018 (protocol version 7, current). Management approval from all NHS health boards was obtained as required by the ethics committee before the start of the study.

Consent will be sought for recording of interventions sessions for quality assurance and treatment fidelity. Written consent will be obtained to record interviews and to include anonymised statements in publication of the qualitative evaluation outcomes.

Ownership of the data arising from this study resides with the University of Aberdeen.

A clinical study report will be prepared in accordance with ICH authorship guidelines which will be used for dissemination of findings via publication and presentation at scientific meetings. Investigators have the right to publish orally or in writing the results of the study.

There are no plans to place study outcome data in a repository at the current time.

Supplementary material

TABLE 1 Study Matrix

ADDITIONAL FILE 1 SWAT 24

For peer review only

ADDITIONAL FILE 2 MRI SUB-STUDY

Abbreviations

AxSpA, Axial Spondyloarthritis; CBA, Cognitive Behavioural Approach; CF, Chalder Fatigue Scale; CHaRT, Centre of Healthcare Randomised Trials; CI, Chief Investigator; CRP, C-reactive protein; eGFR, Estimated glomerular filtration rate; ESR, Erythrocyte Sedimentation Rate; FSS, Fatigue Severity Scale; ICECAP, Investigating Choice Experiments for the preference of older people CAPability measures for adults; ICH, International Council for Harmonisation; IRD, Inflammatory Rheumatic Disease; LIFT, Lessening the Impact of Fatigue in inflammatory rheumatic diseases: a randomised controlled Trial; MAR, missing at random; NHS, National Health Service; PEP, Personalised Exercise Programme; PI, Principal Investigator; RA, Rheumatoid Arthritis; REC, Research Ethics Committee; SLE, Systemic Lupus Erythematosus; TSH, thyroid stimulating hormone;

For peer review only

Acknowledgements

The authors would like to thank representatives of the National Rheumatoid Arthritis Society, the National Ankylosing Spondylitis Society and Lupus UK for their contribution to the study design before the grant application. The authors would also like to thank all members of our Patient Advisory Groups in Aberdeen and Bristol for their review of study material.

Funding and sponsorship

This work was supported by Versus Arthritis (formally known as Arthritis Research UK) grant number 21175. The study is sponsored and led by the University of

Aberdeen (King's College, Aberdeen, AB24 3FX). Versus Arthritis (formerly Arthritis Research UK) (funder) and Sponsor had no input into writing the manuscript.

Author Contributions

NB conceived the study and wrote the trial grant with authors ED, RE, SRG, SH, KL, GJM, KRM, GMcL, PMcN, JN, SS, LP, AW, PDW providing substantial contributions to the design of the work (LA, GMcL, and JN planned the statistical analysis; RE planned the mediation analysis; PMcN planned the health economics analysis. ED and SH planned the qualitative evaluation analysis. SRG, LP, and SS planned the PEP intervention; NB, SH, KL, and AW planned the CBA intervention. EMB, VK, and SR were involved in logistics and data acquisition). EMB, NB, and KRM wrote the first draft of the manuscript and LA, ED, RE, SRG, SH, VK, KL, GJM, GMcL, PMcN, JN, LP, SR, SS, AW, and PDW provided specific content and edited the manuscript. All authors gave final approval for publication.

Competing interests

The authors declare no other competing interests.

Consent for publication

Not applicable

Trial status

The LIFT study began recruitment in August 2017 and will be ongoing until March 2019 (anticipated). It is expected that data collection will be completed by April 2020.

For peer review only - http://bmjopen.bmj.com/site/about/guidelines.xhtml

REFERENCES

1 Repping-Wuts H, Van Riel P, Van Achterberg T. Fatigue in patients with rheumatoid arthritis: What is known and what is needed, *Rheumatology (UK)* 2009;48:207-9.

2 van Hoogmoed D, Fransen J, Bleijenberg G, et al. Physical and psychosocial correlates of severe fatigue in rheumatoid arthritis, *Rheumatology (UK)* 2010;49:1294-302.

3 Suurmeijer TPBM, Waltz M, Moum T, et al. Quality of life profiles in the first years of rheumatoid arthritis: Results from the EURIDISS longitudinal study, *Arthritis Care Res* 2001;45:111-21.

4 Rupp I, Boshuizen HC, Jacobi CE, et al. Impact of fatigue on health-related quality of life in rheumatoid arthritis, *Arthritis Care Res* 2004;51:578-85.

5 Gignac MAM, Sutton D, Badley EM. Reexamining the arthritis-employment interface: Perceptions of arthritis-work spillover among employed adults, *Arthritis Care Res* 2006;55:233-40.

6 De Croon EM, Sluiter JK, Nijssen TF, et al. Work ability of Dutch employees with rheumatoid arthritis, *Scand J Rheumatol* 2005;34:277-83.

7 National Rheumatoid Arthritis Society. Employment and Rheumatoid Arthritis in Scotland. A national picture (RA and work). 2014.

8 Aissaoui N, Rostom S, Hakkou J, et al. Fatigue in patients with ankylosing spondylitis: Prevalence and relationships with disease-specific variables, psychological status, and sleep disturbance, *Rheumatol Int* 2012;32:2117-24.

9 Zonana-Nacach A, Roseman JM, McGwin Jr. G, et al. Systemic lupus erythematosus in three ethnic groups. VI: Factors associated with fatigue within 5 years of criteria diagnosis, *Lupus* 2000;9:101-9.

10 Wang B, Gladman DD, Urowitz MB. Fatigue in lupus is not correlated with disease activity, *J Rheumatol* 1998;25:892-5.

11 Turan Y, Duruöz MT, Bal S, et al. Assessment of fatigue in patients with ankylosing spondylitis, *Rheumatol Int* 2007;27:847-52.

12 Mau W, Listing J, Huscher D, et al. Employment across chronic inflammatory rheumatic diseases and comparison with the general population, *J Rheumatol* 2005;32:721-8.

For peer review only - http://bmjopen.bmj.com/site/about/guidelines.xhtml

13 Hewlett S, Cockshott Z, Byron M, et al. Patients' perceptions of fatigue in rheumatoid arthritis: Overwhelming, uncontrollable, ignored, *Arthritis Care Res* 2005;53:697-702.

14 Davies H, Brophy S, Dennis M, et al. Patient perspectives of managing fatigue in Ankylosing Spondylitis, and views on potential interventions: A qualitative study, *BMC Musculoskelet Disord* 2013;14(1):163.

15 Repping-Wuts H, van Riel P, van Achterberg T. Rheumatologists' knowledge, attitude and current management of fatigue in patients with rheumatoid arthritis (RA), *Clin Rheumatol* 2008;27:1549-55.

16 National Institute for Health and Care Excellence (NICE). Rheumatoid arthritis: the management of rheumatoid arthritis in adults, *Clinical Guideline* 2009.

17 Arthritis and Musculoskeletal Alliance (ARMA). Standards of Care for people with Connective Tissue Diseases. 2007.

18 Tench CM, McCarthy J, McCurdie I, et al. Fatigue in systemic lupus erythematosus: A randomized controlled trial of exercise, *Rheumatology (UK)* 2003;42:1050-4.

19 Cramp F, Hewlett S, Almeida C, et al. Non-pharmacological interventions for fatigue in rheumatoid arthritis. *The Cochrane Library* 2013.

20 Hewlett S, Ambler N, Almeida C, et al. Self-management of fatigue in rheumatoid arthritis: A randomised controlled trial of group cognitive-behavioural therapy, *Ann Rheum Dis* 2011;70:1060-7.

21 Jones E. Studying the influence of patient treatment preference on outcome in a clinical trial. 2012.

22 Johnson RE, Jones GT, Wiles NJ, et al. Active exercise, education, and cognitive behavioral therapy for persistent disabling low back pain: A randomized controlled trial, *Spine* 2007;32:1578-85.

23 Tench C, Bentley D, Vleck V, et al. Aerobic fitness, fatigue, and physical disability in systemic lupus erythematosus, *J Rheumatol* 2002;29:474-81.

24 Rongen-Van Dartel SAA, Repping-Wuts H, Van Hoogmoed D, et al. Relationship between objectively assessed physical activity and fatigue in patients with rheumatoid arthritis: Inverse correlation of activity and fatigue, *Arthritis Care Res* 2014;66:852-60.

25 Brophy S, Cooksey R, Davies H, et al. The effect of physical activity and motivation on function in ankylosing spondylitis: A cohort study, *Semin Arthritis Rheum* 2013;42:619-26.

26 Treharne GJ, Lyons AC, Hale ED, et al. Predictors of fatigue over 1 year among people with rheumatoid arthritis, *Psychol Health Med* 2008;13:494-504.

For peer review only - http://bmjopen.bmj.com/site/about/guidelines.xhtml

27 Burgos PI, Alarcón GS, McGwin Jr. G, et al. Disease activity and damage are not associated with increased levels of fatigue in systemic lupus erythematosus patients from a multiethnic cohort: LXVII, *Arthritis Care Res* 2009;61:1179-86.

28 McBeth J, Prescott G, Scotland G, et al. Cognitive behavior therapy, exercise, or both for treating chronic widespread pain, *Arch Intern Med* 2012;172:48-57.

29 Mohr DC, Vella L, Hart S, et al. The effect of telephone-administered psychotherapy on symptoms of depression and attrition: A meta-analysis, *Clin Psychol Sci Pract* 2008;15:243-53.

30 Druce KL, Jones GT, Macfarlane GJ, et al. Patients receiving anti-TNF therapies experience clinically important improvements in RA-related fatigue: results from the British Society for Rheumatology Biologics Register for Rheumatoid Arthritis, *Rheumatology (Oxford)* 2015;54:964-71.

31 White PD, Goldsmith K, Johnson AL, et al. Comparison of adaptive pacing therapy, cognitive behaviour therapy, graded exercise therapy, and specialist medical care for chronic fatigue syndrome (PACE): A randomised trial, *Lancet* 2011;377:823-36.

32 Hewlett S, Ambler N, Almeida C, et al. Protocol for a randomised controlled trial for Reducing Arthritis Fatigue by clinical Teams (RAFT) using cognitive-behavioural approaches, *BMJ Open* Aug 1;5(8):e009061.

33 Aletaha D, Neogi T, Silman AJ, et al. 2010 Rheumatoid arthritis classification criteria: An American College of Rheumatology/European League Against Rheumatism collaborative initiative, *Arthritis Rheum* 2010;62:2569-81.

34 Arnett FC, Edworthy SM, Bloch DA, et al. The american rheumatism association 1987 revised criteria for the classification of rheumatoid arthritis, *Arthritis Rheum* 1988;31:315-24.

35 Hochberg MC. Updating the American College of Rheumatology revised criteria for the classification of systemic lupus erythematosus. *Arthritis Rheum* 1997;40:1725.

36 Rudwaleit M, Landewé R, Van Der Heijde D, et al. The development of Assessment of SpondyloArthritis international Society classification criteria for axial spondyloarthritis (part I): Classification of paper patients by expert opinion including uncertainty appraisal, *Ann Rheum Dis* 2009;68:770-6.

37 Zigmond AS, Snaith RP. The hospital anxiety and depression scale, *Acta Psychiatr Scand* 1983;67:361-70.

38 Arthritis Research UK. Fatigue and Arthritis - Self-help and daily living. *2269/FATIG/14-1* 2014:1-32.

39 Cella M, Chalder T. Measuring fatigue in clinical and community settings, *J Psychosom Res* 2010;69:17-22.

For peer review only

For peer review only - http://bmjopen.bmj.com/site/about/guidelines.xhtml

40 Krupp LB, Larocca NG, Muir Nash J, et al. The fatigue severity scale: Application to patients with multiple sclerosis and systemic lupus erythematosus, *Arch Neurol* 1989;46:1121-3.

41 Nicklin J, Cramp F, Kirwan J, et al. Measuring fatigue in rheumatoid arthritis: a cross-sectional study to evaluate the Bristol Rheumatoid Arthritis Fatigue Multi-Dimensional questionnaire, visual analog scales, and numerical rating scales. *Arthritis Care Res (Hoboken)* 2010;62:1559-68.

42 Ware Jr. JE, Kosinski M, Keller SD. A 12-Item Short-Form Health Survey: Construction of Scales and Preliminary Tests of Reliability and Validity, *Med Care* 1996;34:220-33.

43 McCaffery M, Beebe A. Pain: Clinical manual for nursing practice. *Pain: Clinical Manual for Nursing Practice* 1989:1-353.

44 Jenkins CD, Stanton B, Niemcryk SJ, et al. A scale for the estimation of sleep problems in clinical research, *J Clin Epidemiol* 1988;41:313-21.

45 Reilly MC, Zbrozek AS, Dukes EM. The Validity and Reproducibility of a Work Productivity and Activity Impairment Instrument, *Pharmacoeconomics* 1993;4:353-65.

46 Katz PP, Radvanski DC, Allen D, et al. Development and validation of a short form of the valued life activities disability questionnaire for rheumatoid arthritis, *Arthritis Care Res* 2011;63:1664-71.

47 Broadbent E, Petrie KJ, Main J, et al. The Brief Illness Perception Questionnaire, *J Psychosom Res* 2006;60:631-7.

48 Skerrett TN, Moss-Morris R. Fatigue and social impairment in multiple sclerosis: The role of patients' cognitive and behavioral responses to their symptoms, *J Psychosom Res* 2006;61:587-93.

49 Wolfe F, Clauw DJ, Fitzcharles M, et al. Fibromyalgia criteria and severity scales for clinical and epidemiological studies: A modification of the ACR preliminary diagnostic criteria for fibromyalgia, *J Rheumatol* 2011;38:1113-22.

50 Charlson ME, Pompei P, Ales KL, et al. A new method of classifying prognostic comorbidity in longitudinal studies: development and validation, *J Chronic Dis* 1987;40:373-83.

51 Siconolfi SF, Garber CE, Lasater TM, et al. A simple, valid step test for estimating maximal oxygen uptake in epidemiologic studies, *Am J Epidemiol* 1985;121:382-90.

52 Larsen DL, Attkisson CC, Hargreaves WA, et al. Assessment of client/patient satisfaction: Development of a general scale, *Eval Program Plann* 1979;2:197-207.

53 Gale NK, Heath G, Cameron E, et al. Using the framework method for the analysis of qualitative data in multi-disciplinary health research, *BMC Med Res Methodol* 2013;13(1):117.

For peer review only

54 Patton MQ. Qualitative research & evaluation methods. Thousand Oaks, California: Sage Publications 2002.

55 Braun V, Clarke V. Using thematic analysis in psychology, *Qual Res Psychol* 2006;3:77-101.

56 Schulz KF, Altman DG, Moher D, et al. CONSORT 2010 statement: updated guidelines for reporting parallel group randomised trials, *BMJ* 2010;340:c332.

57 Al-Janabi H, Flynn TN, Coast J. Development of a self-report measure of capability wellbeing for adults: The ICECAP-A, *Qual Life Res* 2012;21:167-76.

58 McNamee P, Mendolia S. The effect of chronic pain on life satisfaction: Evidence from Australian data, *Soc Sci Med* 2014;121:65-73.

For peer review only

For peer review only - http://bmjopen.bmj.com/site/about/guidelines.xhtml

Figure 1 Study flowchart

Patients diagnosed with IRD will be invited by post and asked to return the pre-study screening questions. Screening is a two-step process using screening questions and review of medical records to establish fatigue state and identify other exclusion criteria. Potential participants will be contacted by phone to verify eligibility criteria and invited to the baseline assessment visit. After obtaining informed consent, their eligibility is confirmed (including fatigue state as well as determination of TSH, Hb and eGFR values if these were not available in the medical notes), baseline data are collected and participants are randomised into the study.

At baseline, participants are also given a participant information sheet about the MRI sub-study. Randomised participants will be contacted by the Trial Office in Aberdeen if they are also eligible to take part in the MRI sub-study.

CBA, cognitive behavioural approach; eGFR, estimated glomerular filtration rate; Hb, haemoglobin; IRD, inflammatory rheumatic disease; PEP, personalised exercise programme; TSH, thyroid stimulating hormone; UC, usual care

Figure 2 Timeline intervention and assessment

Figure 3 Standard Protocol Items Recommendations for Interventional Trials (SPIRIT) Schedule of enrolment, interventions and assessments

For peer review only - http://bmjopen.bmj.com/site/about/guidelines.xhtml

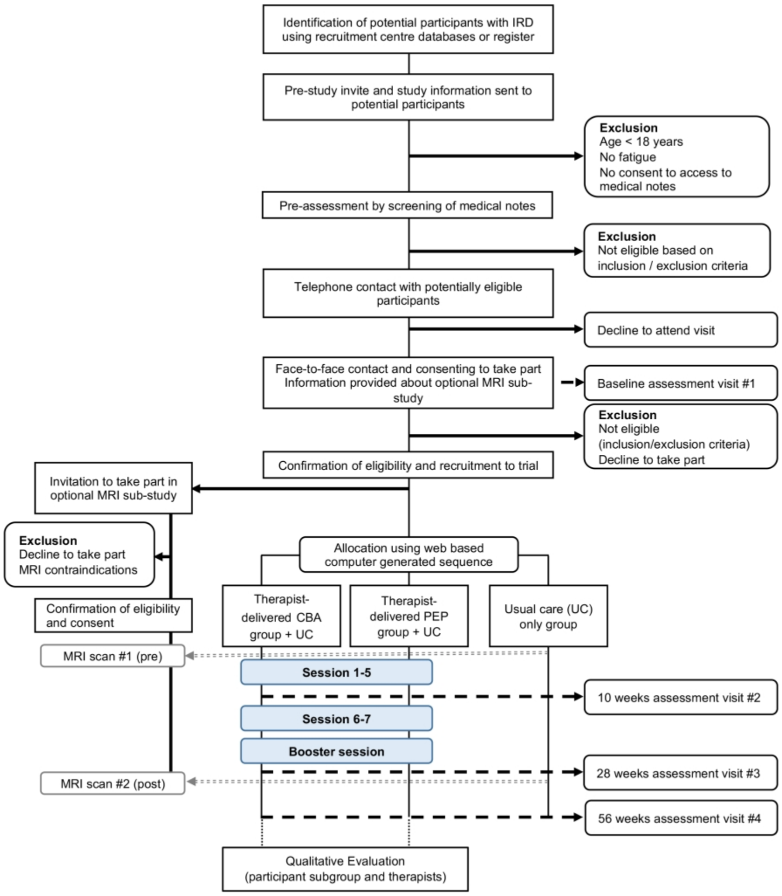
Identification of potential participants with IRD using recruitment centre databases or register
Pre-study invite and study information sent to potential participants
Exclusion
Age < 18 years
No fatigue
No consent to access to medical notes
Pre-assessment by screening of medical notes
Exclusion
Not eligible based on inclusion / exclusion criteria
Telephone contact with potentially eligible participants
Decline to attend visit
Face-to-face contact and consenting to take part
Information provided about optional MRI sub-study
Baseline assessment visit #1
Exclusion
Not eligible
(inclusion/exclusion criteria)
Decline to take part
Confirmation of eligibility and recruitment to trial
Invitation to take part in optional MRI sub-study
Exclusion
Decline to take part
MRI contraindications
Confirmation of eligibility and consent
Allocation using web based computer generated sequence
Therapist-delivered CBA group + UC
Therapist-delivered PEP group + UC
Usual care (UC) only group
MRI scan #1 (pre)
Session 1-5
10 weeks assessment visit #2
Session 6-7
Booster session
28 weeks assessment visit #3
MRI scan #2 (post)
56 weeks assessment visit #4
Qualitative Evaluation
(participant subgroup and therapists)

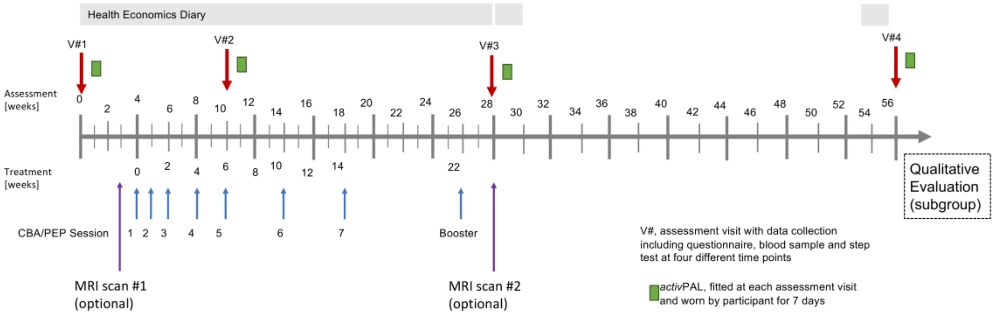
Health Economics Diary
V#1
V#2
V#3
V#4
Assessment [weeks]
0 2 4 6 8 10 12 14 16 18 20 22 24 26 28 30 32 34 36 38 40 42 44 46 48 50 52 54 56
Treatment [weeks]
0 2 4 6 8 10 12 14 22
CBA/PEP Session
1 2 3 4 5 6 7 Booster
MRI scan #1 (optional)
MRI scan #2 (optional)
Qualitative Evaluation (subgroup)
V#, assessment visit with data collection including questionnaire, blood sample and step test at four different time points
activPAL, fitted at each assessment visit and worn by participant for 7 days

Figure 3. Example template of recommended content for the schedule of enrolment, interventions, and assessments.

	STUDY PERIOD					
	Enrolment	Allocation	Post-allocation			Qualitative Evaluation
**TIMEPOINT**	*Pre-study*	*Visit 1 Baseline*	*Visit 2 10 weeks*	*Visit 3 28 weeks*	*Visit 4 56 weeks*	*Interview > 56 weeks*
**ENROLMENT:**						
**Pre-study invite**	X					
**Eligibility screen**	X	X				
**Informed consent**		X				X
**Allocation**		X				
**INTERVENTIONS[a]:**						
Usual Care alone			↔	↔		
CBA + Usual Care			↔	↔		
PEP + Usual Care			↔	↔		
**ASSESSMENTS:**						
Case Report Form	X	X	X	X	X	
Current fatigue status	X	X				
Medical history	X	X				
Comorbidities and pharmacological therapies (current)		X	X	X	X	
Participant reported outcomes[b]		X	X	X	X	
Aerobic fitness test (step test)		X	X	X	X	
Activity monitor		X	X	X	X	
Health Care Diary		↔	↔	↔	X	
Evaluation (quantitative)[c]			X	X		
Nested sub-study[d] Evaluation (qualitative)						X
Nested sub-study[d] MRI scan (optional)		X		X		

[a]Start of active CBA and PEP interventions expected to start between 2 and 8 weeks post-randomisation with an average of 4 weeks after baseline.

[b]Full list of questionnaires see outcomes.

[c]Full list of quantitative evaluation measures see Table 1 study matrix

[d]Invitation and consent separate from main trial

Table 1. Summary of outcome measures for each time point and their source (Study Matrix)

	Source	Items		**Proposed assessment [weeks]**			
			S	0	10	28	56
**Demographic data**							
Date of birth, gender, marital status, employment status, level of education	Q	5		✓			
**Characteristics of study population**							
Overall health	Q	1		✓			
Physical activity (typical self-reported)	Q	1		✓			
Experience of fatigue for more than 3 month	Q / CRF	1	✓	✓			
Average level of fatigue	Q / CRF	1	✓	✓			
Thyroid function test	B or MR			✓			
Urea and electrolytes	B or MR			✓			
Full blood count	B or MR			✓			
Serological status	MR			✓			
Erosive status	MR			✓			
Disease duration	MR			✓			
Presence of other co-morbidities (Charlson Index)	MR/CRF			✓	✓	✓	✓
History of suicide attempts	MR/CRF			✓			
Inflammation (CRP/ESR)	B			✓	✓	✓	✓
Current pharmacological therapies	MR			✓	✓	✓	✓
Blood pressure	T			✓			
**Primary Outcome**							
Chalder Fatigue Scale (Likert scoring)	Q	11		✓	✓	✓	✓
Fatigue Severity Scale	Q	9		✓	✓	✓	✓

For peer review

	Source	Items		Proposed assessment [weeks]			
			S	0	10	28	56
**Secondary Outcomes**							
Bristol Rheumatoid Arthritis Fatigue Multi-Dimensional Questionnaire (fatigue)	Q	20		✓	✓	✓	✓
Hospital anxiety and depression scale (anxiety and depression)	Q	14		✓	✓	✓	✓
Short Form-12	Q	12		✓	✓	✓	✓
Pain numerical rating scale	Q	1		✓	✓	✓	✓
Sleep problem scale	Q	4		✓	✓	✓	✓
Work Productivity and Activity Impairment Questionnaire	Q	6		✓	✓	✓	✓
Valued Life Activities Scale (short 14 items)	Q	14		✓	✓	✓	✓
Global outcome	Q	1			✓	✓	✓
**Additional mediator/moderator data**							
Cognitions and behaviours							
Brief Illness Perception Questionnaire	Q	9		✓	✓	✓	✓
Behavioural Response to Illness Questionnaire	Q	21		✓	✓	✓	✓
Clinical							
Presence of fibromyalgia	CRF	8		✓			✓
Disease activity (self-reported)	CRF	2		✓	✓	✓	✓
Disease activity DAS28 for RA (mandatory), other disease specific activity measures (non-mandatory)				✓	✓	✓	✓
Physical							
Physical activity profiles, over a 7 day period	T			✓	✓	✓	✓

For peer review

	Source	Items		Proposed assessment [weeks]			
			S	0	10	28	56
Quantifying aerobic fitness (step) test (weight, VO_2 max and Borg Rating of Perceived Exertion)	T			✓	✓	✓	✓
Neuroimaging (optional)							
Multi-modal MRI scan	SC			x		x	
**Quantitative evaluation**							
Patient preference	Q	2		✓			
Patient adherence (attendance records)	SR			x	x	x	
Patient engagement and adherence (telephone call)	Q	3			x	x	
Patent engagement and adherence (therapist perspective)	Q	2			x	x	
Patient acceptability (Client Satisfaction Questionnaire)	Q	8				✓	
**Qualitative process evaluation**							
Qualitative evaluation in participants (5% sample)	I						
Qualitative evaluation in therapists (all)	I						
**Economic evaluation**							
Health care costs from participants per diaries	D			✓	✓	✓	✓
Additional (personal) costs for participant per diaries	D			✓	✓	✓	✓
Cost associated with delivery of therapy	SR			✓	✓	✓	✓
Health related quality of life using SF-12 for calculation of QALY				✓	✓	✓	✓
Changes in well-being data using ICECAP	Q	5		✓	✓	✓	✓
Changes in life satisfaction	Q	1		✓	✓	✓	✓

For peer review

	Source	Items		Proposed assessment [weeks]			
			S	0	10	28	56
**Optional blood sample for future ethically approved research**							
1 x PAXgene RNA				✓	✓	✓	
1 x PAXgene DNA				✓			
1 x serum				✓	✓	✓	

Key: B, blood sample taken specifically for LIFT; **CRF**, part of case report form completed by research nurse during visit; **D**, separate diary for participant to keep; **I**, interviews performed after the last follow-up visit; **ICECAP**, Investigating Choice Experiments for the preference of older people CAPability measures for adults; **MR**, information extracted from medical record; **Q**, data derived from questionnaire; **QALY**, Quality Adjusted Life Years; **S**, information obtained during pre-study invite; **SC**, Scanner; **SR**, information extracted from LIFT study records and logs; **T**, test done specifically for LIFT; **x**, outcome collection at same time frame but separate from assessment visit

For peer review only

Additional File 1 SWAT 24

Background

Recruiting and retaining participants for randomised trials can be extremely difficult. It is likely that less than 50% of trials meet their recruitment target, or meet their target without extending the length of the trial [1-3]. Moreover, poor recruitment can lead to an underpowered study, which may report clinically relevant effects to be statistically non-significant. A non-significant finding increases the risk that an effective intervention will be abandoned before its true value is established, or that there will be a delay in demonstrating this value while more studies or meta-analyses are done. Moreover, if non-responses to the study invitation differ between the patients with diagnosis of RA, SLE and AxSpA, a systematic bias may be introduced that may undermine confidence in the results of the trial. Finally, poor recruitment and subsequently retention can lead to a trial being extended, increasing costs.

Trialists recognise the challenge and use many interventions to improve recruitment and retention but it is generally difficult to predict their effect. The Cochrane systematic review of strategies to improve recruitment [4] and the Cochrane review of strategies it improve retention [5] both found only a handful of interventions with high quality evidence of benefit. Given how central recruitment and retention are to all trials, it is crucial that more rigorous evaluations of recruitment and retention interventions are done.

Rationale for SWAT

One way of doing this is to do a Study Within A Trial (SWAT) [6]. A SWAT provides a protocol for the evaluation of an intervention to improve some part of the trial process, such as recruitment or retention. This evaluation is then embedded within a host trial, such as LIFT. Several teams can follow the same SWAT protocol, meaning the results can be combined in a meta-analysis. This coordinated and collaborative approach means trialists will have faster access to high-quality evidence to inform their trial design, conduct, analysis and reporting decisions.

The SWAT 24 which describes the use of a theory-based cover letter was initially developed to increase response rates of questionnaires sent during follow-up to collect outcome data direct from participants. A low response rate to these questionnaires puts the validity and generalisability of the trial results in jeopardy. Since returning the questionnaire is a behaviour, this opens up the possibility of designing a behaviour change intervention to influence the willingness of participants to do that behaviour. We propose to use SWAT 24 in LIFT to improve response rates to the pre-study invitation letter used to make initial contact with potential participants identified as described in the main protocol to explore interest and eligibility. The SWAT 24 study is part of the Trial Forge initiative to improve trial efficiency [7].

Objective for SWAT 24

To assess the effects of a theory-based cover letter on response rate to a pre-study invite to explore interest and eligibility

Outcome

Primary outcome:

For peer review only - http://bmjopen.bmj.com/site/about/guidelines.xhtml

Response rate

Secondary outcomes:

Response time

Consent rate

Study retention

Intervention adherence

Intervention

The Theoretical Domains Framework (TDF) is a tool for identifying theoretical targets for behaviour change interventions [8]. The TDF and behaviour change techniques were used by the IQuaD trial team [9] to produce a template that trial teams can use to structure a theory-informed cover letter.

Comparator

A standard cover letter

Method for allocating to intervention or comparator

Participating study centres will be randomly allocated to send the standard letter or the theory-informed letter.

Analysis plan

The primary analysis is the difference in primary and secondary outcomes between those receiving the theory-based cover letter and those receiving the standard cover letter.

References

1. Sully BGO, Julious SA, Nicholl J. A reinvestigation of recruitment to randomised, controlled, multicenter trials: a review of trials funded by two UK funding agencies. *Trials* 2013, 14(1): 166.
2. McDonald AM, Knight RC, Campbell MK, Entwistle VA, Grant AM, Cook JA, Elbourne DR, Francis D, Garcia J, Roberts I, Snowdon C. What influences recruitment to randomised controlled trials? A review of trials funded by two UK funding agencies. *Trials* 2006;7(1):9.
3. Foy R, Parry J, Duggan A, Delaney B, Wilson S, Lewin-van den Broek NTh, Lassen A, Vickers L, Myres P. How evidence-based are recruitment strategies for randomized controlled trials in primary care? Experience from seven studies. *Family Practice* 2003;20(1):83-92.
4. Treweek S, Mitchell E, Pitkethly M, Cook J, Kjeldstrøm M, Johansen M, Taskila TK, Sullivan F, Wilson S, Jackson C, Jones R, Lockhart P. Strategies to improve recruitment to randomised controlled trials. The Cochrane Library. 2010.

For peer review only

5. Brueton VC, Tierney J, Stenning S, Harding S, Meredith S, Nazareth I, Rait G. Strategies to improve retention in randomised trials. The Cochrane Database of Systematic Reviews. 2013;12:1.
6. Smith V, Clarke M, Devane D, Begley C, Shorter G, Maguire L. SWAT 1: what effects do site visits by the principal investigator have on recruitment in a multicentre randomized trial? *J Evid Based Med* 2013; 6(3):136–7.
7. Treweek S, Altman DG, Bower P, Campbell M, Chalmers I, Cotton S, et al. Making randomised trials more efficient: report of the first meeting to discuss the Trial Forge platform. *Trials* 2015; 16(1):261.
8. Michie et al. Making psychological theory useful for implementing evidence based practice. *BMJ Quality & Safety.* 2005; 14(1):26–33
9. Clarkson JE, Ramsay CR, Averley P, Bonetti D, Boyers D, Campbell L, Chadwick GR, Duncan A, Elders A, Gouick J, Hall AF et al. IQuaD dental trial; improving the quality of dentistry: a multicentre randomised controlled trial comparing oral hygiene advice and periodontal instrumentation for the prevention and management of periodontal disease in dentate adults attending dental primary care. *BMC Oral Health.* 2013; 13(1):58.

For peer review only

Appendix 2: Delineating the neural mediators of inflammatory rheumatic disease (An optional LIFT sub-study)

Background

One of the objectives of the LIFT trial is to understand the mediators of treatment effect which will enable optimising of interventions and inferences to be made regarding the mechanisms of fatigue. We are collecting putative patient reported, clinical and physical activity profile mediator data. In addition we would like to provide the option for participants to provide their neuroimaging data by undertaking additional MRI scans of their brain.

Neuroimaging has provided consistent mechanistic insights into fatigue, reinforcing our epidemiological investigations of RA related fatigue which have identified strong associations with central factors such as mental health and cognitive dysfunctions but not peripheral measures of inflammation[4,5]. We were the first to test these methods in a fatigued chronic inflammatory disease cohort. We identified multiple neural correlates of fatigue in patients with vasculitis using different structural and functional magnetic resonance imaging (MRI) modes [6,7] and we have since observed similar fatigue specific findings in RA.

Our original studies were small (n=12) and cross-sectional but did implicate a potential role for a striato-thalamo-frontal network. Among fatigued vasculitis patients, we observed an apparent overuse of the cingulum and fornix white matter tracts, as identified by Diffusion Tensor Imaging (DTI), which coincided with the high functional activity within some of their source and destination grey matter structures, specifically the thalamus, medial globus pallidus, medial frontal and cingulate gyri and paracentral lobule.

Our most recent pilot was larger and although uncontrolled included a repeat multi-modal scan at 6 months that enabled the longitudinal evaluation of fatigue in the context of standard care (which did not include fatigue specific therapy). Of those n=54 completing follow up, n=22 reported modest, albeit clinically relevant, improvements in their fatigue. Interestingly, widespread reductions in cortical grey matter volumes were measured, using voxel-based morphometry (VBM), among the non-improvers at follow-up however no such volume changes were observed among improvers. In contrast, sub-cortical grey matter volumes exhibited large significant increases in non-improvers. The sub-cortical grey matter volumes of improvers also increased, although the changes were small in comparison.

In terms of white matter integrity, as measured by DTI, widespread abnormalities were observed among improvers. Within this group, significantly reduced fractional anisotropy (FA) was measured at follow-up compared to baseline in major white matter tracts. Strikingly, no significant longitudinal FA changes were measured within the non-improvers group. Similarly, improvers- and not non-improvers- evidenced widespread imbalances in functional connectivity imbalance over time.

Overall, these data strongly associate central neural pathways in RA related fatigue. Although the different MRI metrics provide complementary evidence which implicate frontal networks, a targeted approach (e.g. with non-invasive neuromodulation devices) will demand much greater knowledge of the precise culprit frontal regions, moreover several non-frontal regions seem also to be relevant. Until now, no studies have been adequately designed to pin-point those brain regions which mediate changes in fatigue (and so demonstrate causal potential).

For peer review only

The problem with our existing studies is that they were either cross-sectional, or longitudinally followed individuals who had spontaneous changes in their fatigue (thus the changes in fatigue from one time point to the next were modest). We propose that the best way to identify which of these brain regions are most important in mediating fatigue is to perform controlled longitudinal imaging studies in individuals prior to and then following an intervention that reliably improves fatigue in most individuals.

This Lessening the Impact of Fatigue Trial (LIFT) sub-study provides a timely opportunity to address this research void.

Sub-study objective

- Which functional and structural brain mediators best explain fatigue improvement and are they potentially trans-cranially accessible?

Study design

An optional nested mechanistic observational sub-study within the Lessening the Impact of Fatigue Trial (LIFT).

Participants

All consenting LIFT participants will be invited to participate in this optional sub-study which involves an additional MRI brain scan prior to and 26 weeks after their first treatment session (if CBA or PEP) or within 8 weeks of randomisation and 6 months thereafter if usual care. The only additional exclusion criteria is any contra-indications to MRI scanning (e.g. pacemaker).

Participant selection

At the baseline visit, all participants will be provided a Participant Information Sheet on this sub-study. The research team will then contact the participants a few days later to establish interest and the absence of MRI contraindications. If suitable and interested, the participant will be offered an appointment to attend their nearest participating MRI research facility (Glasgow, Edinburgh or Aberdeen) within a month. A research team member, recorded in the Delegation Log and with GCP training, will be responsible for taking additional full written informed consent (i.e. specific to this sub-study) on attendance at the imaging centre prior to the MRI assessment and then conduct a final MRI safety screen.

Participant withdrawal

All participants will be free to withdraw at any time from the MRI sub-study, without giving reasons and without prejudicing further treatment or their participation in the LIFT trial.

For peer review only

MRI assessment

Prior to entering the scanner, subjects will have the opportunity to practice a cognitive task required for the standard fMRI aspect of analysis. As with our previous work[6], the validated PASAT will be employed to transiently fatigue the subject. The task is a measure of cognitive function; specifically auditory processing, calculation, working memory and attention. Participants will be asked to listen to a series of numbers ranging from 1 to 9. They are required to sum consecutive numbers (i.e. the first to the second, the second to the third etc.) and to record, via a button press, every occasion two consecutive numbers sum to the number 10. Concurrently, they will be asked to focus on a computer screen displaying three boxes containing random, rapidly changing numbers. This visual stimulus is intended to distract the participants from the auditory task and hence increase difficulty. They will be instructed not to process the visual numbers in any way.

Participants will then be asked to lie supine in the 3T Phillips Achieva X-series MRI scanner in Aberdeen or the equivalent scanner in Edinburgh or Glasgow. The multi-modal MR will consist of structural and functional sequences:

Structural: We will collect images to allow volumetric analysis. We will also acquire images to allow determination of white matter hyperintensity lesion load and measures of white matter structural integrity (e.g. DTI).

Functional Imaging: Images sensitive to BOLD contrast will be acquired during rest to investigate metrics such as intrinsic network connectivity as well as during the PASAT task (3x3minute periods interspersed by 30s rest periods).

In total these can will take approximately 45 minutes to conduct and will be repeated at approximately 6 months (when we predict to observe the greatest effect from the interventions).

Analysis

Following pre-processing of the MRI data the following analysis will be undertaken which will integrate data which will have been collected as part of the parent trial:

Longitudinal comparisons (paired t-tests as implemented by SPSS for ROI based variables and Freesurfer for voxel based variables) of structural and functional change indices in relation to subjects' change in fatigue will be performed. Putative confounders will be individually introduced as co-variates of interest. The individual analyses will focus upon those regions of interest previously identified by our studies, but since we recognise that these are not comprehensive we will also conduct agnostic (data-driven) whole brain analyses. All analyses will be adjusted for multiple testing.

Having identified and validated key neural areas, group differences in mediators of treatment effects on outcomes will be assessed via mediation analysis methods in order to tackle the secondary objective. These involve causal inference methods, such as structural equation modelling, to account for measurement error in the imaging data, and repeated measures to allow for the inclusion of all available data. Those resultant neural mediators which are common to both interventions and accessible to non-invasive neuromodulation will serve as our future therapeutic targets.

Finally we will be using whole brain statistical pattern recognition techniques on the neuroimaging data and mediation effects identified in answer to the previous objective. In the Pattern Recognition for Neuroimaging Toolbox (PRoNTo) brain scans are treated as spatial patterns and statistical

For peer review only

learning models are used to identify statistical properties of the data that can be used to discriminate between, or classify, experimental groups of subjects.

Sample size

We aim to recruit 120 participants (who will have been randomised to receive either usual care alone, CBA in addition to usual care or PEP in addition to usual care in the parent trial).

Data handling

MRI scan data will be stored in an anonymised format in the University of Aberdeen and Edinburgh imaging archive system on the university drive, with a back-up disc stored in a fireproof safe. The code for the images will be held on a separate computer relating the patient information to the participant ID. The participant ID will be used on the MRI images. Images may be stored on disc in anonymised format for research team discussions out-with the imaging department.

Safety assessments

The MRI scanner is very safe and does not expose participants to any harmful radiation. Given its reliance on a strong magnetic field, it is essential that certain metallic instruments/objects are not taken into the scan room.

This is avoided by:

1) All participants are clothed in 'theatre greens' so to avoid the danger of concealed metal objects within clothes.

2) Patients will be screened for absolute exclusion to MRI scanning.

In addition, all participants must undertake a strict and comprehensive checklist prior to scanning. This includes questions about heart valves, pacemakers and other potential metallic implants.

If a participant becomes distressed during the MRI scan, he/she will be able to access to a “panic button” which will immediately terminate the procedure.

For peer review only

Sub-study matrix

The study connects to the 1-year LIFT recruitment phase which will begin in August 2017 (month 0).

Study steps	-3–0 m	0–6 m	6–12 m	12–18 m	18–24 m	24–30 m
Governance approvals						
RA Subject recruitment						
MRI scan #1 (pre-intervention)						
MRI scan #2 (post-intervention)						
Data processing, analysis & report						

References

1. Hewlett S, Cockshott Z, Byron M, et al. Patients' perceptions of fatigue in rheumatoid arthritis: overwhelming, uncontrollable, ignored. *Arthritis and Rheumatism.* 2005;53(5):697-702.
2. Davies H, Brophy S, Dennis M, Cooksey R, Irvine E, Siebert S. Patient perspectives of managing fatigue in Ankylosing Spondylitis, and views on potential interventions: a qualitative study. *BMC Musculoskelet Disord.* 2013;14(1):163.
3. Patton MQ. *Qualitative Research & Evaluation Methods.* 3rd ed. Thousand Oaks, Calif. ; London: Sage; 2002.
4. Druce KL, Jones GT, Macfarlane GJ, Basu N. Determining pathways to improvements in fatigue in rheumatoid arthritis: results from the British Society for Rheumatology Biologics Register for Rheumatoid Arthritis. *Arthritis & Rheumatology.* 2015;67:2303-2310.
5. Druce KL, Jones GT, Macfarlane GJ, Basu N. The changes in pain which mediate Reductions in fatigue likely reflect central not peripheral mechanisms: Results from the BSRBR-RA. *Arthritis Care & Research.* 2016; 68(7):922-6.
6. Basu N, Murray AD, Jones GT, Reid DM, Macfarlane GJ, Waiter GD. Neural correlates of fatigue in granulomatosis with polyangiitis: a functional magnetic resonance imaging study. *Rheumatology.* 2014;53:2080-2087.
7. Basu N, Murray AD, Jones GT, Reid DM, Macfarlane GJ, Waiter GD. Fatigue-related brain white matter changes in granulomatosis with polyangiitis. *Rheumatology.* 2013; 52(8):1429-34.

For peer review only

SPIRIT

STANDARD PROTOCOL ITEMS: RECOMMENDATIONS FOR INTERVENTIONAL TRIALS

SPIRIT 2013 Checklist: Recommended items to address in a clinical trial protocol and related documents*

Section/item	Item No	Description	Addressed on page number
**Administrative information**			
Title	1	Descriptive title identifying the study design, population, interventions, and, if applicable, trial acronym	1
Trial registration	2a	Trial identifier and registry name. If not yet registered, name of intended registry	5
	2b	All items from the World Health Organization Trial Registration Data Set	n/a
Protocol version	3	Date and version identifier	31
Funding	4	Sources and types of financial, material, and other support	30
Roles and responsibilities	5a	Names, affiliations, and roles of protocol contributors	1-3, 31
	5b	Name and contact information for the trial sponsor	30
	5c	Role of study sponsor and funders, if any, in study design; collection, management, analysis, and interpretation of data; writing of the report; and the decision to submit the report for publication, including whether they will have ultimate authority over any of these activities	30
	5d	Composition, roles, and responsibilities of the coordinating centre, steering committee, endpoint adjudication committee, data management team, and other individuals or groups overseeing the trial, if applicable (see Item 21a for data monitoring committee)	26

For peer review only - http://bmjopen.bmj.com/site/about/guidelines.xhtml

For peer review only

**Introduction**			
Background and rationale	6a	Description of research question and justification for undertaking the trial, including summary of relevant studies (published and unpublished) examining benefits and harms for each intervention	5-8
	6b	Explanation for choice of comparators	5-8
Objectives	7	Specific objectives or hypotheses	8
Trial design	8	Description of trial design including type of trial (eg, parallel group, crossover, factorial, single group), allocation ratio, and framework (eg, superiority, equivalence, noninferiority, exploratory)	9
**Methods: Participants, interventions, and outcomes**			
Study setting	9	Description of study settings (eg, community clinic, academic hospital) and list of countries where data will be collected. Reference to where list of study sites can be obtained	9
Eligibility criteria	10	Inclusion and exclusion criteria for participants. If applicable, eligibility criteria for study centres and individuals who will perform the interventions (eg, surgeons, psychotherapists)	9-11
Interventions	11a	Interventions for each group with sufficient detail to allow replication, including how and when they will be administered	13-16
	11b	Criteria for discontinuing or modifying allocated interventions for a given trial participant (eg, drug dose change in response to harms, participant request, or improving/worsening disease)	n/a
	11c	Strategies to improve adherence to intervention protocols, and any procedures for monitoring adherence (eg, drug tablet return, laboratory tests)	16, 19
	11d	Relevant concomitant care and interventions that are permitted or prohibited during the trial	13
Outcomes	12	Primary, secondary, and other outcomes, including the specific measurement variable (eg, systolic blood pressure), analysis metric (eg, change from baseline, final value, time to event), method of aggregation (eg, median, proportion), and time point for each outcome. Explanation of the clinical relevance of chosen efficacy and harm outcomes is strongly recommended	16-20, Supplement Table 1
Participant timeline	13	Time schedule of enrolment, interventions (including any run-ins and washouts), assessments, and visits for participants. A schematic diagram is highly recommended (see Figure)	9, 16, Figure 1, Figure 2, Figure 3

For peer review only - http://bmjopen.bmj.com/site/about/guidelines.xhtml

Sample size	14	Estimated number of participants needed to achieve study objectives and how it was determined, including clinical and statistical assumptions supporting any sample size calculations	22
Recruitment	15	Strategies for achieving adequate participant enrolment to reach target sample size	9
**Methods: Assignment of interventions (for controlled trials)**			
Allocation:			
Sequence generation	16a	Method of generating the allocation sequence (eg, computer-generated random numbers), and list of any factors for stratification. To reduce predictability of a random sequence, details of any planned restriction (eg, blocking) should be provided in a separate document that is unavailable to those who enrol participants or assign interventions	12
Allocation concealment mechanism	16b	Mechanism of implementing the allocation sequence (eg, central telephone; sequentially numbered, opaque, sealed envelopes), describing any steps to conceal the sequence until interventions are assigned	12
Implementation	16c	Who will generate the allocation sequence, who will enrol participants, and who will assign participants to interventions	12
Blinding (masking)	17a	Who will be blinded after assignment to interventions (eg, trial participants, care providers, outcome assessors, data analysts), and how	12-13
	17b	If blinded, circumstances under which unblinding is permissible, and procedure for revealing a participant's allocated intervention during the trial	n/a
**Methods: Data collection, management, and analysis**			
Data collection methods	18a	Plans for assessment and collection of outcome, baseline, and other trial data, including any related processes to promote data quality (eg, duplicate measurements, training of assessors) and a description of study instruments (eg, questionnaires, laboratory tests) along with their reliability and validity, if known. Reference to where data collection forms can be found, if not in the protocol	16-21
	18b	Plans to promote participant retention and complete follow-up, including list of any outcome data to be collected for participants who discontinue or deviate from intervention protocols	22

For peer review only - http://bmjopen.bmj.com/site/about/guidelines.xhtml

Data management	19	Plans for data entry, coding, security, and storage, including any related processes to promote data quality (eg, double data entry; range checks for data values). Reference to where details of data management procedures can be found, if not in the protocol	27
Statistical methods	20a	Statistical methods for analysing primary and secondary outcomes. Reference to where other details of the statistical analysis plan can be found, if not in the protocol	22-26
	20b	Methods for any additional analyses (eg, subgroup and adjusted analyses)	24-25
	20c	Definition of analysis population relating to protocol non-adherence (eg, as randomised analysis), and any statistical methods to handle missing data (eg, multiple imputation)	25
**Methods: Monitoring**			
Data monitoring	21a	Composition of data monitoring committee (DMC); summary of its role and reporting structure; statement of whether it is independent from the sponsor and competing interests; and reference to where further details about its charter can be found, if not in the protocol. Alternatively, an explanation of why a DMC is not needed	26-27
	21b	Description of any interim analyses and stopping guidelines, including who will have access to these interim results and make the final decision to terminate the trial	n/a
Harms	22	Plans for collecting, assessing, reporting, and managing solicited and spontaneously reported adverse events and other unintended effects of trial interventions or trial conduct	21
Auditing	23	Frequency and procedures for auditing trial conduct, if any, and whether the process will be independent from investigators and the sponsor	n/a
**Ethics and dissemination**			
Research ethics approval	24	Plans for seeking research ethics committee/institutional review board (REC/IRB) approval	31-32
Protocol amendments	25	Plans for communicating important protocol modifications (eg, changes to eligibility criteria, outcomes, analyses) to relevant parties (eg, investigators, REC/IRBs, trial participants, trial registries, journals, regulators)	n/a

For peer review only - http://bmjopen.bmj.com/site/about/guidelines.xhtml

Consent or assent	26a	Who will obtain informed consent or assent from potential trial participants or authorised surrogates, and how (see Item 32)	12
	26b	Additional consent provisions for collection and use of participant data and biological specimens in ancillary studies, if applicable	19, 20
Confidentiality	27	How personal information about potential and enrolled participants will be collected, shared, and maintained in order to protect confidentiality before, during, and after the trial	27
Declaration of interests	28	Financial and other competing interests for principal investigators for the overall trial and each study site	31
Access to data	29	Statement of who will have access to the final trial dataset, and disclosure of contractual agreements that limit such access for investigators	30
Ancillary and post-trial care	30	Provisions, if any, for ancillary and post-trial care, and for compensation to those who suffer harm from trial participation	n/a
Dissemination policy	31a	Plans for investigators and sponsor to communicate trial results to participants, healthcare professionals, the public, and other relevant groups (eg, via publication, reporting in results databases, or other data sharing arrangements), including any publication restrictions	30
	31b	Authorship eligibility guidelines and any intended use of professional writers	n/a
	31c	Plans, if any, for granting public access to the full protocol, participant-level dataset, and statistical code	30
**Appendices**			
Informed consent materials	32	Model consent form and other related documentation given to participants and authorised surrogates	n/a
Biological specimens	33	Plans for collection, laboratory evaluation, and storage of biological specimens for genetic or molecular analysis in the current trial and for future use in ancillary studies, if applicable	18-19

*It is strongly recommended that this checklist be read in conjunction with the SPIRIT 2013 Explanation & Elaboration for important clarification on the items. Amendments to the protocol should be tracked and dated. The SPIRIT checklist is copyrighted by the SPIRIT Group under the Creative Commons "Attribution-NonCommercial-NoDerivs 3.0 Unported" license.

For peer review only - http://bmjopen.bmj.com/site/about/guidelines.xhtml
