# Supplementary material for: Protocol for a multicentre randomised controlled parallel-group trial to compare the effectiveness of remotely delivered cognitive-behavioural and graded exercise interventions with usual care alone to lessen the impact of fatigue in inflammatory rheumatic diseases (LIFT)
Source: BMJ Open. 2019 Jan 30;9(1):e026793. doi: 10.1136/bmjopen-2018-026793 (PMC6359876; doi:10.1136/bmjopen-2018-026793)
Supplement: Reviewer comments [file bmjopen-2018-026793.reviewer_comments.pdf]

PEER REVIEW HISTORY

BMJ Open publishes all reviews undertaken for accepted manuscripts. Reviewers are asked to complete a checklist review form (**http://bmjopen.bmj.com/site/about/resources/checklist.pdf**) and are provided with free text boxes to elaborate on their assessment. These free text comments are reproduced below.

ARTICLE DETAILS

**TITLE (PROVISIONAL)**	Protocol for a multi-centre randomised controlled parallel-group trial to compare the effectiveness of remotely delivered cognitive behavioural and graded exercise interventions with usual care alone to lessen the impact of fatigue in inflammatory rheumatic diseases (LIFT)
**AUTHORS**	Martin, Kathryn; Bachmair, Eva-Maria; Aucott, Lorna; Dures, Emma; Emsley, Richard; Gray, Stuart; Hewlett, Sarah; Kumar, Vinod; Lovell, Karina; Macfarlane, Gary; Maclennan, Graeme; McNamee, Paul; Norrie, John; Paul, Lorna; Ralston, Stuart; Siebert, Stefan; Wearden, Alison; White, Peter; Basu, Neil

This article was not externally reviewed at BMJ Open. Protocols that have been independently assessed prior to submission to BMJ Open are usually fast-tracked to publication on the grounds that further substantial changes will not be possible. This independent assessment will usually be external, independent review for both a substantial grant award from a non-commercial or government funder and ethics approval.
